# Consensus guidelines for the use and interpretation of angiogenesis assays

**DOI:** 10.1007/s10456-018-9613-x

**Published:** 2018-08

**Authors:** Patrycja Nowak-Sliwinska, Kari Alitalo, Elizabeth Allen, Andrey Anisimov, Alfred C. Aplin, Robert Auerbach, Hellmut G. Augustin, David O. Bates, Judy R. van Beijnum, R. Hugh F. Bender, Gabriele Bergers, Andreas Bikfalvi, Joyce Bischoff, Barbara C. Böck, Peter C. Brooks, Federico Bussolino, Bertan Cakir, Peter Carmeliet, Daniel Castranova, Anca M. Cimpean, Ondine Cleaver, George Coukos, George E. Davis, Michele De Palma, Anna Dimberg, Ruud P. M. Dings, Valentin Djonov, Andrew C. Dudley, Neil P. Dufton, Sarah-Maria Fendt, Napoleone Ferrara, Marcus Fruttiger, Dai Fukumura, Bart Ghesquière, Yan Gong, Robert J. Griffin, Adrian L. Harris, Christopher C. W. Hughes, Nan W. Hultgren, M. Luisa Iruela-Arispe, Melita Irving, Rakesh K. Jain, Raghu Kalluri, Joanna Kalucka, Robert S. Kerbel, Jan Kitajewski, Ingeborg Klaassen, Hynda K. Kleinmann, Pieter Koolwijk, Elisabeth Kuczynski, Brenda R. Kwak, Koen Marien, Juan M. Melero-Martin, Lance L. Munn, Roberto F. Nicosia, Agnes Noel, Jussi Nurro, Anna-Karin Olsson, Tatiana V. Petrova, Kristian Pietras, Roberto Pili, Jeffrey W. Pollard, Mark J. Post, Paul H. A. Quax, Gabriel A. Rabinovich, Marius Raica, Anna M. Randi, Domenico Ribatti, Curzio Ruegg, Reinier O. Schlingemann, Stefan Schulte-Merker, Lois E. H. Smith, Jonathan W. Song, Steven A. Stacker, Jimmy Stalin, Amber N. Stratman, Maureen Van de Velde, Victor W. M. van Hinsbergh, Peter B. Vermeulen, Johannes Waltenberger, Brant M. Weinstein, Hong Xin, Bahar Yetkin-Arik, Seppo Yla-Herttuala, Mervin C. Yoder, Arjan W. Griffioen

**Affiliations:** 1 Molecular Pharmacology Group, School of Pharmaceutical Sciences, Faculty of Sciences, University of Geneva, University of Lausanne, Rue Michel-Servet 1, CMU, 1211 Geneva 4, Switzerland; 2 Translational Research Center in Oncohaematology, University of Geneva, Geneva, Switzerland; 3 Wihuri Research Institute and Translational Cancer Biology Program, University of Helsinki, Helsinki, Finland; 4 Laboratory of Tumor Microenvironment and Therapeutic Resistance, Department of Oncology, VIB-Center for Cancer Biology, KU Leuven, Louvain, Belgium; 5 Department of Pathology, University of Washington, Seattle, WA, USA; 6 University of Wisconsin, Madison, WI, USA; 7 European Center for Angioscience, Medical Faculty Mannheim, Heidelberg University, Heidelberg, Germany; 8 Division of Vascular Oncology and Metastasis Research, German Cancer Research Center, Heidelberg, Germany; 9 German Cancer Consortium, Heidelberg, Germany; 10 Division of Cancer and Stem Cells, School of Medicine, University of Nottingham, Nottingham, UK; 11 Angiogenesis Laboratory, Department of Medical Oncology, VU University Medical Center, Cancer Center Amsterdam, De Boelelaan 1117, 1081 HV Amsterdam, The Netherlands; 12 Department of Molecular Biology and Biochemistry, University of California, Irvine, CA, USA; 13 Department of Neurological Surgery, Brain Tumor Research Center, Helen Diller Family Comprehensive Cancer Center, University of California, San Francisco, CA, USA; 14 Angiogenesis and Tumor Microenvironment Laboratory (INSERM U1029), University Bordeaux, Pessac, France; 15 Vascular Biology Program and Department of Surgery, Harvard Medical School, Boston Children’s Hospital, Boston, MA, USA; 16 Center for Molecular Medicine, Maine Medical Center Research Institute, Scarborough, ME, USA; 17 Department of Oncology, University of Torino, Turin, Italy; 18 Candiolo Cancer Institute-FPO-IRCCS, 10060 Candiolo, Italy; 19 Department of Ophthalmology, Harvard Medical School, Boston Children’s Hospital, Boston, MA, USA; 20 Laboratory of Angiogenesis and Vascular Metabolism, Department of Oncology and Leuven Cancer Institute (LKI), KU Leuven, Leuven, Belgium; 21 Laboratory of Angiogenesis and Vascular Metabolism, Center for Cancer Biology, VIB, Leuven, Belgium; 22 Division of Developmental Biology, Eunice Kennedy Shriver National Institute of Child Health and Human Development, National Institutes of Health, Bethesda, MD, USA; 23 Department of Microscopic Morphology/Histology, Angiogenesis Research Center, Victor Babes University of Medicine and Pharmacy, Timisoara, Romania; 24 Department of Molecular Biology, Center for Regenerative Science and Medicine, University of Texas Southwestern Medical Center, Dallas, TX, USA; 25 Ludwig Institute for Cancer Research, Department of Oncology, University of Lausanne, Lausanne, Switzerland; 26 Department of Medical Pharmacology and Physiology, University of Missouri, School of Medicine and Dalton Cardiovascular Center, Columbia, MO, USA; 27 School of Life Sciences, Swiss Federal Institute of Technology, Lausanne, Switzerland; 28 Department of Immunology, Genetics and Pathology, Uppsala University, Uppsala, Sweden; 29 Department of Radiation Oncology, University of Arkansas for Medical Sciences, Little Rock, AR, USA; 30 Institute of Anatomy, University of Bern, Bern, Switzerland; 31 Department of Microbiology, Immunology, and Cancer Biology, University of Virginia, Charlottesville, VA, USA; 32 Emily Couric Cancer Center, The University of Virginia, Charlottesville, VA, USA; 33 Vascular Sciences, Imperial Centre for Translational and Experimental Medicine, National Heart and Lung Institute, Imperial College London, London, UK; 34 Laboratory of Cellular Metabolism and Metabolic Regulation, VIB Center for Cancer Biology, Leuven, Belgium; 35 Laboratory of Cellular Metabolism and Metabolic Regulation, Department of Oncology, KU Leuven and Leuven Cancer Institute, Leuven, Belgium; 36 University of California, San Diego, La Jolla, CA, USA; 37 Institute of Ophthalmology, University College London, London, UK; 38 Edwin L. Steele Laboratories, Department of Radiation Oncology, Massachusetts General Hospital and Harvard Medical School, Boston, MA, USA; 39 Metabolomics Expertise Center, VIB Center for Cancer Biology, VIB, Leuven, Belgium; 40 Department of Oncology, Metabolomics Expertise Center, KU Leuven, Leuven, Belgium; 41 Molecular Oncology Laboratories, Oxford University Department of Oncology, Weatherall Institute of Molecular Medicine, John Radcliffe Hospital, Oxford, UK; 42 MCDB, University of California, Los Angeles, CA, USA; 43 Department of Cancer Biology, Metastasis Research Center, The University of Texas MD Anderson Cancer Center, Houston, TX, USA; 44 Department of Medical Biophysics, Biological Sciences Platform, Sunnybrook Research Institute, University of Toronto, Toronto, ON, Canada; 45 Department of Physiology and Biophysics, University of Illinois, Chicago, IL, USA; 46 Ocular Angiogenesis Group, Departments of Ophthalmology and Medical Biology, Academic Medical Center, University of Amsterdam, Amsterdam, The Netherlands; 47 The George Washington University School of Medicine, Washington, DC, USA; 48 Department of Ophthalmology, University of Lausanne, Jules-Gonin Eye Hospital, Fondation Asile des Aveugles, Lausanne, Switzerland; 49 Department of Pathology and Immunology, University of Geneva, Geneva, Switzerland; 50 HistoGeneX, Antwerp, Belgium; 51 Department of Cardiac Surgery, Harvard Medical School, Boston Children’s Hospital, Boston, MA, USA; 52 Pathology and Laboratory Medicine Service, VA Puget Sound Health Care System, Seattle, WA, USA; 53 Laboratory of Tumor and Developmental Biology, GIGA-Cancer, University of Liège, Liège, Belgium; 54 Department of Biotechnology and Molecular Medicine, University of Eastern Finland, Kuopio, Finland; 55 Department of Medical Biochemistry and Microbiology, Science for Life Laboratory, Uppsala Biomedical Center, Uppsala University, Uppsala, Sweden; 56 Department of oncology UNIL-CHUV, Ludwig Institute for Cancer Research Lausanne, Lausanne, Switzerland; 57 Division of Translational Cancer Research, Department of Laboratory Medicine, Lund, Sweden; 58 Genitourinary Program, Indiana University-Simon Cancer Center, Indianapolis, IN, USA; 59 Medical Research Council Centre for Reproductive Health, College of Medicine and Veterinary Medicine, University of Edinburgh, Edinburgh, UK; 60 Department of Physiology, Maastricht University, Maastricht, The Netherlands; 61 Einthoven Laboratory for Experimental Vascular Medicine, Department Surgery, LUMC, Leiden, The Netherlands; 62 Laboratory of Immunopathology, Institute of Biology and Experimental Medicine, National Council of Scientific and Technical Investigations (CONICET), Buenos Aires, Argentina; 63 Department of Basic Medical Sciences, Neurosciences and Sensory Organs, University of Bari Medical School, Bari, Italy; 64 National Cancer Institute “Giovanni Paolo II”, Bari, Italy; 65 Department of Oncology, Microbiology and Immunology, Faculty of Science and Medicine, University of Fribourg, Fribourg, Switzerland; 66 Institute of Cardiovascular Organogenesis and Regeneration, Faculty of Medicine, WWU, Münster, Germany; 67 Department of Mechanical and Aerospace Engineering, The Ohio State University, Columbus, OH, USA; 68 Comprehensive Cancer Center, The Ohio State University, Columbus, OH, USA; 69 Tumour Angiogenesis and Microenvironment Program, Peter MacCallum Cancer Centre and The Sir Peter MacCallum, Department of Oncology, University of Melbourne, Melbourne, VIC, Australia; 70 Medical Faculty, University of Münster, Albert-Schweitzer-Campus 1, Münster, Germany; 71 Department of Pediatrics, Indiana University School of Medicine, Indianapolis, IN, USA; 72 Translational Cancer Research Unit, GZA Hospitals, Sint-Augustinus & University of Antwerp, Antwerp, Belgium

**Keywords:** Angiogenesis, Aortic ring, Endothelial cell migration, Proliferation, Microfluidic, Zebrafish, Chorioallantoic membrane (CAM), Vascular network, Intussusceptive angiogenesis, Retinal vasculature, Corneal angiogenesis, Hindlimb ischemia, Myocardial angiogenesis, Recombinant proteins, Tip cells, Plug assay, Myocardial angiogenesis, Vessel co-option

## Abstract

The formation of new blood vessels, or angiogenesis, is a complex process that plays important roles in growth and development, tissue and organ regeneration, as well as numerous pathological conditions. Angiogenesis undergoes multiple discrete steps that can be individually evaluated and quantified by a large number of bioassays. These independent assessments hold advantages but also have limitations. This article describes in vivo, ex vivo, and in vitro bioassays that are available for the evaluation of angiogenesis and highlights critical aspects that are relevant for their execution and proper interpretation. As such, this collaborative work is the first edition of consensus guidelines on angiogenesis bioassays to serve for current and future reference.

## 1 Introduction

The process of angiogenesis—the formation of new blood vessels from preexisting ones—is a hallmark of tissue repair, expansion, and remodeling in physiological processes, such as wound healing, ovulation, and embryo development, and in various pathologies including cancer, atherosclerosis, and chronic inflammation [1–5]. Many of these conditions share characteristics, for example the occurrence of hypoxia or inflammation, recruitment of inflammatory cells, angiogenic growth factor production, basement membrane degradation, endothelial cell (EC) migration, proliferation and differentiation, and modulation of vascular support cells. However, depending on the tissue or disease under investigation, important details may differ considerably. Moreover, EC in different vascular beds exhibits organ-specific heterogeneity associated with the differentiated specialized functions of the tissue. It is often not possible to accurately visualize the process of angiogenesis and its molecular players. Therefore, different in vivo, ex vivo, and in vitro bioassays and techniques have been developed to investigate the specific stages of the angiogenesis. However, the use of bioassays that study a part of the process, with the intention to extrapolate and understand the full process of angiogenesis, inherently implies accepting specific limitations. It is therefore crucial to understand the full potential of these bioassays during their specific applications. These assays have been instrumental in the study of vascular biology in growth and development [6–8] but also play a key role in the design, development, and evaluation of drugs that positively or negatively modulate vessel function for the treatment of many diseases [9–11]. Some examples of where the use of such bioassays has been imperative are: (1) the development of angiostatic drugs for the treatment of cancer, ocular diseases, and other pathologic conditions where angiogenesis is implicated and also angiogenic treatment strategies in ischemic cardiovascular disease [12, 13], (2) screening of natural anti-angiogenic compounds [14], (3) the efforts to design combination therapies including angiogenesis inhibitors [15–20], (4) the unraveling of mechanisms regulating lymphangiogenesis [21, 22], (5) the interrelationship of angiogenesis and immunity [23–25], (6) the development of imaging as diagnostic strategy [26], (7) the study of drug resistance mechanisms [27–29], (8) development of compounds and strategies for the revascularization of ischemic injuries [30, 31], and (9) to improve the vascular fitness in aging vessels [32, 33]. The current paper describes a large collection of assays and techniques for the evaluation of angiogenesis and aims at explaining their respective advantages and limitations. In addition, we included strategies to study angiogenesis in tissues, through means of assessing and quantifying microvessel density (MVD), vessel co-option, pericyte coverage, and tip cell behavior.

## 2 Endothelial cell and monocyte migration assays

EC migration is one of the hallmarks of angiogenesis and one of the earlier steps in the angiogenic cascade. This process is characterized by cell-autonomous motility property but in some cases, it acquires the features of collective migration [34–37], in which a group of cells coordinate their movements toward a chemotactic gradient and by establishing a precise hierarchy with leader and follower cells. Therefore, dissection of the molecular mechanisms of EC migration is critical to understand and to therapeutically manipulate the process to either inhibit sprouting (e.g., in tumors) or stimulate vessel formation (e.g., during tissue regeneration or wound healing).

Likewise, migration assays have been successfully used to assess the migratory responsiveness of monocytes. Monocytes are actively involved in angiogenesis, and their migratory response or potential correlates well with that of endothelial cells. Most importantly, CD14-positive monocytes can easily be isolated and obtained from any individual, not only humans [38, 39], but also mice [40]. A number of 2D and 3D cellular migration assays have been established as relatively simple in vitro readouts of the migratory/angiogenic activity of EC in response to exogenous stimuli. Depending on the specific scientific question, a range of assays is available to quantitatively and qualitatively assess EC migration. The most widely employed assays include variations of the wound closure and the Boyden chamber assays.

### 2.1 Types of assays

#### Cell culture wound closure assay

Lateral migration assays are performed to investigate the pro- or anti-migratory effect of compounds, as well of specific gene perturbations, or to describe phenotypes resulting from genetic manipulation of EC. Although these assays can be used to characterize chemokinesis (unidirectional migration) in response to a given compound added to the cell culture medium, they do not allow determination of directed migration rate toward or away from a compound. Assessment of chemotaxis can only be determined when a gradient is also provided.

The cell culture wound closure assay is one of the basic readouts for characterizing the migratory activity of cells. It is a measure of the lateral 2D migration of EC in cell culture to test compounds for pro-migratory or anti-migratory activity. Depending on the migratory effect of the tested substances, the assay is performed over 2–4 days. ECs are grown to confluency in a cell culture dish and then scraped with a razor blade/pipette tip [41], allowing the EC at the wound edge to migrate into the scraped area. To really examine the motility contribution to the healing and to exclude the component related to cell proliferation, ECs are incubated with the antimitotic agent mitomycin [42]. Large genome-wide screens can also be assessed with the scratch wounding technique. The use of precision wounding replicators with floating pins and a workstation robot enables large numbers of scratches to be made with reduced coefficients of variation [43, 44].

Wound healing assay connected with video-lapse microscopy allows studying in 2D dimension the role of collective migration in angiogenesis and vascular development [34–36]. The use of aortic rings (see below) and that of specific microfluidic devices represent a further tool to describe this process in a 3D architecture [45]. For instance, wound healing assay exploited by single-cell analysis and by using chimeric EC sheets obtained by infecting cells with different fluorescent proteins [34, 45, 46] was instrumental to describe the following steps of EC collective migration: (1) In resting state, ECs undergo random cell motility in the monolayer with a regulated dynamics of homotypic cell junctions; (2) the presence of cell-free space (i.e., the wound) and a chemotactic gradient results in the appearance at the sheet margin of leader cells, which is characterized by an aggressive phenotype with prominent stress fibers, ruffling lamellipodia and enlarged focal adhesions, formation of peripheral actin cables, and discontinuous adherens junctions, which indicate mechanical coupling between leader and follower cells in the migrating cluster [47]; (3) as leaders start to migrate in the free space, a follower phenotype appears within cells of the monolayer.

#### Trans-well cell migration assay—Boyden chamber assay

The Boyden chamber assay is a useful tool to study chemotaxis and cell invasion. It was originally introduced by Steven Boyden in the 1960s for the analysis of leukocyte chemotaxis [48], and a modified version of the assay has recently been used extensively for the assessment of monocyte migration [39]. Indeed, today, a large range of Boyden chamber devices, adapted to individual needs, are commercially available. The assay is based on a chamber of two medium-filled compartments separated by a microporous membrane of defined pore size and can be used to distinguish positive chemotaxis (migration toward the attractant) and negative (migration away from a repellent) chemotaxis. Briefly, ECs are placed in the upper compartment and are allowed to migrate through the pores of the membrane into the lower compartment. The chemotactic agent of interest or cells-secreting chemotactic agents are present in the lower compartment. The membrane between the fluid-filled compartments is harvested, fixed, and stained after a defined incubation time, and the number of cells that have migrated to the bottom side of the membrane is determined by staining and subsequent microscopic analysis. Because a chemical gradient cannot be maintained for extended periods, Boyden chamber assays are limited to 2–6 h.

Boyden chamber assays are also utilized to measure different types of chemotaxis, including haptotaxis, transmigration, and cell invasion. Angiogenesis and transendothelial migration are special forms of haptotaxis, as the trigger for migration requires not only a chemokine, but also the presence of cell surface or extracellular matrix (ECM) molecules. In this case, the insert on the bottom of the Boyden chamber is coated with purified cell surface ligands (e.g., ICAM1, VCAM1) or ECM proteins (e.g., collagens, fibronectin), evaluating the migration of cells exposed to specific adhesion sites. Transmigration describes the migration of cells, such as leukocytes or tumor cells, through the vascular endothelium and toward a chemoattractant. Therefore, the assay measures transmigration of cells through a confluent, tight EC layer. Angiogenesis requires the invasion of EC through the basement membrane to form sprouting capillaries. Invasion processes can also be modeled in a Boyden chamber assay by coating the well membrane with a layer of Matrigel or collagen. In this case, cells must secrete matrix metalloproteases to degrade matrix proteins and migrate (invade).

The bioactive molecules in Boyden chamber assays can be provided directly by adding recombinant proteins or small molecule compounds or by plating cells-secreting specific factors in the bottom chamber. Manipulation of test cells (gain-of-function/loss-of-function) can be introduced into the assay. Migration assays are often performed in co-culture with tumor cells, pericytes or fibroblasts.

#### The real-time random migration assay

The use of video-lapse microscopy allows measuring migration not only as an endpoint result, but gives information on single-cell parameters, on morphological changes, and on the influence exerted by specific substratum. Subconfluent ECs are plated on plastic surface coated with specific extracellular matrix proteins (i.e., collagens, fibronectin, vitronectin) allowed to adhere and then observed with an inverted microscope equipped with thermostatic and CO_2_-controlled chamber (e.g., Leica, DMi8 platform; Nikon, TE microscope). Images of motile ECs are captured with a 5 min time interval over 4 h. Images were then processed with DIAS software (Solltech). A recent review on tracking algorithms offers a wide and comprehensive selection of the available tools to analyze cell motility [49]. Generally, data are displayed as a centroid plot showing the location of the geometrical center of the cell as a function of time. Directional persistence was calculated by determining the ratio between the net path length and the total path length. Furthermore, other parameters such as the total and net distance, the speed, the feature of turning angle can be calculated. Single-cell trajectories were plotted using MATLAB software and displayed in windrose graphs [50, 51].

### 2.2 Limitations and challenges

Standardization of techniques is one of the most critical issues to ensure the reproducibility of experimental results, and one has to be aware that cellular in vitro systems represent only a surrogate of the in vivo conditions. Nevertheless, compared to in vivo experiments, in vitro assays are relatively simple to perform and they offer the possibility to pursue high-throughput screens of compounds or supernatants of tumor cells affecting EC migration, e.g., supernatants of tumor cells. Nonetheless, the assay has limitations. The cell culture conditions must be standardized, and pure populations of EC are required. More frequently than not, human umbilical vein ECs (HUVECs) are used for these assays; however, these are derived from a large vessel, whereas angiogenesis occurs in microvessels. HUVECs are primary cells and are only viable for a limited time, and like other cells in culture, they change their expression profile and therefore their phenotype and behavior over time and through repeated passage events. Furthermore, reproducibility of scratch assays relies strongly on the initial degree of confluency [52]. In addition, scratch assays must be carefully evaluated to rule out the possibility that experimental conditions have influenced proliferation of EC, rather than their migration (this can be easily done using cell proliferation assays such as expression of pHH3, see below). Another important consideration is that ECs in vivo are exposed to shear stress as well as hypoxia gradients, i.e., the drag force induced by blood flow to the endothelium, which are absent in the standard static cell culture models. While the scratch assay is a straightforward cell culture assay to analyze EC chemokinesis, it does not have a high degree of sensitivity, but it is a useful tool to perform large-scale screening experiments. One downside of this method is that the width of the scratch introduced into the cell layer is difficult to control and cannot be easily standardized. Moreover, wounding of the monolayer with a sharp object may scratch the surface of the cell culture dish and additionally damage the EC at the migration front.

To obtain more reliable and reproducible results, fencing techniques that allow for controlled release of a confluent monolayer have been developed. These enable lateral migration without wounding of the cells or the underlying matrix [53]. Cells are grown as a monolayer in a culture dish containing a silicon template of defined size prior to seeding the cells. Once the culture reaches confluency, the silicon template is removed, thus allowing the cells to migrate laterally into the area previously occupied by the silicon template. Precise microscopic quantification of lateral migration is then possible over 2–4 days. Ideally, the duration of the assays should be kept under 2 days, because over longer incubation times, cell proliferation will contribute to wound closure, thereby confounding the migration effect.

As previously mentioned, a potential drawback is the difficulty of standardizing the wound areas, but this can be overcome using silicon templates [53]. Likewise, several commercial suppliers have developed robust assays that also circumvent this problem. For example, Essen Biosciences marketed a mechanical pin tool, the WoundMaker™, which applies equal pressure to create 96 homogeneous 700–800 μm scratches in each microplate well (www.essenbioscience.com/en/products/incucyte). Wound healing is visualized in real time using the IncuCyte ZOOM™, and cell tracing is fully automated, thus facilitating analysis and reproducibility. A similar device, providing automated hardware and analysis software, is provided by Peira Scientific Instruments (Beerse, Belgium) [54]. Electric cell-substrate impedance sensing (ECIS, Applied Biophysics Inc, New York, USA) can also be used to automatically create wounds and simultaneously measure cell migration in multiple wells, and V&P Scientific has developed a wounding replicator using floating pins that deliver a precise scratch (www.vp-scientific.com/wounding_tissue_culture_experiments.php) by a Sciclone ALH 3000 workstation robot (Caliper Life Sciences, Hopkinton, MA). The additional precision of the scratches using the robot over manual wounding replicators makes the latter instrument the method of choice when performing the scratch wound assay for large-scale screens on drug or genome-wide siRNA/sgRNA screens [43, 44].

It should be stressed that the Boyden chamber assay is somewhat delicate and it requires experience in handling. The most critical issue is the possible trapping of air bubbles in the lower and upper chambers during assembly. Air bubbles appear as empty spaces on the filter at the conclusion of the assay, because they hinder cell migration. For manual analysis, this may not be of major importance, but it becomes relevant if an automated analysis is performed since trapped cells would be undistinguishable from the absence of migration. It is important to invest substantial time into the setup and troubleshooting of the assay in order to yield robust and reliable results. It is also recommended to include a checkerboard analysis to distinguish between chemotaxis and chemokinesis effects. To this end, different dilutions of the compound to be tested should be titrated in the upper and lower chamber. Equal concentrations in the upper and lower chamber should lead to the same migration behavior as in the control for a compound that strictly responds to a gradient (i.e., chemotaxis).

### 2.3 Concluding remarks

In summary, the lateral scratch wound assay [55] and the Boyden chamber assay are both robust and reliable platforms to study EC migration. They are suitable for scaled-up purposes in order to perform manual or automated large-scale compound screens. Various vendors provide scratch and transwell assay systems. Although these systems offer good reproducibility and adequate throughput capacity, variation between individual EC isolations can occur resulting in variable results. Pooling several EC isolates may reduce this variability. In combination with time-lapse microscopy, it is also a powerful tool for tracing the migratory behavior of individual EC.

## 3 Endothelial cell proliferation assays

Many regulators of angiogenesis have been identified, validated, and developed based on their effects on EC proliferation. ECs are among the most quiescent cells in the body, with proliferation rates approaching zero under steady-state conditions. Only after stimulation, usually as a consequence of injury, inflammation, or pathological processes such as malignant growth, can they initiate cell cycle entry [56, 57]. The ideal assay to measure EC proliferation should be rapid, reproducible, and reliable and wherever possible should exclude inter-operator variability, for example through quantitative computational readout rather than qualitative researcher-dependent observations [57]. This section presents different methods and elaborates on problems and pitfalls.

### 3.1 Types of proliferation assays

**Fig. 1:**
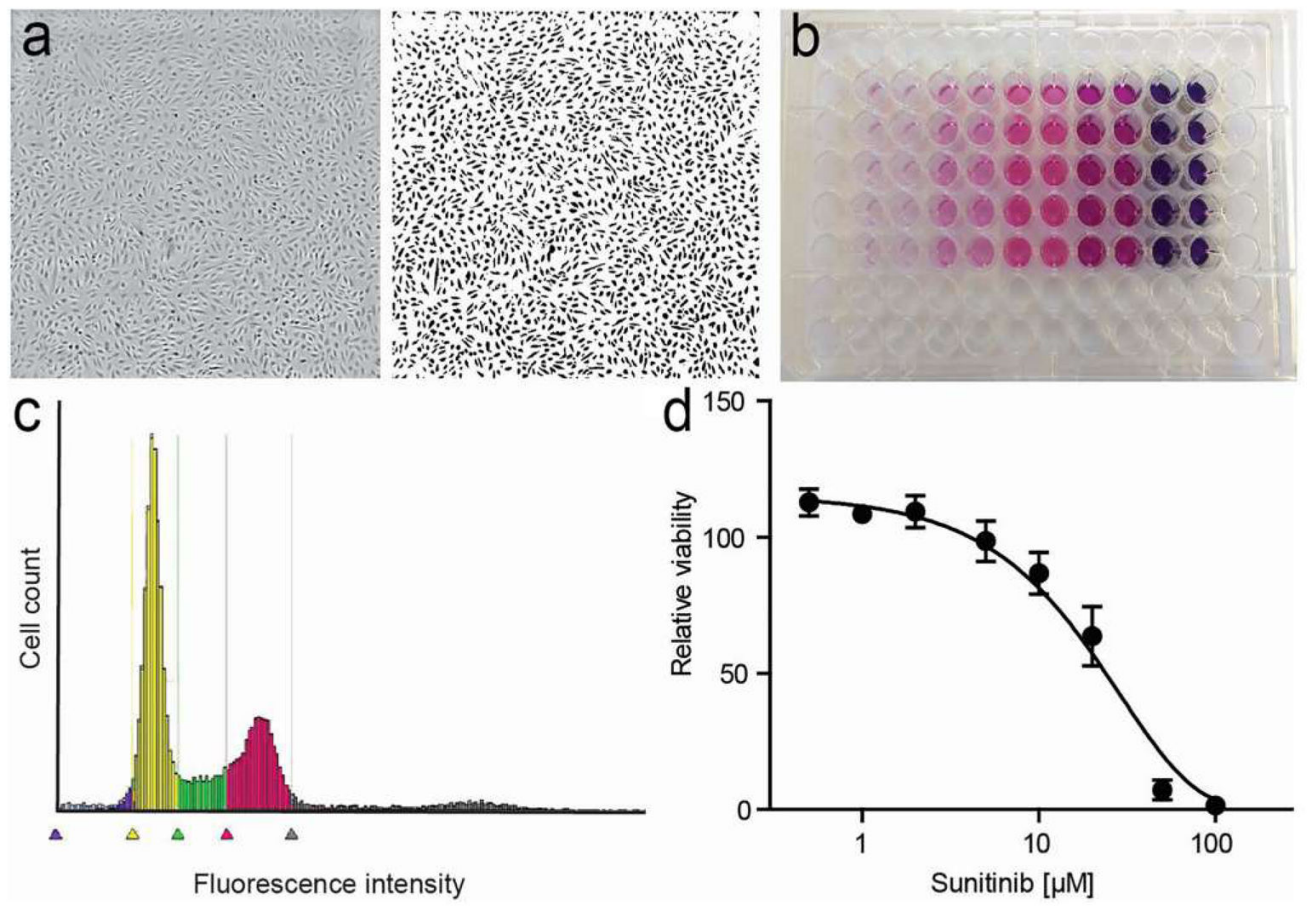
Endothelial cell proliferation assays. **a** Phase-contrast image (left) and binarized image of HUVEC grown in a regular 96-well plate. Simple software solutions can be used to count features in the image. **b** Example of MTT assay, with color intensity correlating with cell number. **c** DNA staining profile of HUVEC using PI, measured on a plate cytometer. **d** Cell viability of HUVEC exposed to sunitinib, measured using a luminescent assay

A number of different approaches to address cell proliferation have been developed in the last decades. In general, these include assessment of cell number, detection of DNA synthesis by incorporation of labeled nucleotide analogs, measurement of DNA content, detection of proliferation markers and metabolic assays (Fig. 1). Depending on the broadness of the definition of cell proliferation, which can range from the narrow description, for example, “the fraction of cells dividing over time” to the more general “the doubling time of a population,” several different assays may be pursued. Apart from that, means and equipment available will also dictate the choice of a particular method. As all methods focus on a particular aspect of the process, it is highly recommended to verify results with a complementary assay.

#### Cell counting

Cell counting is considered the gold standard for proliferation. Moreover, at least in theory, it is one of the most straightforward procedures for measuring proliferation of a cell population. It can be done using automated cell counters (e.g., Beckman Coulter) or by using a hemocytometer after removal of the cells from the culture vessel [57, 58]. More recently, different automated platforms have entered the market that allow analysis of cells while present in microplates, such as plate cytometers, automated microscope, or high-content screening platforms, that are compatible with cell counting-like procedures. With these, cells can be monitored over time but frequently require staining for detection, calibration, and (computation-assisted) quantification by, for example, staining of nuclei. Moreover, real-time cell analysis (referred to as RTCA) platforms have emerged that allow label-free, automated, real-time monitoring of cellular properties during incubation based on electrical resistance measurements. The equipment, however, requires considerable investment, beyond reach for many laboratories.

#### DNA labeling

During S phase of the cell cycle, DNA is synthesized and subsequently divided between the daughter cells (2 N → 4 N → 2 N; N = number of a complete set of chromosomes). Addition of modified nucleotides to the culture medium will result in their incorporation into the newly synthesized DNA. Adhering to the narrow definition of proliferation as stated above, this type of assay most closely reflects a means of measuring the fraction of actively dividing cells. It should be noted that this technique does not directly measure cell division or population doublings, but exclusively incorporation of a tracer into DNA synthesis.

^3^H-thymidine has been used in proliferation assays for decades [19, 56, 58, 59]. Briefly, cells are pulsed with ^3^H-thymidine for several hours and radioactivity is measured by liquid scintillation counting. This provides a very accurate representation of DNA synthesis and it is highly sensitive since the amount of incorporated ^3^H-thymidine is directly proportional to the rate of DNA synthesis [57, 58]. Constraints on using radioactive compounds and the rise of alternative methods have limited its use. In a similar manner, the incorporation of 5-bromo-2′-deoxyuridine (BrdU) or EdU (5-ethynyl-2′deoxyuridine) can be measured. BrdU or EdU can be (in)directly detected and subsequently be (semi-) quantified using ELISA, flow cytometry, or immunohistochemistry [57, 58, 60]. The latter two quantification techniques allow one to determine the fraction of dividing cells. These uridine analogs can be combined with DNA dyes (see below) to gain additional cell cycle information [57].

Another approach is the measurement of cellular DNA content using intercalating dyes such as PI (propidium iodide) or DAPI (4′,6-diamidino-2-phenylindole). Using flow or plate cytometry, a profile of the distribution of cells over the different phases of the cell cycle can be visualized, represented by DNA contents of 2 N (G1/0), 4 N (G2/M), or mixed (S). In addition, this method allows for the detection of apoptotic cells that would exhibit a subG1/0 (< 2 N) DNA content.

An alternative method to study EC cycle is based on the use of Fucci (fluorescent, ubiquitination-based cell cycle indicator) technology (Thermo fisher). It consists of a fluorescent protein-based system that employs both a red and a green fluorescent protein, respectively, fused to cdt1 and geminin, which are two regulators of cell cycle. These two proteins are ubiquitinated by specific ubiquitin E3 ligases in a specific temporal sequence. In the G1 phase, geminin is degraded; therefore, only cdt1 is present and appears as red fluorescence within the nuclei. In the S, G2, and M phases, cdt1 is degraded and only geminin remains, resulting in cells with green fluorescent nuclei. During the G1/S transition, when cdt1 levels are decreasing and geminin levels increasing, both proteins are present, giving a nuclear yellow fluorescence. More recently, Fucci probe was reengineered to generate a triple color-distinct separation of G1, S, and G2 phases extending the use of this technology to quantitative analyze the interphase of cell cycle [61].

#### Proliferation markers

Cell division is a highly coordinated process in which specific proteins act in concert with allow progression through different stages of cell division. Detection of these proteins, usually through immunochemical procedures, allows the estimation of the fraction of dividing cells. This approach can be used in in vitro endpoint assays, but can additionally be used to evaluate active EC proliferation in tissue sections (see separate section). The most frequently used markers are phosphorylated histone H3 (PH3), which marks cells in S phase, or PCNA and KI67, which label cells in all cell cycle stages except G0.

#### Metabolic assays

Gradually, the use of cell viability assays has taken a dominant position in addressing cell proliferation [62]. While not reflecting this property in its narrowest sense, if properly conducted, these assays accurately represent the number of live cells. They are readily available and require minimal handling and infrastructure. The most well known is the MTT assay (3-(4,5-dimethylthiazol-2-yl)-2,5-diphenyltetrazolium bromide), in which this yellow salt is taken up by metabolically active cells and converted by mitochondrial dehydrogenase to insoluble purple formazan crystals. As the amount of the converting enzyme is highly stable in a given cell population, the generation of formazan (and hence color intensity) is proportional to the number of viable cells. This is subsequently quantified by solubilization of the crystal-containing cells and spectrophotometry [63]. Variations in this method, for example, involving less toxic reagents, simplified reaction steps, or alternative readouts such as cellular ATP levels, have also been widely used [54, 58, 64]. In particular, the water-soluble tetrazolium salts, such as MTS (3-(4,5-dimethylthiazol-2-yl)-5-(3-carboxymethoxyphenyl)-2-(4-sulfophenyl)-2H-tetrazolium), allowing the detection of the colored product directly in cell culture media without solubilization, facilitate high-throughput and pharmacological screenings [65].

### 3.2 Limitations and challenges

#### Endothelial cell culture considerations

Studying EC proliferation in vitro requires a purified population of EC compatible with the assay setup. As already mentioned before, HUVECs are a widely available source, but have limitations regarding phenotype and life span in vitro. Therefore, other sources of EC are necessary for confirmation of results. Foreskin-derived human dermal microvascular ECs (HDMECs) or human dermal blood microvascular ECs (HDBECs) are good alternatives; however, the user should be aware that they represent mixed populations of blood and lymphatic endothelial cells [66].

It is essential to standardize the protocol of cell culture and propagation, by adhering to a fixed scheme of passaging, in order to use passage number as a surrogate for population doublings [67]. In all cases, cell density needs to be carefully controlled. Assay linearity can be compromised when cells are plated at too high (e.g., 50,000 cells/cm^2^) or too low (e.g., 5000 cells/cm^2^) a density. Loss of cell–cell contact is a potent stimulus for EC to proliferate, whereas ECs enter a quiescent state upon confluency, a process known as contact inhibition [57, 58, 63]. Optimization of the dynamic performance should involve synchronization of cells by exposure to low serum conditions (when studying pro-angiogenic molecules), or by stimulation prior to the addition of angiostatic factors or anti-angiogenic drugs [6]. Though immortalized EC can pose a helpful alternative, it should be recognized that the immortalization itself will likely alter growth control and survival mechanisms in these cells [67]. As such, care must be taken to address the generalizability of assay outcome.

#### Assay choice considerations

Each type of EC proliferation assay described here has limitations. Though cell counting is the most straightforward method, it can be prone to sampling error when cell detachment is required. Furthermore, it can be labor-intensive and it requires relatively large samples [57, 58]. However, it can be executed in the absence of toxic, mutagenic, or radioactive compounds, such as metabolic, DNA labeling, and DNA incorporation-based assays [57, 58, 63]. Of note, the outcome of these assays is adversely affected by EC death. From a methodological point of view, each assay has its strengths and weaknesses. For example, the indirect detection of antigens (e.g., PCNA or BrdU) requires careful procedural optimization. Although the alternative “click” chemistry, by which the analogous EdU can be detected directly, circumvents this issue of indirect detection, making EdU preferable [57, 58, 60]. Nonetheless, assay readout and interpretation are important to consider. When measuring incorporation of nucleotide analogs, one should realize that DNA synthesis is not con-fined in all situations to chromosomal duplication during S phase [57, 58]. For example, during DNA repair, nucleotides are excised and replaced, which is especially relevant when addressing the action of compounds with a potential DNA damaging effect. With DNA-intercalating dyes, care must be taken that doublets are excluded in the gating procedure or with readout in plate-based systems. By nature, this type of assay is mostly suited for truly diploid cells, and not for cells that may display alternative karyotypes. Although the latter is not a common trait of EC, a few reports have addressed this matter in tumor-derived EC [68], and personal observations also indicate this may be the case with EC lines. Finally, test reagents may interfere with readout chemistry; thus, compounds that affect mitochondrial function are less compatible with metabolic assays. Surrogate assays such as the VEGFR-BaF3 cell lines which bind VEGF ligands and signal through chimeric receptors in reporter cell lines have been very useful (both academically and in industry) for quantifying the presence of major angiogenic factors from human, mouse, and viral sources that stimulate the VEGFR-2 and VEGFR-3 pathways [69, 70].

### 3.3 Concluding remarks

The choice for a particular EC proliferation assay is determined by a number of considerations. Endpoints, test compounds, laboratory infrastructure, scale, required throughput, convenience, and cost all influence the applicability of an assay system. The growth of a cell population is influenced by both cell division and death, which are difficult to monitor simultaneously. Most important is the researchers’ awareness of strengths and weaknesses of each individual assay. Importantly, interpretation of data must be done with care, and whenever possible, results are validated with an alternative method.

## 4 3D models of vascular morphogenesis

The emergence of vascular networks either through vasculogenesis or angiogenesis requires the association of cells into stable 3D tubes in a process that involves differentiation, migration, proliferation, aggregation, and rearrangement of these cells to form cords that then undergo lumen formation. Taken together, this process is referred to as vascular morphogenesis. Subsequently, ECs recruit perivascular stromal cells (pericytes) to stabilize this newly formed network and minimize leakage upon blood perfusion. Importantly, not all sprouts become functional vessels. Pruning serves to selectively remove redundant or non-functional vessels to optimize fluid flow through the network [71].

In vitro assays have played a valuable role in our understanding of vascular morphogenesis. These assays provide a simpler platform than animal models for dissecting individual steps within the process while also incorporating 3D matrix to mimic native in vivo tissues. Here, we present several of the most reliable and informative assays developed to date and highlight the strengths and limitations of each (Table 1). While many types of EC can be used in these assays, the most commonly used are HUVEC and human endothelial colony-forming cell-derived EC (ECFC-EC), which generally have a higher proliferative potential. Mouse ECs are not generally used in these assays as they are notoriously difficult to maintain in culture. While we use “EC” to reference to cells derived from both species, assays using a specific EC source are annotated accordingly.

### 4.1 Types of assays

#### Fibrin Bead Assay

**Fig. 2:**
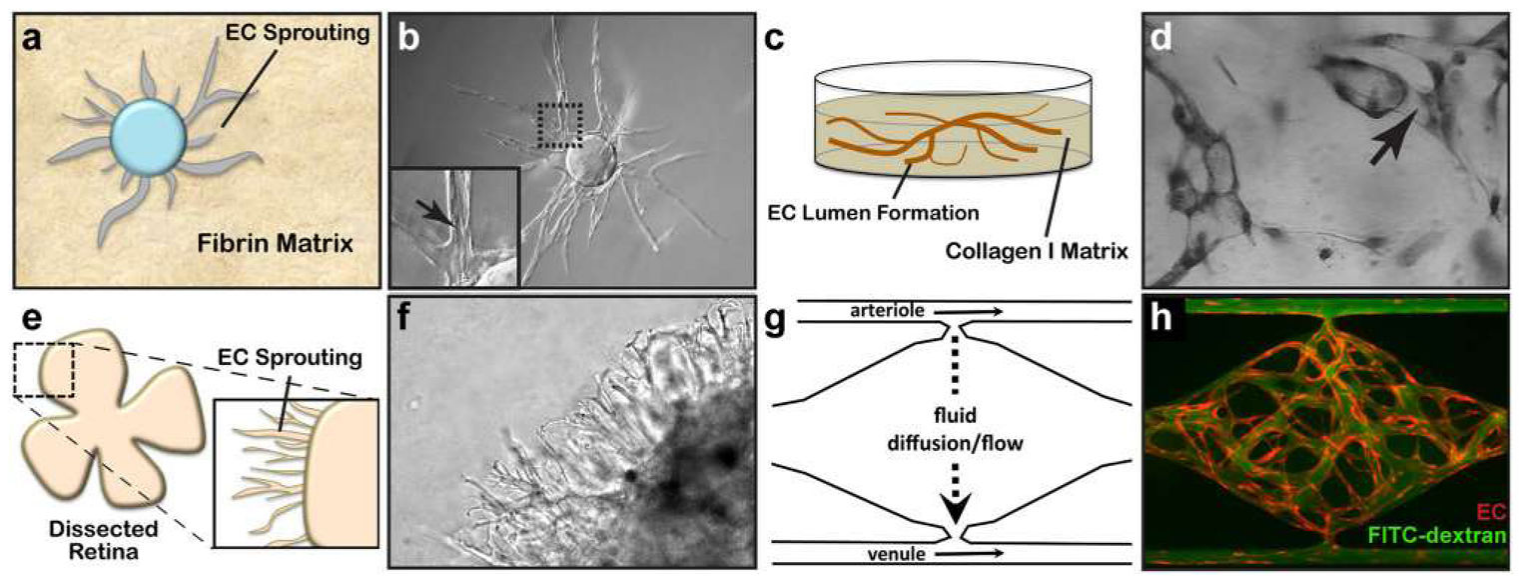
Three-dimensional assays of vascular morphogenesis. **a** A fibrin bead assay uses collagen I- and EC-coated Cytodex beads embedded within a 3D fibrin gel matrix to measure EC sprouting and lumen formation. **b** These features are readily resolved using phase-contrast microscopy. **c** EC tube formation can be measured by embedding EC within a collagen I matrix. **d** Once formed, these tubes can be visualized by toluidine blue staining and bright-field microscopy. **e** Whole-mount, dissected retinas from postnatal mice are mounted within collagen I-Matrigel matrix mix and cultured in pro-angiogenic medium to stimulate EC sprouting. **f** Sprout and lumen formation are resolved using phase-contrast microscopy. **g** The vascularized micro-organ (VMO) approach utilizes “arteriole” (high pressure) and “venule” (low pressure) microfluidic channels to drive medium diffusion and flow across a cell chamber where microvasculature forms. **h** The formed microvasculature (EC, red) can be measured for leak by perfusion with 70 kDa FITC-dextran (green)

Traditional Matrigel cord-forming or collagen I angiogenic invasion assays are insufficient to mimic the complexity of angiogenesis, as these assays are two-dimensional and ECs in these assays often form incomplete lumens. Moreover, lumen formation in Matrigel is not unique to EC as several non-EC cell types (e.g., human prostate carcinoma and glioblastoma cells) also form cords, complicating the interpretation of results from these assays [57]. By contrast, the fibrin bead assay provides a platform for testing EC sprouting and lumen formation over an extended period (2–3 weeks) and incorporates a 3D, ECM, and multiple cell types (i.e., stromal pericytes) to model native angiogenesis. ECs (HUVECs) are first allowed to adhere to collagen I-coated Cytodex beads to generate an EC monolayer that mimics the vessel wall of native vessels. These EC-coated beads are then embedded into a fibrin gel with human stromal cells either embedded within the gel or plated as monolayer on the surface of the gel. Tip cells are observed 2–3 days post-plating, and elongating sprouts appear 2–4 days thereafter (Fig. 2a, b). When maintained in pro-angiogenic EGM-2 medium (Lonza), lumens form within a week and the cells remain viable up to 3 weeks, at which point anastomoses between sprouts are often apparent [72]. A detailed, video protocol of this assay is available [73].

Angiogenic sprouting from individual beads is evaluated by phase-contrast microscopy allowing for quantification of sprout number, length of sprouts, percentage of sprout lumenization, and number of anastomoses. Genetic approaches (siRNA, lentiviral transduction, CRISPR/Cas9) [74] can modify gene expression in individual cell types to dissect cell-autonomous components of the angiogenesis process. Protein expression and localization are measured by fixing bead assays and using modified immunofluorescent staining techniques. More detailed gene expression analyses are made possible by harvesting individual cell types to track RNA expression changes over time through various stages of sprouting angiogenesis.

The use of stromal cells (e.g., lung fibroblasts) is critical to the success of this assay, as these cells secrete angiogenic factors necessary for EC sprouting and lumen formation, including growth factors [hepatocyte growth factor (HGF), transforming growth factor alpha (TGF-α), and angiopoietin-1 (Ang-1)], as well as matrix molecules, matrix-modifying proteins and matricellular proteins [e.g., procollagen C endopeptidase enhancer 1, secreted protein acidic and rich in cysteine (SPARC), transforming growth factor-β-induced protein ig-h3 [βIgH3], and insulin growth factor binding protein 7 (IGFBP7)]. These factors act to locally stiffen the matrix, which supports sprouting and lumen formation [75]. This assay represents a significant improvement over conventional, single-cell-type angiogenic assays, as the inclusion of multiple cell types more closely mimics the physiological environment. Nevertheless, as this assay uses primary cultures of cells, rather than cell lines, it is important to remember that batch-to-batch variations in stromal cells (and EC) can significantly affect assay results. To partially overcome these issues, it should be appropriate to use ECs pooled from 5 to 10 umbilical cords. Generally, it is crucial to identify stromal cell-HUVEC pairs that yield optimal angiogenic sprouts.

#### Collagen Lumen Assay

To investigate EC lumen formation mechanisms, early assays seeded EC in monolayers on plastic dishes coated with ECM proteins (i.e., collagen I, collagen III, fibrin, or Matrigel). While these 2D assays are sufficient to induce EC cord formation [76–78], they cannot reproduce the necessary cues for true lumen formation found in native, 3D tissues. Collagen sandwich assays surround EC within a 3D matrix by seeding the cells in monolayer on collagen I matrix and then covering them with a second layer of collagen [79]. Nevertheless, tube formation fails to occur in a random, 3D growth pattern, forming only in the *X*–*Y* plane of the initial gel layer and not in the z-axis. As this does not adequately recapitulate normal vessel growth in a true 3D environment, George Davis and others further optimized these assays, opting instead of embedding single EC (HUVEC) randomly throughout a collagen I matrix. In the simplest version of these assays, HUVECs are seeded at low density (7 × 10^5^ cells/ml) under serum-free growth conditions and with the addition of minimal growth factors (phorbol ester, VEGF), and fibroblast growth factor-2 (FGF-2). After 48 h, the embedded ECs form intact tubes throughout the gel, with clearly demarcated lumens (Fig. 2c, d). Several variations on this assay have since enhanced and optimized lumen formation. First, the addition of several other growth factors, including a cocktail of stem cell factor (SCF), IL-3, stromal-derived factor-1α (SDF-1α), and FGF-2, further promotes lumen formation while maintaining serum-free growth conditions. Second, when simultaneously seeded within the same matrix, stromal pericytes are recruited by EC, recapitulating a key step in vascular morphogenesis. Lastly, to understand the process of EC sprouting and angiogenesis, EC can be seeded on top of a 3D collagen gel containing the same growth factors and invasion of the underlying gel layer can be quantified. A detailed protocol of the collagen lumen assay and its variations is available for further reading [80].

Real-time imaging of tube formation can be achieved using fluorescent protein-transduced EC. Alternatively, fixed vessels can be stained with 0.1% toluidine blue and imaged using bright-field microscopy (Fig. 2d). More in-depth analyses can be carried out on these fixed vessels using immunofluorescence staining of relevant protein markers or transmission electron microscopy to resolve structural details of formed lumen and remodeled ECM.

Regular users of collagen gels will note that the viscosity, pH, and contraction of these gels can hinder successful execution of assays in the hands of new users. As a result, special care should be taken when pipetting (such as when mixing cells and growth factors) and plating gels to ensure even gel coating of the bottom of the well plate. Perhaps most significantly, early gel contraction can limit the useful length of these assays. Users will note that plating gels only in wells within the center of the 96 half-area wells and adding medium or water to the outer wells of the plate will minimize gel contraction, by maintaining local humidity levels. Additionally, seeding fewer ECs within the collagen (1.5 × 10^3^ cells/ml) can minimize gel contraction and prolong the assay.

#### Retinal Explant Assay

Although in vitro assays are high throughput and can mimic major steps in vascular morphogenesis, they do not fully recapitulate the in vivo, whole-organ environment [72]. Several in vivo animal models, such as mouse retina or zebrafish fins, are valuable tools for studying vascular (re)-establishment in a physiologically relevant context [57, 81]. However, the added complexity of these systems makes it more difficult to ascertain the role of individual proteins and growth factors and cell types in the vascular morphogenesis process, relying on genetic manipulations or system-wide administration of pharmacologic inhibitors to dissect molecular pathways [57, 82]. As such, there is a need to increase assay complexity and physiological relevance while developing platforms amenable to ex vivo study in the laboratory. Retina explant assays are one such ex vivo platform, whereby dissected retinas are maintained and observed for vascular morphogenesis over several weeks in the laboratory. While multiple versions of this assay have been published, a protocol published by Sawamiphak et al. is most widely used for the study of endothelial sprouting [83]. Briefly, retina cups from embryonic, postnatal, or adult mice are harvested and cut radially to allow flat mounting of the retina interior surface onto a membrane insert. After recovery in media for 2–4 h, the explants can then be treated with stimulatory or inhibitory agents for up to 4 h, followed by whole-mount microscopy analysis to evaluate the (anti-) angiogenic effect of these agents on vessel sprouting (Fig. 2e). A trained researcher can harvest and dissect each pair of retinas within minutes. Unfortunately, without the support of a 3D matrix, retinal cells cannot survive for long periods, thus making studies of later stages of angiogenesis impossible. To overcome this, Rezzola et al. have improved the assay by embedding the retinas in different matrices after dissection [84]. In this approach, retinas can be crosscut into four equal pieces and left in serum-free media overnight. The retina fragments are then embedded in Matrigel, collagen I, or fibrin matrix and fed every 2–3 days. Depending on the age of the mice and the matrix used, sprouts can be observed between days 3 and 6 and anastomosis of neighboring sprouts similar to what occurs in vivo can be observed in 10–14 days [85] (Fig. 2f). These explants can be maintained up to 3 weeks before the vessels eventually regress. When cultured for a longer period however, vascular sprouts also start to form toward the chorioidea and not only in the retinal plane, making analysis more complex.

Vessel formation can be analyzed in real time using time-lapse imaging or immunofluorescence microscopy of fixed explants at established experimental time points [85, 86]. Gene expression can be manipulated by genetic crossing of the donor mice or, more transiently, by treating retinas with lentivirus or siRNA or Crispr-Casp9 technology. Moreover, embedded retinas can be treated with drugs over extended periods to dissect individual signaling pathways.

Several factors are critical to consistently achieve sprouting from dissected retina explants. Firstly, the matrix proteins in which retinas are embedded can greatly influence how vessels sprout. Matrigel is far superior to single matrix proteins in inducing sprouting. However, the addition of 10–20% Matrigel in collagen I matrix is sufficient to stimulate sprouting compared to pure collagen I matrix. Secondly, the use of pro-angiogenic EGM-2 medium yields more sprouts as compared to basal medium alone. Lastly, as with any tissue explant, the age of the mouse can influence the degree of vessel sprouting. As such, special care should be taken to select mice appropriate for the experimental question at hand. There are many similarities between the mouse retinal explant assay and the traditional mouse/rat aortic ring angiogenesis assay or rat vena cava explant assay [87–89]. However, retinal explant models more closely model true capillary sprouting as the vasculature in these explants is actively developing and remodeling. This makes the retinal explant model uniquely suited to studying microvessel formation and its underlying mechanisms.

#### Vascularized Micro-Organ Platform

To understand all the steps of vascular morphogenesis in a single platform, a vascularized micro-organ (VMO) approach has been developed to drive formation of a perfusable vascular network within a 3D hydrogel matrix environment. In contrast to the assays described above, VMO-embedded ECs are exposed to and respond to shear stress, form lumenized vessels, and are perfused with a blood-substitute medium that delivers nutrients to tissues within the matrix, just as in the body. Specifically, this platform utilizes “arteriole” (high pressure) and “venule” (low pressure) microfluidic channels that are joined by a living microvascular network that forms by vasculogenesis in an intervening tissue chamber (Fig. 2g). A pressure differential between the two channels is used to drive interstitial flow through the fibrin gel matrix during vessel development, induce vessel formation (through shear-sensing), and drive convective flow through the mature vasculature once formed and anastomosed to the outer channels. This pressure difference is induced by varying the level of medium within fluid reservoirs at either end of the microfluidic channels, thereby creating hydrostatic pressure heads that ensure continuous fluid convection across the cell chamber. To form vessels, human ECFC-EC and human lung stromal cells are co-loaded within a fibrin matrix into the central cell chamber through an independent loading tunnel. When maintained in pro-angiogenic EGM-2 medium, vessels form within 4–6 days post-loading (Fig. 2h). When perfused with 70 kDa rhodamine dextran, a molecule similar in size to albumin, these vessels demonstrate minimal vessel leakage—comparable to in vivo microvasculature. For readers interested in more information, a detailed protocol for loading and maintaining the VMO platform is available [90].

The VMO platform is fabricated from polydimethylsiloxane (PDMS), an optically clear, biologically inert polymer widely used in the microfluidics field [91]. The use of this polymer and the dimensions of the platform ensure that live, GFP-transduced endothelial progenitor cells (ECFC-EC) can be imaged and quantified throughout vessel formation. Specific parameters such as vessel network length, branching, and anastomosis can be measured in real time as can vessel permeability by perfusion with fluorophore-tagged dextran molecules of various molecular weights. Additionally, immunofluorescent staining can be used to quantify expression of specific molecular markers or RNA can be collected to measure changes in gene expression. Lastly, gene expression can be manipulated by treating individual cell types with lentivirus or siRNA prior to loading in the platform.

To ensure robust and reproducible vascular network formation, several steps are critical. First, the fibrin gel matrix must be consistently loaded within the VMO cell chamber. During normal loading, perfusion burst valves at the interface between the tissue chamber and the microfluidic channels ensure a gel/air interface (later a gel/fluid interface) is formed. To simplify loading and minimize specialized training for new users of the platform, current iterations of the VMO platform incorporate a pressure release valve at the loading tunnel that minimizes unintended gel bursting [92]. Second, robust vascular network formation requires that vessels within the chamber anastomose with the outer microfluidic channels. To facilitate the formation of these anastomoses, EC can be either seeded directly within the microfluidic channels or induced to migrate from the gel by coating the external channels with ECM [93]. As with the fibrin bead assay, optimal stromal cell–EC pairs should be validated to ensure assay reproducibility.

### 4.2 Limitations and challenges

With all EC assays, the source of EC is critical to assay success. Although commercial versions of HUVEC and ECFC-EC are available, cells from these sources show limited utility in many 3D assays, likely due to a larger than optimal number of cell doublings prior to shipment and use in the laboratory. As a result, the use of freshly isolated EC will provide the most consistent results and is strongly encouraged. Readers will note that isolation protocols for both cell types are available [94, 95]. Additionally, patient-to-patient variation between different EC isolations can lead to inconsistent assay results, an issue that may be avoided by pooling several EC lines prior to use.

Given the many differences between the assays described here, the useful length of these assays varies considerably. Even in well-trained hands, the contraction of gels in the collagen lumen assay effectively limits the useful time frame of assays to 72 h or less. However, the other assays described here can continue for much longer periods of time, with the fibrin bead assay, retinal explant assay, and VMO platform all suitable for time points up to 3 weeks under appropriate conditions.

Lastly, the majority of these assays can be run in a relatively high-throughput manner, thereby accelerating the speed with which genetic, molecular, or pharmacologic screens [96] can be conducted to understand vascular morphogenesis. This is especially true with the fibrin bead and collagen lumen assays, which utilize multiple beads or multi-well culture plates to increase assay throughput. Similarly, while initial versions of the VMO platform were cumbersome to load in high-throughput numbers, this platform is now used in an optimized configuration that incorporates up to 16 individual VMO devices within a standard 96-well plate [97]. This design simplifies translation to outside laboratories and interfacing with existing microscope and plate reader infrastructure. Of all the assays described in this section, retinal explants are most adversely affected by delays between initial dissection, mounting, and plating of tissue samples. This inherently limits the number of animals that can be dissected at once and, for now, limits the number of retinas that can be screened simultaneously.

### 4.3 Concluding remarks

Vascular morphogenesis requires the interaction between several cell types and their surrounding, 3D microenvironment. The 3D vascular morphogenesis models described here provide unique in vitro culture systems that recapitulate this complex multi-step process. At the same time, the simplicity and consistency of these assays compared to in vivo models allow them to be conducted in a high-throughput fashion. Finally, the use of human cells in these assays improves the clinical relevance of such models, making them readily amenable to drug discovery applications.

## 5 Aortic ring assay

Explants of rat or mouse aorta have the capacity to sprout and form branching microvessels ex vivo when embedded in gels of ECM. Angiogenesis in this system is driven by endogenous growth factors released by the aorta and its outgrowth in response to the injury of the dissection procedure. This property of the aortic wall was first described in the early 1980s [98] and led to the development of the aortic ring assay [99], which is now widely used to study basic mechanisms of angiogenesis and test the efficacy of pro-angiogenic or anti-angiogenic compounds [100].

### 5.1 Benefits and strengths of the aortic ring assay

**Fig. 3:**
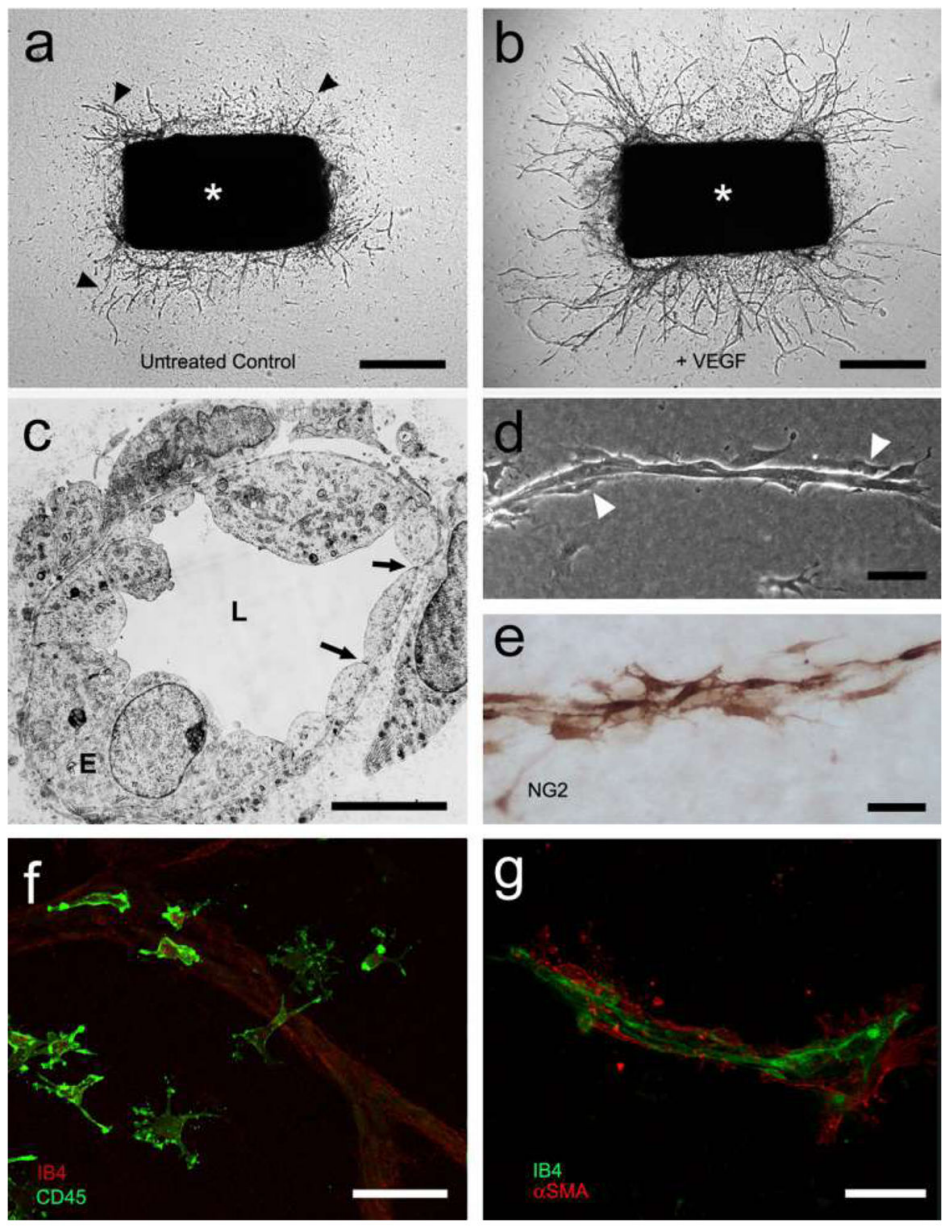
Aortic ring assay of angiogenesis. **a** Serum-free collagen gel culture of rat aorta (asterisk) photographed at day 6 (microvessels marked by arrowheads). **b** Aortic culture treated with VEGF (5 ng/ml) shows increased number of microvessels (day 6). **c** Electron micrograph of aorta-derived microvessel with polarized endothelium (E), patent lumen (L), and surrounding pericytes (P); endothelial tight junctions are marked by arrows. **d** Phase-contrast micrograph of microvessel composed of an inner core of endothelial cells and surrounding pericytes (white arrowheads). **e** Pericytes highlighted with immunoperoxidase stain for NG2. **f** Immunofluorescent image of aorta-derived macrophages stained for CD45; an isolectin B4 (IB4)-stained endothelial sprout is visible in the background. **g** Confocal image of microvessel double stained for endothelial cells (IB4) and pericytes (alpha smooth muscle actin, αSMA). Magnification bars = 500 μm (**a**, **b**), 5 μm (**c**), 50 μm (**d**–**g**)

The aortic ring assay offers many advantages over existing models of angiogenesis. Unlike isolated EC, the native endothelium of the aortic explants has not been modified by repeated passages in culture and retains its original properties. The angiogenic response can be inhibited or stimulated with angiogenic regulators and analyzed by molecular or immunochemical methods without the confounding effects of serum (Fig. 3a, b). Angiogenic sprouting occurs in the presence of pericytes, macrophages, and fibroblasts, as seen during wound healing in vivo [100]. The ultrastructure of neovessels at different stages of development can be evaluated by electron microscopy (Fig. 3c). The different cell types can be identified with specific cell markers by immunostaining whole-mount preparations [101] of the aortic cultures (Fig. 3d–g). Many assays can be prepared from the thoracic aorta of a single animal (approx. 20–25 cultures/rat aorta; approx. 10–15 cultures/mouse aorta). The angiogenic response can be quantitated over time, generating curves of microvascular growth. Aortic cultures can be used to study mechanisms of vascular regression, which typically follows the aortic angiogenic response as seen during reactive angiogenesis in vivo. Aortic rings transduced with viral constructs or obtained from genetically modified mice can be used to study the role of specific gene products in the regulation of the angiogenic response [100].

Recently, rat aortic ring assay was adapted to human arteries by using matrigel as 3D hydrogel [102].

### 5.2 Assay overview

A detailed description of the aortic ring assay protocol is available in previous reports [103, 104]. We provide here a summary of key steps for the preparation of the assay. Aortic rings are prepared from the thoracic aorta of 1–2-month-old rats or mice. After excision from the animal, the aorta is transferred to a Felsen dish containing serum-free Endothelial Basal Medium (EBM). Under a dissecting microscope, the aorta is cleaned of blood and fibroadipose tissue using Noyes scissors and microdissection forceps. Care is taken not to stretch, cut, or crush the aortic wall during the isolation and dissection procedures. As the dissection progresses, the aorta is rinsed in the four compartments of the Felsen dish. Using a scalpel blade, the aortic tube is then cross-sectioned into 0.5–1-mm-long rings. The most proximal and distal rings which may have been damaged during the dissection procedure are discarded. The remaining rings are washed through sequential transfers into eight consecutive baths of serum-free medium, using compartmentalized Felsen dishes. Aortic rings are then embedded individually into thin collagen, fibrin, or basement membrane gels as described. Once the gel has set, 500 μl of serum-free EBM is added to each culture. Each experimental group comprises quadruplicate cultures in four-well NUNC dishes. Aortic ring cultures are incubated in a humidified CO_2_ incubator at 37 °C for 7–21 days.

### 5.3 Quantitative analysis of angiogenesis in aortic cultures

The angiogenic response of the rat (or mouse) aorta can be quantitated by visual counts or by computer-assisted imaging. For visual counts, cultures are examined every 2–3 days and scored for angiogenic sprouting by using an inverted microscope with bright-field optics equipped with 4× to 10× objectives and a 10× eyepiece. Angiogenesis is scored by counting microvessel sprouts, branches, and loops according to previously published criteria [99]. Aortic outgrowths can also be quantified by image analysis using low power images of the cultures thresholded to highlight the vascular outgrowths [105–108]. Standard statistical methods are used to analyze data and determine levels of significance between control and treated cultures. An internal control group with untreated aortic rings must be included in each experiment to mitigate the effect of possible interassay variability.

### 5.4 Critical points

For this assay, we recommend using the thoracic aorta because of its uniform size and intercostal artery branching pattern. The abdominal artery can also be used, but its variable pattern of collaterals and tapering lumen may introduce variability in the angiogenic response. Injury to the aortic endothelium may be an additional cause of uneven sprouting from different rings. Therefore, special care must be taken not to damage the aorta by stretching or letting it dry during the isolation and dissection procedures. Dissection of the aorta and preparation of the aortic ring cultures are best performed in a tissue culture room with HEPA-filtered air to avoid microbial contamination. Best results with this assay are obtained using interstitial collagen or fibrin gels. Collagen can be produced in-house, as described [103, 104], or purchased from commercial sources [109]. Fibrinogen and thrombin for the fibrin gel are commercially available. Matrigel, a basement membrane-like matrix of tumor origin, can also be used [110]. Matrigel cultures, however, require growth factor supplements due to the limited ability of the aortic rings to sprout spontaneously in this dense matrix. The growth medium used for the assay should be optimized for the growth of EC in the absence of serum. Optimal results in collagen and fibrin cultures can be obtained with EBM. When preparing individual collagen gel cultures, given the small volume of gel (20–30 μl), it is important to remove excess growth medium from the aortic rings when they are transferred into the collagen, fibrinogen, or Matrigel solution. This is accomplished by gently streaking the aortic ring onto the bottom of the culture dish while holding it from the adventitial side with microdissection forceps. When working with fibrin gels, which set rapidly, no more than four cultures at a time should be prepared, to avoid disrupting the developing gel while positioning each ring. In addition, for fibrin cultures, the culture medium should include a plasmin inhibitor such as epsilon aminocaproic acid to inhibit fibrinolysis by the aortic rings, which would rapidly destroy the matrix needed for EC to sprout.

The rat aortic ring assay is robust and very reproducible when performed by an experienced operator. The mouse aortic ring assay is more variable than the rat aortic ring assay, likely because of the small size of the rings. For this reason, at least twice as many aortic rings should be used for this assay. Miniaturization of the assay using IBIDI microchambers [104] and a smaller volume of growth medium (50 μl) are recommended for the mouse aortic ring assay to ensure spontaneous sprouting under serum-free conditions [104]. In all cases, experiments should be repeated 2–3 times to obtain sufficient number of data points for statistical analysis. The growth medium can be replaced on a regular basis (3 times/week) or left unchanged for the duration of the experiment. If the medium is not replaced with fresh medium, the angiogenesis response and the stability of neovessels are enhanced due to accumulation of endogenous growth factors in the system. For immunohistochemical evaluation of the aortic cultures, biomatrix gels should not exceed 20–30 μl and should be well spread as a thin wafer around each ring. Formalin fixation should be limited to 10 min to avoid excessive cross-linking of proteins. In addition, overnight incubation may be needed for optimal penetration of the primary antibody into the gel.

### 5.5 Limitations and challenges

The main limitation of the aortic ring assay is the lack of blood flow, particularly for angiogenesis-related genes that are regulated by mechanochemical mechanisms. An additional potential limitation is the source of angiogenic ECs, which are arterial and not venous, as neovessels in vivo primarily sprout from postcapillary venules. Many studies performed with this assay, however, have shown good correlation of results obtained with the aortic ring assay and in vivo models of angiogenesis. If needed, the aortic ring assay methodology can be applied to veins as reported [89]. Some investigators have described variability of the angiogenic response in different aortic cultures. This is due to the delicate nature of the endothelium, which can be damaged because of inadequate handling of the aorta or the aortic rings, drying of the explants, or their excessive exposure to alkaline pH. Suboptimal preparation of the gels resulting in a defective matrix scaffold can also result in a poor angiogenic response. In addition, the age and genetic background of the animal significantly affect the capacity of the aortic rings to sprout spontaneously or in response to angiogenic factors. Aortic outgrowths in Matrigel are much denser than in collagen and fibrin and more difficult to quantitate by visual counts due to the intricate branching pattern of the endothelial sprouts and the tendency of mesenchymal cells to arrange in confounding networks, which mimic angiogenic sprouts. Immunostaining of the aortic outgrowths with endothelial markers followed by image analysis may overcome this limitation. For quantitative analysis of the angiogenic response, the visual count method (described in detail in Ref. [104]), becomes challenging when cultures stimulated by growth factors produce 250–300 or more vessels. Since the outgrowths of rings oriented with the luminal axis parallel to the bottom of the culture dish (recommended orientation) are typically symmetrical, angiogenesis in these cases can be quantified by counting the number of microvessels in half of each culture and then doubling the score. Alternatively, these cultures can be measured by image analysis [105–108]. Finally, for the whole-mount immunohistochemical stain, gel thinness is critical for optimal antibody penetration.

### 5.6 Concluding Remarks

Many of the molecular mechanisms orchestrating angiogenesis have been discovered, but many others remain to be identified, studied, and evaluated as targets for the development of new therapies. The aortic ring assay reproduces ex vivo cellular and molecular mechanisms that are essential for the regulation of the angiogenic process. As such, this assay provides an invaluable platform to test new hypotheses and analyze the efficacy of the next generation of angiogenesis-targeting drugs.

## 6 Tumor microvessel density and histopathological growth patterns in tumors

Microvessel density (MVD) is often regarded as a surrogate marker of angiogenesis in tumors. Angiogenic tumors contain areas with a high concentration of new but inefficient blood vessels, which have sprouted from existing vessels and are often arranged in a tortuous, glomeruloid tangle. In addition, to these “hot spots,” angiogenic tumors also contain areas of low blood vessel numbers. Weidner developed a method to assess MVD in vascular hot spots using pan-endothelial immunohistochemical (IHC) labeling assays, using lectins such as WGA, Ulex Europaeus, or antibodies against CD31, CD34, and von Willebrand factor and less often VE-cadherin, αvβ3 integrin, CD105, or type IV collagen [111]. Vascular hot spots are identified by light microscopy at low power magnification by scanning the entire stained tumor section. Individual microvessels are then counted at high power magnification in these regions. As an alternative, microvessels can be counted in regions defined by systematic uniform random sampling (SURS) [112]. These IHC staining assays are robust, fast, and easy to perform and to automate. Although results in individual studies are highly variable, meta-analyses have confirmed the prognostic value of MVD, for example in breast cancer [113] and isolated reports support the predictive value of MVD for anti-VEGF treatment [114, 115]. However, the inter-observer variability of the vessel counting algorithm presents a significant barrier to the use of MVD as a bio-marker [116]. Inter-observer variability can be reduced by applying strict counting rules and by consensus training of individual observers [112].

Although the importance of angiogenesis to tumor biology is well-established, multiple observations that primary and metastatic tumors develop and progress in the absence of angiogenesis suggest that other mechanisms are frequently involved in tumor vascularization [117]. This represents a challenge to Folkman’s hypothesis that the growth of a tumor is only possible when accompanied by angiogenesis [118]. Moreover, MVD cannot distinguish angiogenic and non-angiogenic tumors. In addition to the high numbers of blood vessels in angiogenic tumor hot spots, high MVD is indeed also observed in non-angiogenic tumors which have co-opted vessels in organs with extensive microvasculature, such as the liver and the lungs [117]. The differentiation of angiogenic and non-angiogenic tumors is, therefore, not related to the absolute number of vessels per surface area but to the growth pattern of a tumor.

### 6.1 Histopathological growth patterns

Histopathological growth patterns (HGPs) are defined according to the morphological characteristics of the tumor at the interface with the surrounding normal tissue. HGPs are identified by light microscopy in standard hematoxylin-and-eosin stained tissue sections, and distinct HGPs have been described for tumors that grow in the lung, liver, skin, brain, and lymph node [117]. Recently, international consensus guidelines for scoring these HGPs have been described [119, 120]. One of the important differences in the biology of tumors with specific HGPs is their means of vascularization. Liver metastases may present with one of two common HGPs, replacement or desmoplastic. In the replacement HGP, cancer cells “replace” the hepatocytes while co-opting the sinusoidal blood vessels at the tumor–liver interface. Patients with colorectal cancer (CRC) liver metastases with a replacement HGP respond poorly to the anti-VEGF-A treatment, bevacizumab, likely because these tumors utilize vessel co-option instead of angiogenesis [119]. By contrast, in desmoplastic liver metastases, the cancer cells are separated from the liver by a rim of desmoplastic tissue in which new blood vessels are formed by sprouting angiogenesis. Desmoplastic CRC liver metastases showed a better response to bevacizumab [119]. Taken together, these observations strongly suggest that HGPs can be used to guide the choice of treatment for individual patients with liver metastases.

Across studies, approximately 50% of patients with CRC liver metastases present with a predominant replacement growth pattern and this proportion extend to 95% when patients with breast cancer liver metastases are considered [119, 120]. This clearly demonstrates that non-angiogenic tumor growth is not a rare phenotype. The same applies to primary lung carcinomas and lung metastases for which angiogenic and non-angiogenic HGPs have also been described [121, 122]. In the non-angiogenic, alveolar HGP, cancer cells fill the alveolar spaces and incorporate the capillary blood vessels of the alveolar walls. Approximately, 40% of the lung metastases from clear cell renal cell carcinoma (ccRCC) present with a non-angiogenic HGP despite the fact that nearly all primary ccRCC relies on sprouting angiogenesis, driven by loss of VHL protein function [122].

**Fig. 4:**
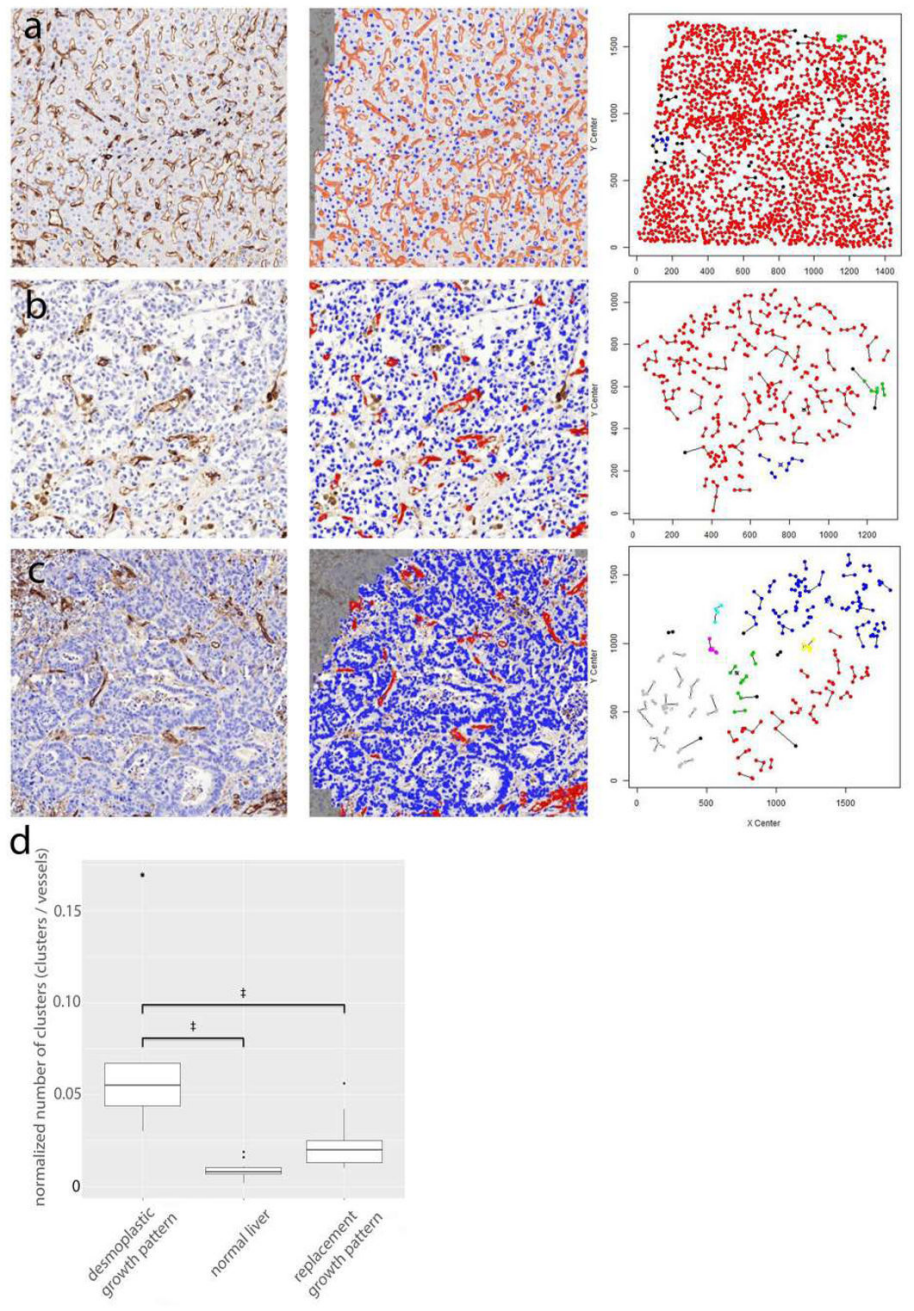
Microvessel density and histopathological growth patterns. **a** Unsupervised spatial modeling of the blood vessel pattern in normal liver shows a low number of clusters per number of vessel profiles. A selected region of interest (ROI) at the tumor–liver interface of normal liver in CD31-stained tissue is shown (left). The Blood Vessel Analysis algorithm of Definiens™ segments and classifies blood vessel objects (orange) and nuclei (blue) (mid). The Cartesian coordinates (*x*, *y*) of the centroids of all vessel objects in one ROI were used in a simplified “SeedLink” clustering method [646] (right). Centroids with the same color (e.g., red) belong to the same cluster. **b** Unsupervised spatial modeling of the blood vessel pattern in a colorectal cancer liver metastasis with a replacement growth pattern shows a low number of clusters per number of vessel profiles. A selected region of interest (ROI) at the tumor–liver interface of replacement growth pattern in CD31-stained tissue is shown (left). The Blood Vessel Analysis algorithm of Definiens™ segments and classifies blood vessel objects (red) and nuclei (blue) (mid). The Cartesian coordinates (*x*, *y*) of the centroids of all vessel objects in one ROI were used in a simplified “SeedLink” clustering method [646] (right). Centroids with the same color (e.g. red) belong to the same cluster. **c** Unsupervised spatial modeling of the blood vessel pattern in a colorectal cancer liver metastasis with a desmoplastic growth pattern shows a high number of clusters per number of vessel profiles. A selected region of interest (ROI) at the tumor–liver interface of desmoplastic growth pattern in CD31-stained tissue is shown (left). The Blood Vessel Analysis algorithm of Definiens™ segments and classifies blood vessel objects (red) and nuclei (blue) (mid). The Cartesian coordinates (*x*, *y*) of the centroids of all vessel objects in one ROI were used in a simplified “SeedLink” clustering method [646] (right). Centroids with the same color (e.g., red) belong to the same cluster. **d** Tukey boxplots of the normalized number of clusters of blood vessel objects for the desmoplastic growth pattern, the replacement growth pattern, and normal liver. There was a statistically significant difference between the growth patterns as determined by one-way ANOVA [*F*(2,22) = 10.8, *p* < 0.001]. A post hoc Tukey test showed that the number of clusters divided by number of vessel objects was significantly different between the desmoplastic growth pattern and the replacement growth pattern (*p* < 0.05, ‡), but also between the desmoplastic growth pattern and normal liver (*p* < 0.001,‡). However, no difference was found between the replacement growth pattern and normal liver (*p* > 0.05). Outliers are plotted as points (·) and extreme values are plotted as asterisks (*)

The prevalence of non-angiogenic tumors and their resistance to anti-VEGF treatment require the identification of a biomarker that accurately reflects this type of tumor growth. HGPs constitute a good candidate biomarker. The vascular pattern in a tumor section immunostained with pan-endothelial antibodies and the use of antibodies that mark EC participating in sprouting angiogenesis are other potential histopathological methods to distinguish non-angiogenic from angiogenic tumors. Indeed, when the number of hot spots is determined by nearest neighbor analysis in whole-slide digital images of liver metastasis tissue sections stained for CD31, the non-angiogenic, replacement-type metastases clearly resemble normal liver tissue. This contrasts with angiogenic, desmoplastic liver metastases, which show a significantly higher number of vascular hot spots than normal liver tissue and their non-angiogenic counterparts (Fig. 4).

Several groups have identified “tumor endothelial markers” or TEMs [123–125]. These are proteins that are selectively upregulated on tumor endothelium compared to normal endothelium. Tumor angiogenesis meta-signatures have been generated using gene expression data of several tumor types by using “seeds” of transcripts, which were known to participate in angiogenesis and to be expressed by EC. The resulting set of EC/angiogenesis-related transcripts that correlated with these seeds was then purified by selecting genes that were modulated in response to anti-angiogenic treatment [126]. Interestingly, several of the top ranking genes in this signature have been confirmed as TEM by other independent teams [127]. Although we do not currently know whether TEMs are expressed equally on both angiogenic tumor vessels and co-opted vessels in tumors, it will be intriguing to assess the expression of these genes, for example ELTD1, CLEC14a, and ROBO4 in the vasculature of replacement versus desmoplastic liver metastases. In primary and secondary non-angiogenic human lung tumors, only preexisting vessels, with an LH39-positive basal membrane and weak or absent αvβ3 integrin, and arising from the alveolar septa entrapped by the neoplastic cells, were observed [128].

The HGP is not a static phenotype: Systemic treatment of patients with CRC liver metastases can alter the HGP [119]. The fact that the HGPs can change in response to treatment reflects the dynamic nature of tumor vascularization driven by either angiogenesis or vessel co-option. This illustrates the necessity for longitudinal assessment of vascularization mechanisms in tumors during treatment and follow-up of individual patients. In order to achieve this, the histopatho-logical evaluations of the tumor vasculature as described here must be supplemented by medical imaging techniques and/or assays, which utilize small biopsies, circulating tumor cells, endothelial cells, or circulating tumor derivatives. Moreover, the importance of an accurate biomarker of ongoing sprouting angiogenesis or non-angiogenic vessel co-option is corroborated by the extensively documented and strong link between angiogenesis and immune suppression [129–131] and the surge of clinical studies that combine anti-VEGF and immunotherapy strategies [132]. In this regard, studies suggested that interactions between endogenous galectins and glycans may link tumor immunosuppression and angiogenesis, conferring resistance to anti-VEGF treatment [133].

### 6.2 Concluding remarks

Although the assessment of MVD has a prognostic value in many different tumor types, this parameter cannot distinguish angiogenic and non-angiogenic processes of vascularization. However, the histopathological growth patterns of, for example, lung and liver tumors accurately reflect the means of vascularization, being sprouting angiogenesis or non-angiogenic co-option of existing blood vessels. We therefore propose to determine both the HGP and MVD when studying the vascularization of tumors. In addition, noninvasive surrogate markers of the HGP should be developed, for example medical imaging parameters, and these markers should be integrated in clinical oncology trials.

## 7 Assessment of intussusceptive angiogenesis

Angiogenesis is the de novo formation of blood vessels and can follow either a sprouting or a non-sprouting course. One important non-sprouting mechanism is intussusception (growth within itself; known also as vascular splitting). Although both sprouting and intussusception lead to an expansion of the capillary network, the processes involve different cellular mechanisms, which are presumably regulated by different molecules. Sprouting angiogenesis (SA) is characterized mainly by local vasodilatation, increased vascular permeability, and cell proliferation. It is initiated by proteolytic degradation of the basement membrane, which is followed by migration of proliferating EC into the ECM. The sprouts reorganize internally to form a vascular lumen and to finally connect to other capillary segments [134]. Intussusceptive angiogenesis proceeds through transluminal tissue pillar formation and subsequent vascular splitting. The direction taken by the pillars delineates intussusceptive angiogenesis into its overt variants, namely: (1) intussusceptive microvascular growth (expansion of capillary surface area), (2) intussusceptive arborization (remodeling of the disorganized vascular meshwork into the typical tree-like arrangement), and (3) intussusceptive branching remodeling (optimization of local vascular branching geometry), including intussusceptive vascular pruning. It has been shown that intussusceptive angiogenesis takes place not only during physiological processes, but also under pathological conditions including tumor growth, colitis, and in neurodegenerative disease, among others [135]. Although the morphological facets of intussusceptive angiogenesis as well as molecular mechanisms of sprouting are well described, the exact cellular and molecular mechanism of intussusceptive angiogenesis is not yet characterized.

### 7.1 The concept of intussusception and the methodological challenge

The concept of intussusceptive angiogenesis was first described within the rapidly expanding pulmonary capillary bed of neonatal rats [136]. Morphological investigations during the last three decades have indicated that the transluminal pillars arise into the vessel lumen according to the four consecutive steps in pillar formation postulated by Burri et al. in 1990: (1) creation of a zone of contact between the opposing sides of the capillary wall; (2) reorganization of the junctions between endothelial cells in the contact zone, resulting in central perforation of the bilayer; (3) formation of an interstitial pillar core, which is invaded first by the cytoplasmic processes of myofibroblasts and pericytes, and then by collagen fibrils; and (4) the pillars expand in girth (≥ 2.5 μm) and transform into a capillary mesh. The described model with the four consecutive steps is based mainly on transmission and scanning electron microscopy methods (scanning or transmission electron microscopy), and these techniques do not allow dynamical observation of the pillar formation, fusion, and vascular splitting over time.

### 7.2 Limitations and challenges

Intussusceptive angiogenesis is spatially quite a complex process, and it is not possible to document it on isolated sections. To overcome this limitation and maintain high resolution, a 3D reconstruction based on serial ultrathin sections was reported for the first time in year 2000 [137]. This is a very laborious approach feasible only in specialized laboratories. Even with significant expertise, only limited numbers of pillars as a qualitative and descriptive illustration can be visualized. A new technique is now available that makes such task much more feasible—serial block face 3D imaging. With this new technique, exact morphological information on relatively big sample volumes (1 × 1 × 1 mm) within a short time can be acquired: For example, the scanning of a block the size of one zebrafish embryo would take around 1 day and would provide a 3D scan of the complete embryo at ultrastructural level. Using the standard approaches, it would have taken weeks, if not months. Moreover, the outcome from 3View is of much better quality and without loss of information (slices). This technique enables a quantum leap forward in 3D morphological studies [138].

**Fig. 5:**
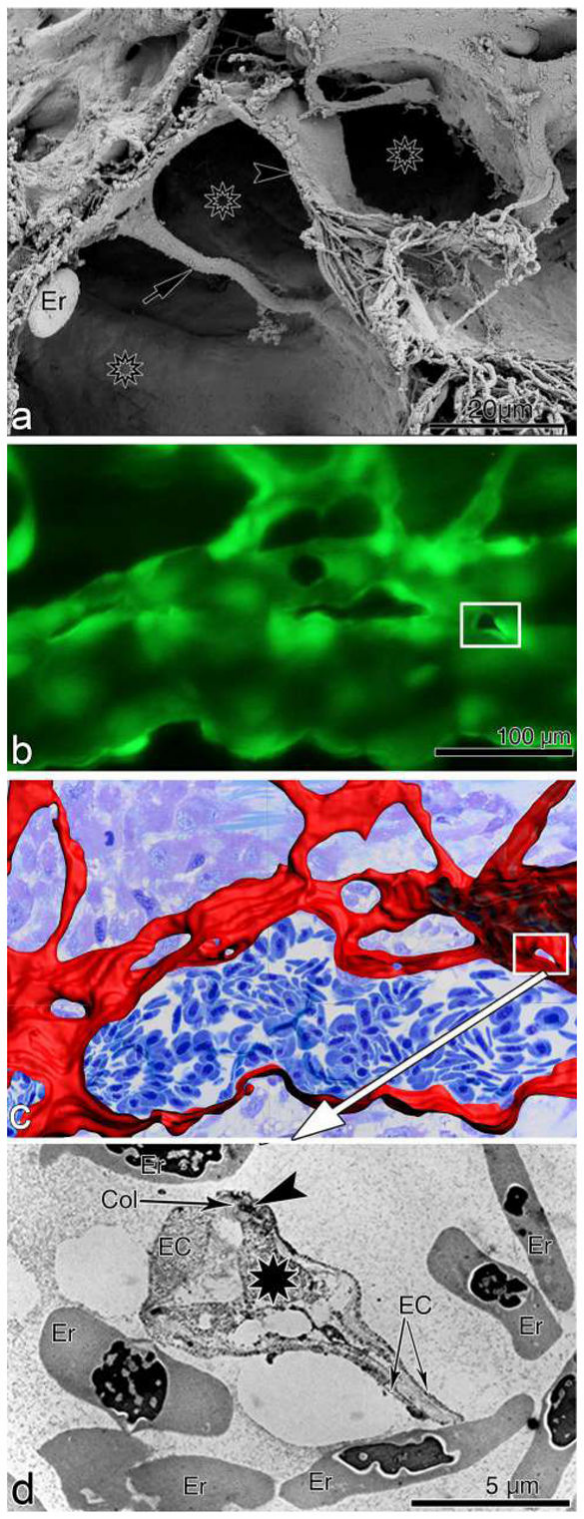
Intussusceptive angiogenesis—the methodological challenge. **a** Scanning electron microscopy image of transluminar pillars. Early stage is characterized by tinny pillar (arrow), formed mainly by endothelial protrusions coming from the opposing EC. At later stages, the pillar (arrowhead) is increasing in girth and its core is invaded by perivascular cells, fibroblasts, and fibers (visible in the lower disrupted part of the pillar). Lumen of the vessel is marked with asterisks. *Adapted from* [647]. **b** Dynamic in vivo observation of the regenerating zebrafish fin vasculature demonstrated a newly formed pillar (rectangle). **c** Three-dimensional reconstruction based on serial semi-thin sections from the same area depicted in **b**, **d** transmission electron micrograph demonstrates the transluminal tissue pillars (rectangle in **b**, **c**) at ultrastructural level. Black asterisk indicates the core of the pillar, while arrowhead pointed to cell–cell contacts between the endothelial cells (EC). *Er* erythrocyte, *Col* collagen fibers. Adapted from [648]

*Vascular casting* has been extensively used to demonstrate intussusceptive angiogenesis in 3D. The methodology is based on creating a vascular replica of the microvasculature. As casting media Methylmethacrylate (Mercox, Japan Vilene) or PU 4ii (VasQtec, Switzerland) is used [137]. A few hours after perfusion, the organs are excised and transferred to 15% KOH for 2–4 weeks for dissolving the tissue. After washing, the casts are dehydrated in a graded series of increasing ethanol concentrations and dried in a vacuum desiccator. They are then examined using SEM. A new powerful methodology employing polymer-based contrast agent in combination with micro-computed tomography (micro-CT) has been reported recently [139]. Both casting and micro-CT are very useful for evaluation of large samples, for example entire mouse organs and in a subsequent step to “zoom” in on the area of interest. The vascular casting combined with scanning electron microscope has the potential to demonstrate greatest morphological details (Fig. 5a). The micro-CT does not provide a high level of resolution, but is very rapid and the epitopes are not destroyed and can be used for further morphological investigations [139].

Dynamic in vivo observation of the pillar formation and subsequent vascular splitting over time is feasible employing conventional fluorescence microscopy, stereomicroscopy, and laser scanning microscopy (Fig. 5b). The latter methodologies are widespread, but often cause partially severe, phototoxic artefacts. Zebrafish lines with uniform cytosolic expression of the respective fluorescent proteins such as Tg(Fli1a:eGFP)//Tg(Gata1:dsRed) represent a useful model for dynamic observation of vascular splitting and blood flow (Circulating erythrocytes). Zebrafish lines with uniform cytosolic expression of the respective fluorescent proteins can be used, for example Tg(Fli1a:eGFP)//Tg(Gata1:dsRed). The embryos are mounted in low-melting agarose in the presence of phenylthiourea and can be monitored for 16–20 h. To reduce phototoxicity, imaging should be performed with laser scanning microscopes with a long working distance, using a heated chamber, fast scanning (less than 1 μs pixel time), lowest possible laser power (in a range of few percentages depending on the microscope), and time steps of 10–15 min. Such a dense time pattern is necessary to record the dynamic cellular alterations of pillar formation and splitting. Using these settings, no signs of photobleaching or phototoxicity (membrane blebbing, cell apoptosis) have been observed.

### 7.3 Concluding remarks

Due to its 3D complexity, intussusceptive angiogenesis can only be properly investigated by a combined methodological approach. Matching the dynamic cellular changes in capillaries obtained with in vivo time-lapse studies with morphological data from light microscopy level up to the ultrastructural level by transmission electron microscope can provide complex and detailed information (Fig. 5b–d). The sites of interest should be documented first by intravital microscopy as described above. After the in vivo documentation, the area of interest can be harvested, fixed, and processed either for paraffin sectioning and LM with immunohistochemistry or alternatively for serial semi-thin sectioning and 3D analyses using, for example, Imaris Software. To obtain a deeper insight into the morphological substrate and tissue components involved in the process, the same tissue should be processed for transmission electron microscope (Fig. 5).

## 8 In vivo sprouting lymphangiogenic assay and AAV-mediated gene transfer of vascular endothelial growth factor c (VEGFC)

The lymphatic system plays a key role in the body in maintaining tissue fluid homeostasis, in lipid absorption, and in immune cell trafficking. In some life-threatening diseases, such as cancer, lymphangiogenesis, the formation of new lymphatic vessels, contributes to disease progression. By contrast, inherited or acquired insufficiency of lymphatic vessel development results in various forms of lymphedema [140, 141]. The most important receptor signal transduction system guiding lymphatic growth is the VEGF-C/VEGFR-3 system. Increasing tissue concentration of VEGF-C by transgenic overexpression or viral gene delivery system leads to lymphatic overgrowth [142, 143]. By contrast, overexpression of soluble VEGFR-3 extracellular domain leads to suppression of lymphatic vessel growth in cancer models [144]. Thus, stimulation or suppression of lymphatic vessel growth in experimental animals and in human patients can be the ultimate way to improve or inhibit lymphatic function. In this section, a description is provided of two in vivo techniques that allow studying the lymphangiogenic process. One is called the ear sponge assay, and the other one describes viral gene delivery techniques to achieve the same goals. Several viral gene delivery vectors are known to date, such as adenovirus, adeno-associated virus (AAV), lenti/retrovirus, and baculovirus. Here, we describe as an example, the use of AAV for the transfer and analysis of lymphatic vascular growth factors.

### 8.1 Description of the ear sponge assay and AAV generation/tissue transduction

**Fig. 6:**
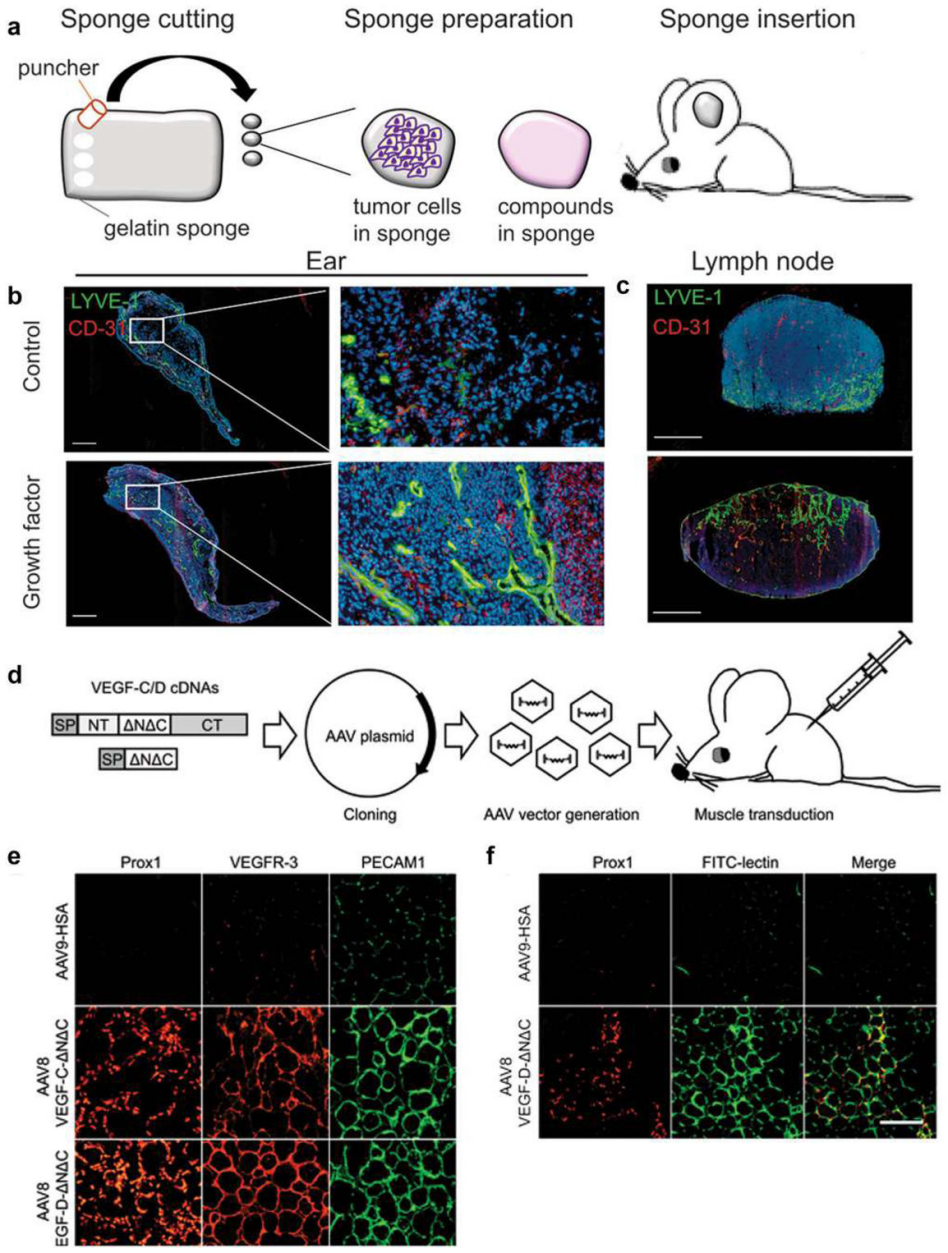
Stimulation of lymphatic and blood vessel growth in vivo. **a**–**c** Preparation of gelatin sponges for lymphangiogenic assay. **a** Small pieces of gelatin sponge are detailed with a puncher before to be soaked with tumor cells or a compound. Prepared sponges are inserted in mouse ear between the two skin layers. **b**, **c** Immunohistochemical analysis of sponges (**b**) and sentinel lymph node (**c**) resected from mouse with control sponge (PBS or medium without growth factor) or from mouse with sponge imbedded with growth factor. Lyve-1 (lymphatic endothelial cell marker) is stained in green, and CD-31 (blood endothelial cell marker) is stained in red. Sponges soaked with growth factor showed higher lymphangiogenesis and angiogenesis compared to control sponge. Scale bar in **b**, **c**—250 μm. **d**–**f** Preparation of AAV and transduction of skeletal muscle. **d** Schematic representation of different VEGF-C and VEGF-D isoforms, produced by step-by-step proteolysis, and general AAV production and usage protocol. **e** Immunohistochemical analysis of t.a. muscle transduced with AAV8 encoding VEGF-C-ΔNΔC or VEGF-D-ΔNΔC. Tibialis anterior muscles of C57BL/6 J male mice (8 weeks old) were injected with 10^9^ AAV8 particles in 30 μl of PBS, and the mice were euthanized 2 weeks later. T.a. muscle samples were isolated and analyzed immunohistochemically for the indicated markers. *HSA* human serum albumin. **f** Analysis of the functionality of lymphatic vessels. Lectin (from Lycopersicon esculentum), conjugated with FITC (FITC-lectin) was injected to the distal part of t.a. muscle. After 45 min, the mice were euthanized and t.a. muscle was isolated, fixed, and stained for Prox1. Lectin is visualized by FITC. Scale bar in **e**, **f**—50 μm

In this model, sprouting lymphangiogenesis is reproduced in a complex in vivo microenvironment. This application is based on the use of a biomaterial, gelatin sponge that is inserted in the mouse ear to develop a large lymphatic network [145]. The gelatin sponge is cut into pieces of about 3 mm^3^, which are transferred in a 96-well plate (one sponge piece per well) for pre-treatment (Fig. 6a). The sponge can be soaked with tumor cells, cell-conditioned medium, lymphangiogenic growth factors or inhibitors. Cell suspension or compound solution (20 μl) is seeded on the top of the sponge. The plate is incubated at 37 °C, for 30 min, to allow the diffusion of the solution into the biomaterial. Sponges are next embedded in a type I collagen solution (7.5 volumes of interstitial type I collagen solution, 1 volume of 10 × Hanks’ balanced salt solution, 1.5 volume of 186 mM NaHCO3, pH adjusted at 7.4 with a 1 M NaOH solution) and placed in a new well for 30 min to allow collagen polymerization. For sponge implantation into mice, a small incision is made in the basal, external, and central parts of the mouse ear. Ear skin layers are slowly detached with forceps to make a hole for sponge insertion, which is then suture shut. Mice are killed at different time points (2–4 weeks post-sponge implantation) to evaluate lymphangiogenesis in the sponge and lymph nodes (Fig. 6a–c).

Preparation of the recombinant AAV encoding VEGF-C or VEGF-D of mouse origin starts from the cloning of the ORF into the special AAV plasmid vector, which possesses flanking sequences 5′ and 3′ Inverted Terminal Repeats (ITRs) (Fig. 6d). Such vectors can be obtained from commercial sources, for example from Addgene (www.addgene.com). Next, the AAV plasmid vector is used to generate AAV particles by transfecting the plasmid into the host cell lines derived from embryonic human kidney 293 cells. We suggest using three-plasmid transfection system, in which AAV and adenovirus helper genes are administered into 239T cells together with the VEGF-C/VEGF-D encoding AAV vector plasmid by simultaneous transfection. 293T cells are seeded at an initial density of 7.7–10^6^ cells/145 cm^2^plate. When reaching cell confluency level of 70–80%, cells are transfected with three plasmids (gene encoding one and the two are the helper plasmids) (1:1:1 molar ratio). The AAV accumulating inside the cells are released from the cells by three consecutive freeze/thaw cycles using liquid nitrogen and + 37 °C water bath, respectively. AAV particles are then purified and concentrated by ultracentrifugation. It is important to run a functional test to assess the ability of the AAV particles to transduce mammalian cells and drive expression of transgenic proteins. This can be done by transfecting cultured 293T cells and determining the expression of a transgene by any suitable method, such as Western blotting, ELISA, or fluorescent microscopy in case of fluorescent proteins.

For the treatment of skeletal muscle (tibialis anterior, t.a., in mice), the volume of the AAV solution should be kept to a minimum and should not exceed 50 μl/t.a. for the adult mice of ≥ 7–8 weeks old. The AAV dose (concentration of AAV particles in the injected volume) for a single injection is subject to thorough consideration, taking into account various factors, such as susceptibility for AAV transduction in a certain tissue, biological activity and toxicity of the transgene, half-life of the transgenic protein in free circulation or in a tissue. Gelatin sponge transplantation and injection of AAV solutions are performed under either isoflurane or liquid (ketamine/xylazin) anesthesia. Transgene expression starts soon after the treatment and becomes detectable in 1–2 days, increases until it reaches the plateau (in 5–7 days), and can slightly decline later on, but never shuts down completely.

### 8.2 Applications, benefits, and strengths of the assay: analysis of lymphangiogenesis and angiogenesis

Lymphatic vessels are visualized by immunohistochemical staining using antibodies against, for example LYVE1, Prox1, or VEGFR3, which are relatively specific for lymphatic EC (Fig. 6b, c, e, f). As the mouse ear displays also a high blood vessel density, especially after angiogenic stimulation, angiogenesis can be analyzed by using anti-CD-31 antibody. In addition, lymphatic function prior to and after the AAV treatment can be studied in mice under anesthesia by injecting a lymphatic tracer [such as FITC-lectin (FL-1171; Vector Laboratories)] to the distal part of the muscle as the tracer is taken up by the lymphatic vessels and carried by lymph flow. The moving front of the tracer can be observed and recorded and serves as a measurable parameter of the functional lymphatics. The tracer can be also visualized by immunohistochemical staining, combined with simultaneous co-staining for lymphatic endothelial marker(s) (Fig. 6e, f).

#### Lymphatic remodeling in sentinel lymph node

The ear sponge assay applied with tumor cells mimics the early steps of cancer progression and the establishment of lymph node pre-metastatic niche [146, 147]. The collagen capsule provides a barrier that retains cells in the sponge. Lymphatic vessels present in the ear drain factors to lymph nodes leading to a remodeling of the lymphatic vasculature in the sentinel lymph node prior to tumor cell arrival (Fig. 1c). Lymph node metastases are detected between 3 and 4 weeks post-implantation (depending on tumor cell types and the number of cells used).

#### AAV gene delivery provides stable transduction

Because of low immunogenicity, AAV itself does not provoke a strong immune response, in sharp contrast to in vivo transduction with adenovirus, which activates the NF-KB pathway. Unlike AAV, adenovirus gene delivery is accompanied by a significant immune response to the adenovirus-encoded proteins, resulting in the shutdown of transgene expression due to host immune attack. This feature of AAV allows long-lasting tissue transduction in mice for up to 2 years. If the transgene delivered to the mice via an AAV vector encodes an isogenic murine protein, it does not provoke an immune response either. Although the purified transgenic proteins can also be used for in vivo treatment, their administration into the tissues usually produces an inferior effect due to the instability and fast dilution of the injected protein when it diffuses out from the site of injection. It should be noted that lymphangiogenic and angiogenic proteins must be present for a prolonged period of time to allow vessel formation and maturation of the newly formed vessels. Immature vessels often formed after adenovirus-mediated gene delivery are unstable and prone to regress soon after the transgene expression ceases [148]. Multiple injections of the protein to the same tissue are not desirable due to the trauma caused by the injections.

#### Analysis of various forms of lymphangiogenic factors

Various forms of VEGF-C or VEGF-D can be used including immature (full-length) pro-VEGF-C/VEGF-D and mature VEGF-C/VEGF-D-ΔNΔC forms, which are naturally produced during step-by-step proteolysis (Fig. 6d). Only mature forms VEGF-C/VEGF-D-ΔNΔC demonstrate full lymphangiogenic activity in vitro and in vivo, primarily due to higher affinity interactions with VEGFR-3. In nature, they are all expressed as immature pro-VEGF-C/VEGF-D, which then undergo step-by-step proteolysis producing fully active mature ΔNΔC forms. Thus, AAV-mediated expression of ΔNΔC form of VEGF-C or VEGF-D provides somewhat artificial stimulatory conditions for lymphatic vessel growth.

#### Lymphangiogenic factors can also stimulate blood vessel growth

The mature forms of both VEGF-C and VEGF-D are able to bind to and stimulate not only VEGFR-3, but also VEGFR-2, although the affinity toward VEGFR-2 is significantly lower than VEGF-A (by a factor of 20–100). Blood vessel growth is induced when AAV-VEGF-C or AAV-VEGF-D is administered to the murine skeletal muscle [143], which has also been demonstrated in rabbit skeletal muscle transduced by recombinant adenoviruses [149]. The angiogenic effect of VEGF-C, however, can be decreased, while preserving full lymphangiogenic activity, by using a VEGF-C156S mutant form of the growth factor [150]; VEGF-C156S has a reduced binding affinity for VEGFR-2, while binding affinity to VEGFR-3 is largely unaltered.

### 8.3 Limitations and challenges

#### Sponge preparation

Steps related to sponge preparation are critical for the reproducibility of the assay. The addition of a drop of 20 μl with cells or factors on the top of the sponge is better than an immersion of the sponge in the solution. Moreover, it is important to turn the sponge halfway through the incubation period to allow homogenous diffusion in the biomaterial. Finally, the collagen capsule formation is a key step to allow sponge insertion between ear skin layers without cell or solution loss.

#### Control of transgene expression

To date, broad usage of AAV-mediated overexpression of VEGF-C/VEGF-D (or VEGFR-3-Fc) in mice or in human patients in clinical practice is hindered by the inability to efficiently regulate transgene expression. When a lymphangiogenic effect is achieved, it would be desirable to shut off the expression of the transgene. Long-term overexpression of VEGF-C/VEGF-D would lead to a heavily overgrown and unstructured lymphatic vasculature, which would be poorly functional. Blood vessels may also respond to long-term, uncontrolled VEGF-C/VEGF-D overexpression by overgrowth and increased leakiness. Currently existing ON/OFF switch systems, such as under tetracycline regulation, are either inefficient, or toxic for humans, although some have been adapted for in vitro use. The versatility of the CRISPR/Cas9 delivery system may provide solutions to these problems in the near future.

#### Unequal transduction of target cells in the transduced tissue

Unlike in transgenic animals, where transgene expression can be induced in virtually every cell in a target tissue such as muscle, viral gene delivery fails in some of the target cells and this can lead to tissue mosaicism and non-homogenous protein delivery. Cell types vary in their sensitivities to AAV transduction due to differential expression of AAV-binding receptors. For example, tail vein injection of AAV9 results in a significantly higher transduction of heart than of the liver or pancreas [151].

#### Local trauma associated with ear sponge implantation and viral vector delivery

During surgery, the integrity of the ear must be assessed as perforations can result in sponge loss. Of note, it is not possible to identify mice using ear clips since both ears are used for the assay. Depending on the pathological context of the study, a limitation of the ear sponge model is local inflammation induced by the sponge insertion. Regarding viral vector delivery, when AAV is administered locally, the injection itself produces some trauma in the target tissue, which can lead to altered sensitivity of cells along the vector injection path.

### 8.4 Concluding remarks

The ear sponge assay is a suitable model to study lymphatic vessel remodeling in the both primary tumor and sentinel lymph node prior (pre-metastatic state) and after tumor cell arrival (metastatic state). Gene delivery via AAV represents a reliable and relatively inexpensive approach for gene therapy in inherited or acquired vascular disease models. AAV provides a convenient way to study biological properties of bioactive proteins, such as vascular growth factors in vivo and under conditions mimicking the natural expression of the endogenous genes.

## 9 Assay for pericyte recruitment to endothelial cell-lined tubes, capillary assembly, and maturation

**Fig. 7:**
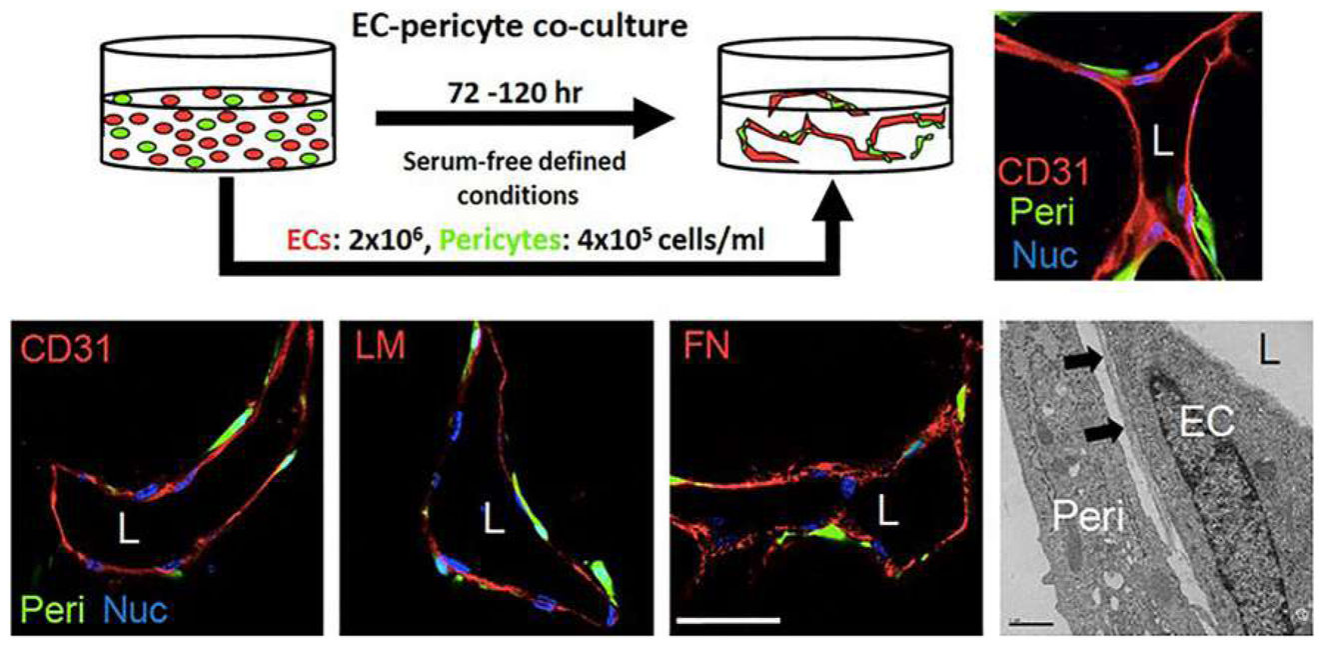
Serum-free defined model of human endothelial cell-pericyte tube co-assembly in 3D collagen matrices. **a** Human EC and GFP-labeled pericytes (Peri) were seeded together at the indicated cell densities and after 120 h, were fixed and stained with anti-CD31 antibodies. The immunostained cultures were imaged using confocal microscopy. The serum-free defined culture system contains SCF, IL-3, SDF-1α, FGF-2 and insulin (a component of the RS II supplement), which are required to be added in combination. **b** Cultures fixed at 120 h were immunostained with antibodies to CD31, laminin (LM), and fibronectin (FN) and were imaged using confocal microscopy or were examined by transmission electron microscopy. Arrows indicate the capillary basement membrane. L indicates lumen while Nuc indicates nuclear labeling. Bar equals 25 μm

A fundamental question concerns the growth factor and signaling requirements governing how human EC and pericytes co-assemble to form capillary tubes in a 3D matrix or tissue environment [80, 152–155]. To this end, bioassays have been developed in 3D collagen or fibrin matrices to address such issues using serum-free defined conditions [156, 157] (Fig. 7). In either system, pericyte recruitment to EC tubes can be readily observed over a 72–120 h period and a consequence of this recruitment is deposition of the vascular basement membrane between the abluminally recruited pericytes and the ECs that line the branched networks of tubes [153]. Interestingly, EC tube formation results in the creation of networks of ECM spaces, which are termed vascular guidance tunnels [158], where the EC tubes reside, and are also a physical space into which pericytes are recruited along the basal and abluminal EC tube surface [153, 158]. Both EC and pericytes migrate along each other in a polarized manner within these tunnels, resulting in basement membrane deposition underneath the abluminal surface of the EC-lined tubes [152, 153, 159]. Basement membrane deposition leads to much narrower and elongated EC tubes in EC-pericyte co-cultures compared to EC-only tubes which progressively get wider and less elongated with time [153, 159].

### 9.1 Benefits and strengths of the assay

The major benefit and strength of this assay model is its high reproducibility and high replicate number in that it is performed in half-area 96-well plates. The assay is typically set up in 60 wells per plate, and this can be readily established using one T75 flask of human EC (e.g., HUVEC) and one T25 flask of human pericytes (brain-derived vascular pericytes) (labeled with GFP). The assay allows performance of siRNA suppression experiments using either cell type or lysates that can be prepared to assess signaling transduction cascades by Western blot. Also, the recruitment of pericytes can be examined in time with or without blocking antibodies to various targets (e.g., growth factors, receptors, integrins, ECM molecules, etc.) or signal transduction inhibitors. The consequence of pericyte recruitment to EC tubes can be revealed by examining basement membrane deposition (by immunofluorescence staining or transmission electron microscopy) (Fig. 7), or by measuring capillary tube widths and lengths. Finally, real-time video analysis can be used to monitor pericyte and endothelial motility as they co-assemble to form tube networks with abluminally recruited pericytes in 3D matrices.

### 9.2 Assay overview

HUVEC or human artery endothelial cells (HUAECs) and human brain-derived vascular pericytes or bovine retinal pericytes are trypsinized and seeded within 3D collagen matrices at a density of 2 × 10^6^ cells/ml for EC and 4 × 10^5^ cells/ml for pericytes. Both types of EC or pericytes work very well in this assay system. FGF-2 and stromal-derived factor-1α (SDF-1α) are each added at 200 ng/ml into the collagen gel mix (2.5 mg/ml of rat collagen type I). Twenty-five μl of gel is added into each individual 96 well (A/2 plates from Costar). After 30 min of equilibration in a CO_2_ incubator, culture medium is added (100 μl per well) which is Medium 199 (100 μl per well supplemented with 1:250 dilution of Reduced Serum Supplement II) [160], and 40 ng/ml each of FGF-2, stem cell factor (SCF), interleukin-3 (IL-3), and 50 μg/ml of ascorbic acid are added. Cultures are then placed in a CO_2_ incubator for 72–120 h. After this time, cultures are fixed with 3% paraformaldehyde in PBS (120 μl per well). Cultures can then be imaged under fluorescence to quantify EC tube area and pericyte recruitment under the various conditions of treatment. Individual cultures can be immunostained with various antibodies directed to EC or pericyte cell surfaces and/or ECM antigens (i.e., basement membrane proteins such as fibronectin, laminin, and collagen type IV) [153]. Alternatively, cultures can be used to make lysates using SDS sample buffer or used to isolate total RNA to perform gene expression experiments [153]. Real-time movies can be performed to examine pericyte recruitment and measure pericyte motility during capillary assembly and maturation [154]. In the latter case, nuclear GFP-labeled pericytes can be utilized to track pericyte motility [154].

### 9.3 Critical steps in the assay

The assay system above is highly dependent on the health of the growing EC. To each 500 ml bottle of Medium 199, we add 200 mg of lyophilized bovine hypothalamic extract, 50 mg of heparin, and 100 ml of fetal calf serum (then sterile filter complete media). Tissue culture flasks are coated with 1 mg/ml of gelatin solution for at least 30 min. prior to seeding the trypsinized EC. Pericytes are grown using the same culture media and gelatin-coated flasks. Bovine hypothalamic extracts as well as rat tail collagen preps are used at 7.1 mg/ml protein in 0.1% acetic acid. The assay system is highly robust and reproducible [153, 156, 157].

Especially for longer-term experiments (i.e., multiple days), it is important to completely surround each well with 100 μl of water. This reduces dehydration of media in the microwells and maintains the health of the cultures. For cultures extending longer than 72 h, the cultures are replenished by removing 90 μl of media and replacing with 100 μl of media per well. This medium is slightly different than that used above to start the assay: Medium 199 containing a 1:250 dilution of RS II, ascorbic acid at 50 μg/ml, as well as 80 ng/ml of FGF-2, and 40 ng/ml each of SCF, IL-3, and SDF-1α.

### 9.4 Limitations and challenges

The major limitations of this in vitro assay relate to how reflective the assay model is to the in vivo state. Overall, the data and conclusions obtained over the years have been repeatedly demonstrated to be observed using mouse, zebrafish, or quail in vivo models [155]. One of the interesting issues is the state of differentiation of the EC that are used in these in vitro assays, and how similar are they to EC in vivo that are undergoing vasculogenesis or angiogenic sprouting. This is a very difficult question to answer and needs to be addressed in future studies. Another potential weakness of the current in vitro systems is that flow conditions have to be adapted or the gels have to be transplanted into a mouse model to interface the human vessels with an in vivo vascular system.

### 9.5 Concluding remarks

Here, a defined assay system is described which allows for the assembly of capillary tube networks, with EC-lined tubes and abluminally recruited pericytes in 3D collagen or fibrin matrices. This system has been previously utilized (and can be employed in future studies) for detailed investigations of the molecular and signaling requirements for this process, by independently assessing the functional role of ECs vs. pericytes during these tube co-assembly events.

## 10 EC co-culture spheroids

Angiogenesis, the sprouting of endothelial cell capillaries from preexisting blood vessels, is multifactorial including processes such as proliferation and directed migration. These processes in turn are influenced not only by autocrine but also by paracrine stimuli mediated by, for example, pericytes, fibroblasts, immune cells, and tumor cells in the case of cancer. This intercellular communication by stimuli includes growth factors, chemo- and cytokines as well as other secreted factors, like enzymes, extracellular basement membrane components, and the exchange of genetic material (miRNA, mRNA, DNA) via extracellular vesicles [161–163]. Anatomically, these various cell types are fine-tuned and orchestrated to become organized 3D structures to ultimately allow and control the flow of blood. However, the field of angiogenesis has typically focused on studying angiogenesis-associated processes in vitro in pure EC cultures. Although studying EC in isolation has some advantages in investigating certain EC-specific elements and to certain defined processes within angiogenesis, 3D co-culture spheroids allow one to study the direct intercellular cues and cross talk between EC and their surrounding cells. Co-cultures with tumor cells create a simplified dynamic tumor microenvironment due to the changes in shape and size in time. As a result, the 3D spheroid assay sheds light on the biological conduct and communication of EC with a multitude of different cell types under various stresses or treatment conditions in a controlled manner. Importantly, it offers the possibility to investigate multiple different interventions (e.g., chemotherapies, radiation, experimental drugs) in a medium-throughput manner in an environment more relevant to the in vivo situation.

### 10.1 Strengths and benefits of the three-dimensional co-culture EC spheroids

Studies on in vitro 2D monolayer cell cultures and their translation/extension to clinical settings have their limitations, as they are not capable of mimicking the nutrient and oxygen gradient and an environment reminiscent of the in vivo setting. The preclinical 3D co-culture spheroid assay facilitates intra-/intercellular cross talk mimicking aspects of in vivo vascular architecture, for example lumen formation and polarization, and is particularly valuable for interrogating these interactions in the presence of two or more cell types growing together with natural adhesion and layering properties. Additionally, and at least as important, it lends itself for controlled experimental manipulation and replication thereof as it can be applied in the developmental/regeneration field as well as the cancer biology and the cancer treatment fields. Particularly in the cancer field, the 3D co-culture tumor cell/EC spheroid assay is a valuable instrument for interrogating interactions between the tumor stroma and parenchyma to better understand the mechanisms of radiation and cancer therapeutics and how cells support or damage each other in their response to these interventions. Ultimately, the ability to screen a relatively high number of conditions against these mini-tumor microenvironments has potential to promote the establishment of improved drug pharmacokinetics, efficacy, and safety profiles [164].

Inherent in many in vitro assays, one of the strengths of the spheroid assay is the relative ease of focused manipulation to aid in answering specific hypotheses. Namely, prior to the paired co-culture one of the cell types can be genetically altered by retro- or lenti-viral constructs, including fluorescence (gain-of-function) or by shRNA/siRNA knockdown or CRISPR/Cas9 (loss-of-function). Additionally, the sensitivity or resistance to a particular pharmacological or radiation treatment approach can be tested. We and others have observed marked differences in treatment response and viability of tumor cells growing in the presence/in contact with EC and vice versa [164].

### 10.2 Assay overview and types of assays

**Fig. 8:**
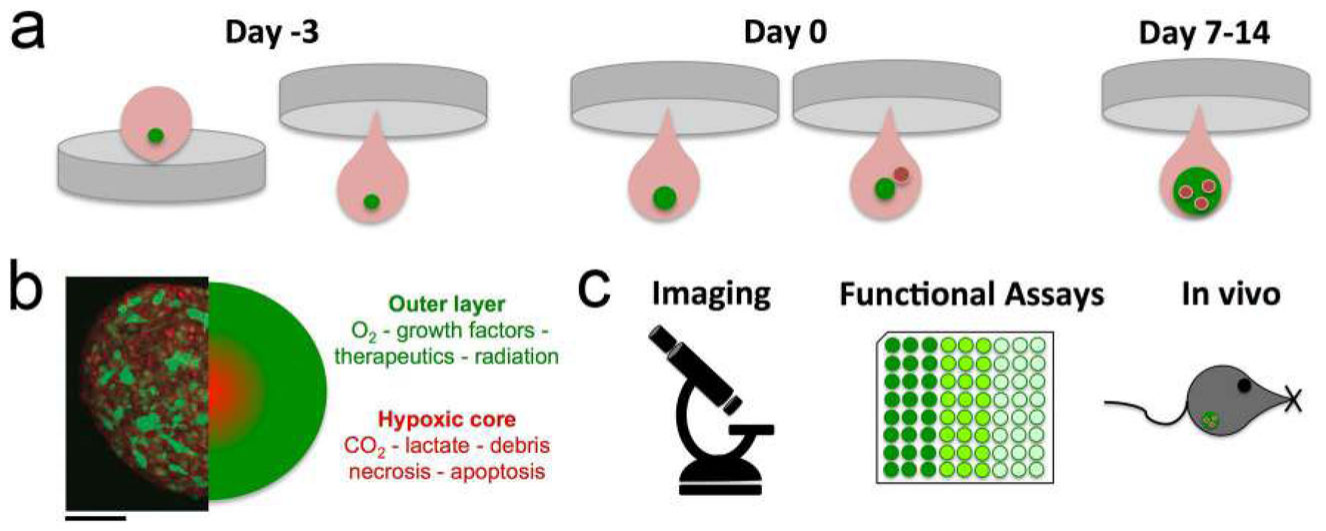
EC co-culture spheroid assay. **a** Overview of the procedure of the hanging drop spheroid growth assay over time. Prior to initiating the co-culture, tumor cells (green) are allowed to establish initial micro-spheroids by themselves. Typically, after 72 h EC can be introduced (red) and their incorporation monitored over time up to 14 days. **b** After 7 days, the tumor-EC spheroid can be quantitatively and qualitatively evaluated by various techniques, such as **c** (fluorescence) microscopy, histology, functional assays or implanted *in vivo*. Breast tumor cells (green; GFP-4T1) are combined with 2H11 EC (red) stained with MitoTracker Red. Scale bar = 100 μm

Multicellular 3D spheroids can be generated by various methods, including the (1) spontaneous spheroid formation using (ultra) low binding plates; (2) “hanging drop” technique; (3) suspension cultures (e.g., by spinner flasks or bioreactors); (4) scaffold-based models (e.g., hydrogels), and (5) magnetic levitation [165]. Since spontaneous spheroid formation and the hanging drop method are arguably the most cost-effective and likely take the least amount of specialized skill set, we will focus on these two iterations of the model (Fig. 8). One of the pioneering approaches to generate self-assembling multicellular 3D tumor endothelial spheroids in hanging drops was by Timmins et al. [166]. Subsequently, this assay was validated by various groups and expanded on in terms of cell types tested and it evolved as an instrument to be transplanted into animals, devoid of Matrigel, to study cancer progression and ultimately metastasis [164].

The initial spheroid structure is produced by pipetting a single-cell suspension of 1000 tumor cells in a 20-μl droplet on the inside of the lid of a, for example, 48-well cell culture plate (Greiner Cellstar, BioExpress, Kaysville, UT). Gravity-enforced self-aggregation is facilitated by inverting the lid and placing it on its receptacle (i.e., the 48-well plate). The culture medium and incubation conditions do not need to be adjusted and thus can stay as preferred. After 72 h, the lids are set inside up and the addition of 2000 EC in a volume of 5 μl is added to the existing culture. The lid is then re-inverted, and the hanging drops can be incubated and monitored for growth, cell type incorporation characteristics, and other aspects for an additional 14 days depending on the cell types used (Fig. 8).

Additionally, the spheroids can be implanted in vivo and used in animal models or within specialized techniques such as the dorsal skin fold window chamber, creating an in vitro*/*in vivo assay. For decades, it has been postulated that growing tumor cell-only spheroids and implanting them in animals lead to a much higher tumor take with far fewer total cells inoculated. This was hypothesized to be due to the creation of a microenvironment in the spheroid as well as possibly engaging or enriching for cancer stem cell phenotype. Although the optimal conditions vary when co-culturing different cell types with EC, when using tumor-EC spheroids, as few as two spheroids per implantation are sufficient to generate tumor growth and metastasis [164]. This approach may be valuable in studying various treatments or aspects of tumor progression thought to be dependent on vascularization and/or stimulation of metastasis or aggressiveness via tumor cells engaging EC.

The spheroids can be assessed by multiple readouts depending on the laboratories preferences, techniques available, and skill set. Namely, quantitative and qualitative evaluations include imaging techniques such as bright field and fluorescence microscopy, molecular marker assessment by, for example, immunohistochemistry or immunofluores-cence, or functional assays such as cell viability and survival assessment with or without growth factors, biologics, therapeutics, or radiotherapy [164]. The functional assays are generally performed after transferring the spheroids into low-binding 96-well plates to better control and standardize the additions of, for example, growth factors, therapeutics, or reporters (e.g., viability or proliferation stains; Fig. 8).

### 10.3 Limitations and challenges

Although in theory this assay can be used for multiple different co-cultures with a number of cell types, some restrictions arise in the use of particular cell types as well as the combinations thereof. Namely, primary isolates have a limited duplication potential and life span, but this can be bypassed with immortalized cells. Relatedly, cell density needs to be carefully controlled based on the cell type (short vs. long doubling times) and the planned length of the assay. A major challenge in pairing cells in co-culture growth is to find a growth medium that will supply each cell type with enough nutrients to survive, but that also does not overstimulate proliferation. Allowing cell types to interact and support themselves by the mere process of adhesion and exchange of various factors is one of the more exciting aspects of this model and frequently has led to new insight in the absence of the high serum, high nutrient mediums used in isolated 2D cultures of the past. Depending on the chosen setup of cells (e.g., primary vs. immortalized) and pairing of the cell types (e.g., endothelial and pericytes, stroma and (tumor) parenchyma, same species or mouse/rat vs. human) interpretation and generalization of the results must take these outsets in consideration.

### 10.4 Concluding remarks

In summary, the EC spheroid assay is an in vitro and possibly an in vitro*/*in vivo co-culture method assisting in the comprehension of initial stages of angiogenesis and angiogenesis-associated processes in a continuum—from initiation to development and progression. The system allows for monitoring tissue development as well as testing pro- and anti-angiogenesis targeted treatments with and without the combination of chemo/radiation therapy in a more relevant microenvironment than traditional 2D cultures. Thus, this in vitro*/*in vivo preclinical cell and animal model holds promise to not only enable the identification of authentic and novel biomarkers but also provide enhanced predictive utility for drug development and discovery.

## 11 Endothelial cell metabolism

Metabolism is a biochemical reaction network that catabolizes nutrients into metabolic products such as energy and biomass that are needed to survive, proliferate, and adapt to environmental challenges. As such, they provide a functional readout of the observed phenotypic changes. Metabolomics is the systematic study of the aforementioned metabolic products and is currently enjoying a revival (“Metabolomics, the apogee of the omics trilogy” [167]). The ultimate goal of metabolomics is to understand: (1) how cells under different circumstances apply and reroute their metabolic pathways, and (2) what are the required start and end products in relation to the observed phenotype.

The majority of EC remains quiescent for years [168], but upon exposure to pro-angiogenic stimuli (such as VEGF), they can rapidly switch to a proliferative and migratory state in health and disease. At the base of this switch lies a tightly regulated metabolic network, providing the necessary energy and building blocks for EC to respond accordingly [169, 170]. ECs rewire their metabolism in a highly specific manner to optimally execute these functions [171]. Indeed, ECs are highly glycolytic, producing large amounts of ATP and lactate even in the presence of ample oxygen [170].

In the following section, we will discuss three approaches that have been established to monitor metabolic activities of EC: (1) tracer metabolomics, (2) radioactive tracer-based metabolic assays, and (3) measurements of extracellular acidification rate and mitochondrial respiration by the Seahorse XF analyzer. These approaches are complementary, and it is recommended to perform these assays in parallel for a more complete metabolic phenotyping.

### 11.1 Tracer metabolomics

The interpretation of metabolic activities based on the (relative) abundances of metabolites under specific conditions remains a formidable challenge, largely because the network of biochemical reactions is highly interconnected. For instance, an increase of intracellular serine levels can be due to increased uptake but also by de novo synthesis using 3-phosphoglycerate from glycolysis as a substrate. As such, the majority of metabolites can be linked to multiple sources and by merely measuring the (relative) abundance of metabolites, one cannot readily deduct the responsible pathways and their active interplay. This knowledge is paramount to understanding cellular biology. A specialized field in metabolomics, Tracer Metabolomics [172], tackles this challenge and uses labeled non-radioactive substrates (carrying ^13^C, ^15^N, ^18^O, ^2^H, etc.) to monitor the distribution of the labeled isotopes throughout the metabolic network and as such, provides the actual contribution of nutrients to specific metabolites and consequently the relative activity of metabolic pathways.

### 11.2 Experimental tracer metabolomics setup of EC

A possible cell culture format for EC is performed using a 6-well plate, in which at least 150,000 ECs are plated. ECs need to be grown in customized medium of which the substrate of choice is withdrawn and replaced by its non-radioactive isotopically labeled counterpart (tracer). The majority of tracers are nutrients that are taken up by the cell (for instance, glucose, glutamine, palmitic acid, etc.), of which one of the constituent atom species (^12^C, ^14^N, ^1^H, ^16^O, etc.) is replaced by its non-radioactive isotopologue (respectively, ^13^C, ^15^N, ^2^H, ^18^O, etc.). ECs take these labeled nutrients up and process them similarly compared to their unlabeled form. Consequently, all of the downstream metabolites and metabolic pathways linked to the specific tracer will incorporate the labeled atoms. It is critical that ECs reach isotopic steady state, a condition that is accomplished when the labeled isotope enrichment in a given metabolite is stable over time relative to the experimental error [172]. Practically, steady state in EC is reached in 24–48 h using, respectively, glucose or glutamine tracers [173, 174], for palmitic acid up to 72 h of incubation is required [169]. The difference in labeling time necessary to reach isotopic steady state depends on both the fluxes (i.e., rate of conversion) from the nutrient to the metabolite of interest, and the concentration of that metabolite and all intermediate metabolites [172]. The fractional contribution, which is the fraction of a metabolite’s carbon (or other atom) produced from a certain nutrient, in the downstream metabolites as well as their isotopic envelopes (isotopologues) can be quantified using mass spectrometry (MS). Prior to MS analysis, ECs are washed using a physiological (0.9%) NaCl solution and metabolites are extracted in an organic (50% methanol–30% acetonitrile–20% H_2_O) extraction buffer. It is of utmost importance that the extraction is carried out swiftly because a cell’s metabolism changes within the order of seconds to minutes [175]. Extracts are centrifuged to remove the precipitated proteins and insolubilities. The supernatant, containing the polar metabolites, is loaded onto the MS platform.

### 11.3 Mass spectrometry

The instrument of choice for carrying out (tracer) metabolomics is mass spectrometry (MS), mainly due to its superior sensitivity and speed of analysis in comparison with other technologies such as nuclear magnetic resonance (NMR) spectroscopy, as well as its flexibility in applying different kinds of chromatography (liquid or gas chromatography, LC, or GC) to study different chemical classes of metabolites [176]. We will only focus on the analysis of polar metabolites (amino acids, organic acids, hexose phosphates, etc.). Apolar species such as lipids are beyond the scope of this section. A crucial requirement for the MS in tracer metabolomics relates to its resolution, the ability to accurately quantify the isotopologue profiles of the metabolites of interest. As such, the current benchtop LC–MS OrbiT-RAPs are among the instruments of choice to conduct tracer metabolomics. Alternatively, GC-linked (triple) quadrupole MS can be used as well. For the separation of polar metabolites prior to the MS, two main types of chromatography can be applied: (1) hydrophilic liquid chromatography (HILIC) [177] and (2) ion-pairing chromatography [178]. The latter has superior binding and separation capacities compared to HILIC, but it restricts any further usage of the chromatograph to ion-pairing mode. In this perspective, HILIC provides a better solution, despite the lesser separation capacity compared to the ion-pairing setup as it comes without any restrictions to apply different chromatography settings on the same platform.

### 11.4 Data processing

Data extraction of isotopic profiles occurs via vendor-specific software or *in*-*house* developed software platforms (e.g., BIOMEX™) [179]. In general, the workflow starts with *in*-*house* libraries containing information on the exact mass of the target molecule and its retention time during the chromatographic separation to identify the metabolite of interest. Next, the abundances of the isotopes of different metabolites of interest are quantified. Following a correction for natural abundances [172, 180] to facilitate proper interpretation of the labeling data, the fractional contribution is calculated [172].

### 11.5 Radioactive tracer-based assays

**Fig. 9:**
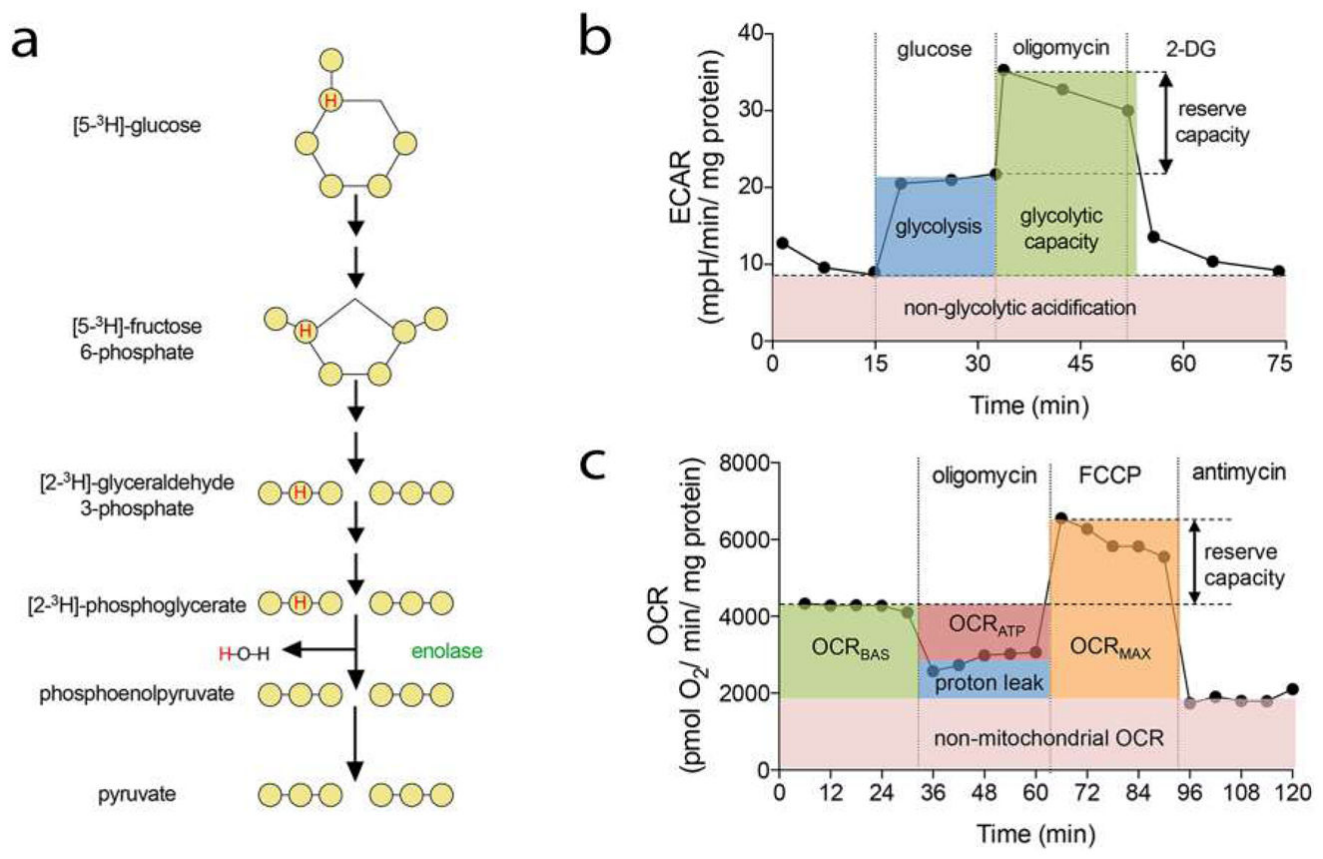
Methods to measure EC metabolism using radioactive tracers and Seahorse XF analyzer. **a** Schematic representation of glycolytic flux measurements with [5–^3^H]-glucose. A single tritium present on 5C glucose is released as water in the ninth step of glycolysis catalyzed by enolase. **b** Schematic representation of the modified Glycolysis Stress Test. The first measurements of ECAR are performed while ECs are incubated in glycolysis stress test medium (without glucose and pyruvate). The injection of glucose leads to the saturation of glucose concentration and allows measuring glycolytic rate (blue). Second, injection of oligomycin blocks oxidative ATP production and shifts the energy production to glycolysis, with the subsequent increase in ECAR revealing the cellular maximum glycolytic capacity (green). The difference between glycolytic capacity and glycolysis rate defines glycolytic reserve. The final injection of 2-deoxyl-glucose (2-DG) inhibits glycolysis, and the resulting decrease in ECAR confirms that the ECAR produced in the experiment is due to glycolysis. ECAR, prior to glucose injection or after 2-DG injection, is referred to as non-glycolytic acidification (pink). **c** Schematic representation of the modified Seahorse Cell Mito Stress Test. First injection of oligomycin blocks ATP synthase and allows the calculation of the ATP coupled oxygen consumption rate (OCR_ATP_; red). Second, injection of FCCP maximizes the OCR by uncoupling the OXPHOS, enabling to calculate the spare respiration (reserve capacity). Third, antimycin-A treatment blocks complex III of ETC enables the calculation of the basal mitochondrial respiration (OCR_BAS_; green), the maximal mitochondrial OCR (OCR_MAX_; orange), the proton leakage (blue), and the non-mitochondrial OCR (pink)

As introduced above, unlabeled or labeled metabolites can be quantified by MS or NMR. However, these techniques display some limitations, as they do not quantify the absolute flux, which reflects the rate of turnover of molecules through a metabolic pathway. Radioactive isotope labeling is a widely used experimental approach to analyze metabolic flux. This technique is based on the principle that nutrients labeled with radioactive tracers (e.g., ^3^H and ^14^C) at specific positions within the molecule release the radioactive label as ^3^H_2_O or 14CO_2_, respectively, upon specific enzymatic activities. Consequently, the generated ^3^H_2_O and 14CO_2_ diffuse into the medium and can be measured by trapping its release on soaked filter paper and subsequently quantified by scintillation counting [170]. As an example, the use of [5-^3^H]-glucose enables to quantify glycolytic flux [181] because the single tritium at the C5 of glucose is removed by a condensation reaction catalyzed by enolase (Fig. 9a), hence releasing radioactive ^3^H_2_O in the medium. Similarly, by using [9,10–^3^H]-palmitic acid, one can measure the radioactive ^3^H_2_O formed during fatty acid oxidation [169].

In order to map the different metabolic fates of glucose and other crucial substrates, a set of differently radioactive-labeled tracers are needed to identify and quantify the ongoing biochemistry. As an example, in parallel with the glycolytic flux measured by [5–^3^H]-glucose, the downstream oxidation of glucose can be monitored by quantifying the release of ^14^CO_2_ by [6–^14^C] glucose or uniformly labeled [^14^C_6_] glucose. Notably, by using [6–14C] glucose, information on the oxidation of glucose in the tricarboxylic acid (TCA) cycle is obtained, whereas the [^14^C_6_] glucose tracer generates ^14^CO_2_ via two different metabolic pathways: the oxidative pentose phosphate pathway (oxPPP) and the TCA. Also, the carbon at position 1 in glucose is liberated in the oxPPP. Hence, using [1–^14^C] glucose in parallel with [6–^14^C] glucose, the oxPPP flux can be estimated by subtracting the rate of [6–^14^C] glucose from the one of [1–^14^C] glucose. In summary, assessing the choice of the tracer and its specific labeling positions is essential in terms of the biological question that needs to be addressed. Similar approaches are available to investigate other “non-glucose” related fluxes, for example by using [^14^C_5_] labeled glutamine to determine glutamine oxidation.

### 11.6 Measurements of extracellular acidification rate and mitochondrial respiration using a Seahorse XF analyzer

The Seahorse extracellular flux (XF) analyzer enables to assess different aspects of cell metabolism. Apart from providing an indirect readout of anaerobic glycolysis through measurements of the extracellular acidification rate (ECAR) [182], it can also assess oxidative phosphorylation (OXPHOS) (through oxygen consumption rate (OCR)). A commonly used method to determine glycolysis by the Seahorse XF analyzer is the measurement of acidification of the medium (pH changes) in a glycolysis stress test [183]. In this technique, glucose, oligomycin (inhibitor of ATP synthase in the electron transport chain (ETC)), and 2-deoxy-glucose (2-DG, inhibitor of hexokinase) are sequentially administered while measurements of ECAR are being performed. In the first phase of the experiment, ECs are cultured in medium deprived of glucose or pyruvate (the glycolysis stress medium). Supplementation of glucose feeds glycolysis, which allows estimation of glycolytic rate by calculating the difference between ECAR before and after addition of glucose. ECAR, prior to glucose injection, is referred to as non-glycolytic acidification resulting from other sources of extracellular acidification not attributed to glycolysis (Fig. 9b). Next, oligomycin, which inhibits mitochondrial ATP production, lowers the ATP/ADP ratio, and shifts energy production to glycolysis, is supplemented to measure the maximum glycolytic capacity. The difference between ECAR before and after oligomycin supplementation is a measure of the glycolytic reserve capacity. The last injection of 2-deoxy-glucose (2-DG) inhibits glycolysis, and the resulting decrease in ECAR confirms that the ECAR produced in the experiment is due to glycolysis. Moreover, inhibition of glycolysis by 2-DG also provides information on non-glycolytic ECAR of cells (Fig. 9b).

Mitochondrial respiration can be assessed by using a modified Seahorse Cell Mito Stress Test [184]. This test sequentially uses modulators of respiration that target different components of the ETC. Oligomycin, carbonyl cyanide 4-(trifluoromethoxy) phenyl-hydrazone (maximizing OCR by uncoupling the OXPHOS), and antimycin (blocking OXPHOS by inhibiting complex III) are sequentially injected to determine basal mitochondrial OCR (OCR_BAS_), ATP-dependent OCR (OCR_ATP_), maximal respiration (OCR_MAX_) and non-mitochondrial respiration (OCR_non-mito_), proton leak, and spare respiration (Fig. 9c) [184].

### 11.7 Limitations and challenges, problems and pitfalls in probing EC metabolism

Tracer Metabolomics provides information on relative pathway activities, qualitative changes in pathway contributions via alternative metabolic routes, and nutrient contribution to the production of different metabolites. In this perspective, a limitation of tracer metabolomics is the lack of quantitatively estimating metabolic fluxes, which provide additional quantitative and complementary information such as pathway directionality. Nonetheless, resolving metabolic fluxes is time and data intensive [185] and often requires applying computational modeling of the data. A challenge for tracer analyses is the requirement of expert knowledge at the level of mass spectrometry and biochemistry. This relates to the choice of specific tracers needed to answer biological questions as well as the tools (chromatography, mass spectrometry, software) available to extract and process the required data. The optimal cell culture experiments apply customized medium of which the unlabeled form of the tracer has been withdrawn. This setup maximizes the amount of label entering the cell and increases the likelihood to identify and quantify the labeled “downstream” targets. A pitfall occurs when the unlabeled form cannot be withdrawn; in this case, the labeled compound can be added in equimolar quantities to the medium as the unlabeled compound. This poses potential problems: (1) The amount of label incorporated in downstream metabolites is reduced and pathways using low amounts of the tracer might be overlooked; and (2) doubling the concentration of a specific substrate can induce metabolic changes into the cellular biochemistry, for instance doubling the glucose levels from the physiological 5.5–11 mM places EC in a diabetic-like “high glucose” environment, triggering additional metabolic changes or, in worst case, inducing toxicity.

Not all of the metabolites present in the cell are detectable by MS, the major causes for this relate to the abundance and/or intrinsic technical challenges, such as the ionization potential of the metabolite of interest. To tackle this challenge and still gain information on this missing piece of the puzzle, one needs to look at the fractional contribution and isotopologue profiles of neighboring metabolites that reflect the labeling pattern of the missing component, for instance the lack of oxaloacetate detection can be accounted for by looking at the isotopologue profile of aspartic acid. Consequently, engaging on tracer analyses implies that sufficient metabolites need to be taken into account ensuring full coverage of the pathway(s) of interest.

#### General limitations of (radioactive) tracer-based assays

Cell metabolism is a very dynamic process, and the metabolic needs as well as metabolic activity of EC have been shown to correlate with the cell cycle phases [171, 186]. Therefore, one should carefully control cell density while performing these experiments. Moreover, the results and interpretation of metabolic studies using radioactive tracers depend on appropriate normalization of the data, as ineffective or poorly chosen normalization methods can lead to erroneous conclusions. Therefore, normalization to total protein concentration, cell number, or DNA content is considered as the golden standard in metabolomics.

#### Seahorse XF analyzer

Although assays performed on a Seahorse XF analyzer requires only small number of cells (30,000 cells/well in 24-well assay plate format), they still present several limitations that one should take into consideration while performing these experiments. Additional metabolic processes, like CO_2_ generated during tricarboxylic acid (TCA) cycle activity, can change the pH of the media and interfere with the readout and interpretation of the results. Since ECAR is essentially a measurement of pH, buffering agents (e.g., sodium bicarbonate) are not included in the assay medium. Moreover, as bicarbonate and media pH play a role in regulating glycolysis, they can influence and confound measurements of ECAR. In order to increase the accuracy of the performed experiments, application or pre-treatment with additional chemical inhibitors (e.g., AR-15585; lactate export blockers) can be of use to determine whether changes in pH come from lactate excretion or other sources of media acidification. Optionally, glutamine can be also withdrawn from the media, in order to assess ECAR coming from glycolytic pathway and not from glutaminolysis [187] albeit the removal of specific nutrients can also induce off-target metabolic changes. In addition, depending on the used cell type, the concentration of each chemical compound must be carefully determined, in order to obtain the optimal result for each of these measurements. For instance, it has been observed that an overdose of oligomycin can lead to maximal inhibition of OCR, which can result in a progressive increase of OCR over time [184]. Unfortunately, the molecular mechanism underlying this observation remains elusive.

## 12 Endothelial cell precursors

Stem and progenitor cells provide a necessary homeostatic source of tissue-specific mature cellular elements that permit appropriate cell functions through replacement of injured, diseased, and senescent cells in many organ systems throughout the life span [188]. While the identity, biology, and molecular regulation of hematopoietic-, intestinal-, skin-, and skeletal muscle stem and progenitor cells are well recognized, little is known of the organization of the reparative cells that comprise the vascular system. In 1997, Asa-hara and colleagues [189] reported on the identification of circulating progenitor cells for the endothelial lineage. Subsequent studies have clarified that those putative “endothelial progenitor cells” (EPC) did not possess the capacity to undergo a stable lineage switch to the endothelium, but were comprised of numerous hematopoietic cells that can serve paracrine pro-angiogenic functions to promote vascular repair and replacement but are incapable of integrating as *a bona fide* EC in the injured vasculature [190]. In fact, these pro-angiogenic cells can upregulate “endothelial cell” markers and thus give the impression they were becoming EC at sites of injury, but failed to persist as functional vasculature long term. A study by the Mayr lab later showed that these are in fact mononuclear cells and that the “endothelial” surface markers were acquired by a process of membrane fusion with platelet microparticles [191]. Only endothelial colony-forming cells (ECFC), also called late outgrowth or blood outgrowth EC (BOEC), are direct EC precursors that form vessels in vivo [190]. This paragraph will briefly discuss available evidence for and remaining controversies regarding the evaluation of endothelial stem and progenitor cells in mouse and man.

### 12.1 Assays to identify endothelial stem and progenitor cells

A simple working definition of a stem cell is a clonal, self-renewing cell, which gives rise to differentiated cell types [188]. A somatic stem cell may be multi-potent (giving rise to multiple types of differentiated cells) or unipotent (differentiating into a single lineage of mature cells). A progenitor cell displays clonal proliferative potential, but progenitor cells lack self-renewal potential. However, progenitor cells can expand into a single lineage of differentiated cells. Multiple assays have been developed to define stem and progenitor cells that reside in different organs and tissues [188]. Stem cells in any tissue must be interrogated for clonal self-renewal (most stringently proven by long-term in vivo persistence with retained capacity for contribution to differentiated progeny [fate mapping approach] and/ or use of transplantation assays into primary and secondary recipient hosts). Progenitor cells are often measured with colony-forming assays in vitro (to prove a precursor product relationship). Stem and progenitor cells may also display distinguishing cell surface markers that can be used to prospectively isolate the cells through the use of monoclonal antibodies and flow cytometry or magnetic bead separation. Most recently, stem and progenitor cells from some tissues have become definable by single-cell gene expression technologies to permit identification by a unique molecular signature [192, 193]. Presentation of some selected publications (as examples) identifying putative stem and progenitor cells for the vascular system (using many of the above criteria) follows below.

#### Progenitor cell assays

To identify the progenitor cells for the endothelial lineage, we need to decide what criteria should be used to define cells belonging to the endothelial lineage. Other paragraphs in this paper have made arguments as to the “best” assays used to define EC characteristics and their functions based on published evidence. Obviously, there are numerous phenotypic, morphological, physiological, genomic, proteomic and functional parameters that constitute unique and characteristic behaviors of EC. However, one cannot identify cells of the endothelial lineage by expression of a few cell surface markers or of a few RNA transcripts. Several selected works that use comprehensive definitions for identifying progenitor cells for the endothelial lineage follow.

Patel et al. [194] recently proposed several features that can be used to define resident vascular endothelial progenitor cells for the murine vasculature. Based upon the level of expression of vascular endothelial cadherin (VE-cadherin/CD144), platelet EC adhesion molecule (PECAM-1/CD31), VEGFR-2, and leukocyte common antigen (CD45), ECs present in blood vessels in induced healing wounds or growing in response to implanted tumors were identified as endovascular progenitor cells (EVP), transit amplifying cells (TA), and definitive differentiated cells (D). Evidence for these distinct populations displaying different functional states was presented using clonogenic in vitro analysis, fate mapping studies in vivo, transplantation analysis, variations in gene expression, differences in phenotypic analysis (flow cytometry and immunofluorescence), and the requirement of certain transcription factors for the differentiation of EVP to transient amplifying and definitive differentiated states.

Evidence for the identification of human endothelial progenitor cells (circulating or resident) has been published by several groups. Analysis of the circulating blood of patients following a sex-mismatched bone marrow transplant indicated that some of the BOEC were derived from the host, but the most proliferative cells were donor bone marrow derived [195]. Umbilical cord blood was reported to be enriched for clonogenic ECFC that displayed a hierarchy of proliferative potential and in vivo vessel formation compared to adult peripheral blood [196]. Furthermore, resident vascular EC derived from umbilical veins or human abdominal aorta contained clonogenic ECFC with vasculogenic potential. Very recently, human-induced pluripotent stem cell (hiPSC)-derived ECFC have been reported with properties that are similar to umbilical cord blood ECFC but with some distinct differences in gene expression [197].

#### Vascular endothelial stem cells

Fang et al. [198] reported that stem cells for the endothelial lineage can be isolated from lung blood vasculature. Vascular endothelial stem cells (VESC) expressing the phenotype CD31^+^CD105^+^Sca-1^+^CD117^+^ (devoid of any mature lineage markers for the hematopoietic system) can be isolated from collagenase-digested lung tissue. The VESC displayed clonogenic colony-forming activity in vitro and when implanted in Matrigel subcutaneously in mice, gave rise to donor-derived blood vessels. Indeed, when 15 VESC (green fluorescent protein tagged: GFP) were implanted with B16 tumor tissue, GFP-labeled blood vessels were identified in primary, secondary, tertiary, and quaternary tumor implants. Even a single VESC displayed the capacity to form donor blood vessels in vivo in a Matrigel implant. While some flow cytometric estimations of the frequency of the CD117 expressing VESC in different tissues were reported, no detailed visualization of the location of the VESC in arteries, veins, and capillaries in different tissues was presented. In addition, no fate mapping studies to identify the contributions of the putative VESC within various vascular beds in homeostatic endothelial turnover were reported.

Naito et al. [199] used the Hoechst staining method to identify resident vascular EC in the side population that were dormant in steady state, but possessed clonal colony-forming activity, produced large numbers of mature endothelial progeny, and when transplanted into ischemic lesions, restored blood flow and reconstituted de novo blood vessels at the site of injection. Although the surface markers of the SP cells were similar to primary capillary EC, the gene expression pattern of the SP and main population that fail to retain the Hoechst dye was significantly different. Several unique cell surface markers were identified in the SP cells that may permit prospective isolation of these putative VESC. Use of Hoechst method fails to permit identification of the putative VESC in the organ and tissue vasculature during development or after injury and will rely upon further studies using novel cell surface markers.

More recently, Yu et al. [200] identified protein C receptor-expressing EC as VESC in the mammary fat pad, skin, and retina. These VESC exhibited robust clonal expansion in tissue culture, high vessel reconstituting ability upon multiple rounds of transplantation, and long-term clonal expansion in lineage tracing experiments. Indeed, the VESC were determined to be bipotent, with contributions not only to the endothelium but also to pericytes throughout vessels in multiple tissues. The authors suggested that VESC underwent endothelial to mesenchymal transition to become pericytes in the examined vascular beds [200].

### 12.2 Limitations and challenges

Most of the > 9564 publications identified by the term EPC in Pubmed (endothelial progenitor cell, Pubmed, July 30, 2017) have failed to provide sufficient evidence that the putative EPC under investigation display clonal proliferative potential and/or the capacity to directly form EC that undergo vasculogenesis in vivo to form new vessels or integrate long term into injured vessels in vivo as EC. If the EPC in question cannot directly give rise to cells of the endothelial lineage at a clonal level (in vitro or in vivo) or function as a bona fide EC in vivo, then the term EPC should not be applied to those cells [190]. The work of Patel et al. [201] has shown that endothelial progenitors can be identified by applying stringent criteria. However, several issues remain to be addressed such as the specific sites, localizations and contributions of EVP, transient amplifying and definitive differentiated cells localization in organs and tissues at homeostasis (artery, vein, or capillary bed), the contributions of EVP to transient amplifying and definitive differentiated cells during homeostasis, differences in the EVP among different organs across the life span of the mouse, and, last but not least, determination of whether the EVP represents an endothelial stem cell. Human EPC have been identified [195–197]; however, no unique identifying markers have permitted prospective isolation of ECFC from circulating blood or blood vascular endothelium to permit identification of the site of origin of ECFC in human subjects and determination of whether these cells display stem cell activity for the endothelial lineage. Several papers have published evidence for the presence of resident VESC in mice; however, the relationship between the unipotent VESC [198, 199] and the bipotent VESC identified by Yu and colleagues [200] remains unclear. Identification of unique and distinguishing characteristics of the VESC that discriminate these stem cells from progenitor and mature endothelial elements awaits further analysis. The difficulty in identifying proper VESC begs the question what the homeostatic function of these stem cells would be in tissues composed of differentiated endothelial and other vascular cells that retain a highly regenerative capacity.

### 12.3 Concluding remarks

Altogether these data suggest that progress in identifying stem and progenitor cells for the vascular endothelium is possible when using multi-parameter evidence that the putative precursor cells are irreversibly differentiating into cells of the endothelial lineage. The use of multi-parameter analytics will be required to permit novel studies on the biology of vascular endothelial stem and progenitor cells and likely may alter our understanding of vascular development and the pathophysiology of vascular diseases.

## 13 Microfluidic assays

The use of microfluidic cell culture systems has changed the way in which we study and manipulate living cells, and numerous researchers have leveraged the capabilities of these systems to advance and refine our understanding of angiogenesis and microvascular function. Cutting-edge microfluidic cell culture models for studying angiogenesis have successfully incorporated principles from quantitative analyses of vascular function, in vitro flow chambers, microfabrication techniques, and 3D tissue scaffolds. Consequently, microfluidic approaches have enabled unprecedented levels of control of chemical gradients, fluid flow, matrix composition, and cell–cell interactions, all of which can be integrated to provide a physiologically relevant context for studying angiogenesis.

### 13.1 Development and capabilities

**Fig. 10:**
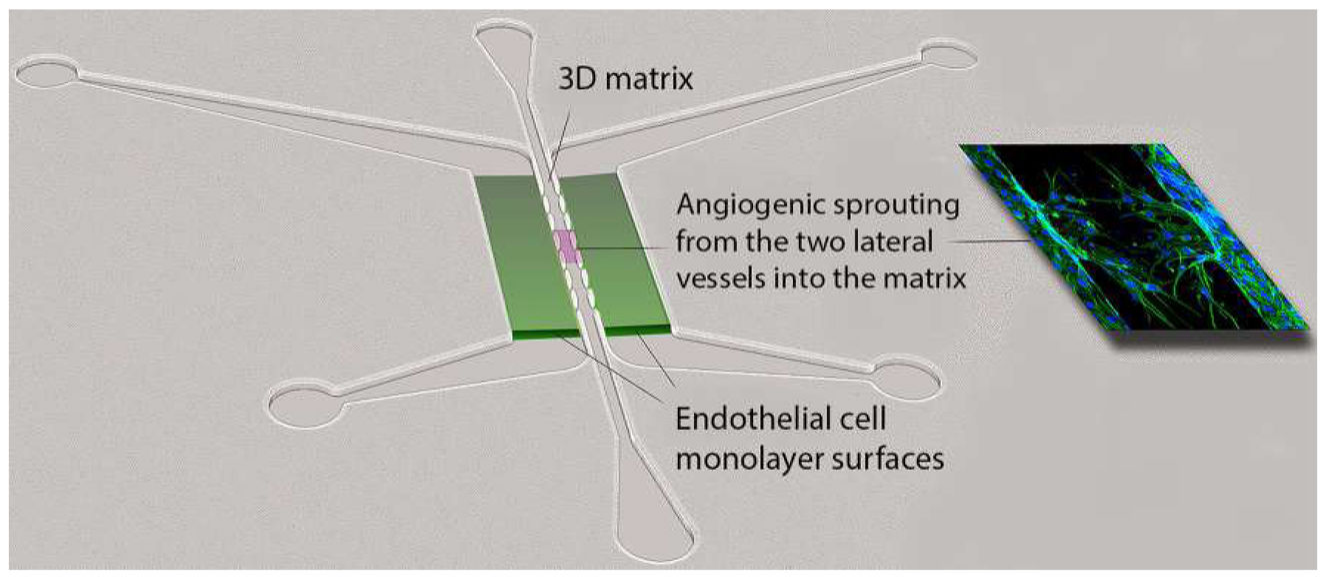
PDMS microfluidic device for analyzing angiogenesis. Fluid flow can be controlled connecting syringe pumps to the ports, or by imposing hydrostatic gradients. Flow can be directed through the endothelial lumens (green), or across the endothelial junctions, through the central matrix gel. Sprouting occurs through the apertures that flank the central 3D matrix and is easily visualized and quantified (Adapted from [221])

Recent advances in microfabrication and biological integration have helped to propel the design and implementation of microfluidic systems, which comprise an emerging class of highly modular in vitro culture models of the microcirculation [202, 203]. Microfabricated devices can provide cultured cells with a microenvironment similar to that in vivo, including the correct ECM (ECM) composition, chemical species, associations with other cells, and mechanical signals to mimic tissue- and organ-level function [204]. Interestingly, microfluidics allows generating chemical gradients and enables the study of EC chemotaxis in 3D geometries. These systems also contain networks of micron-scale fluid-filled channels that are similar in size and architecture to microvessels in vivo [205, 206] to faithfully reproduce certain microvascular phenomena in vitro. Consequently, the application of microfluidics for studying angiogenesis has emerged as a major research thrust at the interface of microsystems engineering and biomedicine. At present, the majority of microfluidic systems are constructed by soft lithography or replica molding of an elastomeric and biocompatible polymer (polydimethylsiloxane or PDMS) [207]. This process is well suited to create structures with defined shapes on the micrometer scale that can be used to position cells and tissues, control cell shape and function, and create highly structured 3D culture microenvironments [208–210]. Researchers have used this capability to assess the influence of the host tissue environment and heterotypic cell–cell interactions on angiogenesis [211] by co-culturing vascular EC organized as an intact vessel structure with other cell types such as cancer cells [212–215], stromal fibroblasts [216, 217], vascular smooth muscle cells [218], and bone marrow cells [219]. Applications for these microfluidic co-culture studies include fundamental studies of cell population behaviors, high-throughput drug screening, and tissue engineering [211]. Application of microfluidic systems has also contributed significantly to our understanding of flow-mediated angiogenesis (Fig. 10). For instance, multiple studies have shown that interstitial flow potently induces angiogenesis [220–222] and more recently lymphangiogenesis [223, 224]. These studies have also shown that the vessel sprouts triggered by interstitial flow preferentially form against the direction of flow [220–223]. In other words, vessel sprouts tend to originate from a local pressure minimum and seek the higher pressure vessel or source [221]. Interestingly, this same sprouting behavior was recently observed in vivo in avian embryos [225]. In addition to interstitial flow, intravascular shear stress has been shown to control sprouting angiogenesis [221, 226] and anastomosis of vessel sprouts [227]. Collectively, these findings suggest that the local fluid mechanical environment can both determine whether sprouting occurs and specify where these sprouts originate from the parent vessel. Moreover, the ability of microfluidic system to apply specific levels of fluid mechanical stimuli in a highly controlled environment will be invaluable toward advancing the mechanobiology of angiogenesis [228].

One limitation to PDMS replica molding is that this process produces microchannels and subsequent endothelial-lined vessel structures with rectangular cross sections. To address this limitation, alternative fabrication techniques have been used to form engineered microvessels with circular cross sections. The most widely used technique to fabricate this type of microchannel is to cast a 3D scaffold housed within a PDMS chamber around a cylindrical needle or rod of approx. 100 μm in diameter [229]. Once the scaffold has polymerized, the needle or rod cast is removed leaving an open cylindrical microchannel embedded within the 3D scaffold. Scaffold-embedded circular microchannels have been used to monitor focal leaks in the endothelial-lined microchannels, by adopting imaging techniques of fluorescent tracer dyes conjugated to macromolecules (e.g., albumin or high-molecular weight dextran) that are widely used in intravital microscopy techniques for measuring vascular permeability in vivo [230, 231]. In addition to quantifying vascular barrier function, circular microchannels have been used to measure vessel sprouting and anastomosis [232].

Since the fabrication of circular microchannels employs a non-lithographic technique, it loses some of the desirable attributes achieved with soft lithography such as rapid prototyping and scalability for high throughput [205]. In addition, circular microchannels require casting and removal of a cylindrical needle or rod inside a hydrogel; thus, they are constrained to simple linear structures [233]. To address the aforementioned limitations of the cylindrically casted microchannels, another circular lumen fabrication technique was recently developed that uses the principle of viscous finger patterning of pre-polymerized hydrogel specified inside PDMS microchannels of different geometries, including branched networks that mimic the topology of bifurcating microvessels [234]. Another hydrogel-embedded microfluidic model that is of significance uses micropatterned stencils to produce microvascular network inside natural 3D hydrogels such as collagen and alginate [235, 236]. Finally, the recent advent of 3D printed microfluidic vessel networks [237] have enabled the patterning of more complex branching networks that mimic in vivo physiology [238].

### 13.2 Advantages of microfluidics for angiogenesis studies

The use of a chip supported by a microfluidic system for EC culture represents specific advantages. Compared to in vivo studies, these systems are relatively straightforward to apply controlled perturbations of extrinsic cues, such as fluid shear stress and biomolecular gradients. Furthermore, in contrast to conventional in vitro angiogenesis assays, vessel structures formed in microfluidic systems can be readily perfused to evaluate the effects of fluid flow on vessel maturation. The feature of these systems enables the visualization and the precise quantification of vessel function (e.g., MVD and vascular permeability) in response to various extrinsic cues and allows the construction of distinct tissue compartments (e.g., vascular and perivascular). Moreover, the cells that comprise each tissue compartment can be independently genetically modified to reproduce conditional knockout studies in vitro. Interestingly, the material that is typically used for microfluidic devices (PDMS) is fairly inexpensive and enables rapid prototyping and the total volume of biological reagents (e.g., cells, ECM, and culture media) that is required for microfluidic experiments is very low and on the order of microliters. Finally, these devices can be further implemented with optical sensors for analytical measurements. Altogether these features enable in vitro screening platforms for the efficacy of candidate drug compounds in a physiologically relevant setting [239, 240].

### 13.3 Limitations and challenges

Microfluidic technologies typically require specialized fabrication techniques and make use of challenging complex 3D geometries that require multilayer construction. These technologies are beyond the research expertise of most biology laboratories. Moreover, despite the beneficial properties of PDMS (e.g., biocompatible, elastomeric, optically transparent, gas and water permeable), there are also several important limitations such as leaking of non-crosslinked oligomers and non-specific absorption of hydrophobic molecules that can critically affect cell-signaling dynamics [241, 242].

### 13.4 Concluding remarks

Existing and emerging technologies for casting, molding, or 3D printing are changing the way we study cells in vitro. These methods bridge the gap between tissue culture dishes and animal models, allowing more informative and representative co-culture systems. Future development may allow recapitulation of additional aspects of vascular biology in vitro such as intussusceptive splitting or network remodeling.

## 14 Flow cytometry and cell sorting assays

Cancer cells modify the microenvironment at the primary site to favor tumor growth and dissemination [243, 244]. Similarly, tumor cells induce systemic alterations and modify the microenvironment at the (prospective) metastatic site to create a (pre) metastatic niche favorable for tumor cell seeding, survival, and growth [245]. The tumor microenvironment (TME) comprises many different cell types, in particular blood and lymphatic endothelial cells, carcinoma-associated fibroblasts (CAF), myeloid and lymphoid inflammatory and immune cells [243, 244]. Tumor angiogenesis promotes local tumor growth and invasion by providing oxygen and nutrients, cytokines and growth factors (angiocrine effects), as well as an escape route toward metastasis [246]. In response to tumor-produced factors, recruited CD11b^+^ myeloid cells polarize toward an alternative (M2) activation state, in contrast to the classical activation state (M1) [247, 248]. M2-polarized CD11b^+^ cells promote tumor growth, invasion, metastasis, and angiogenesis through the release of growth and motility factors, including VEGFs, FGFs, tumor necrosis factor (TNF), platelet-derived growth factor (PDGF), and chemokines [247, 249, 250]. Diverse tumor-promoting pro-angiogenic CD11b^+^ cell types have been reported, including VEGFR-1^+^CD11b^+^ [251], Gr1^+^CD11b^+^ myeloid-derived suppressor cells (MDSC) [252], Tie-2-expressing CD11b^+^ monocytes (TEM) [253], and cKit^+^CD11b^+^ cells [254].

Traditionally, the TME is analyzed by immunohistological staining of tissue sections. While histological techniques have the advantage to render tissue morphology, they nevertheless bear some relevant limitations: They require antibodies that work on fixed tissues; they can only detect one or a few markers simultaneously; stained cells cannot be recovered for further analysis; they are laborious, time-consuming, and unpractical for large series of samples; and they do not allow precise quantification of the number of cells and signal intensity. To circumvent these limitations, alternative techniques were adopted [255]. Flow cytometry (often also referred to as FACS—fluorescence activated cell sorting) originally developed to analyze blood-borne cells [256, 257] is such a technique, now widely used to study the cellular composition of the TME. In addition, “true” FACS allows the isolation of cells for further analysis [258].

### 14.1 Types of assays

#### Analytical flow cytometry

**Fig. 11:**
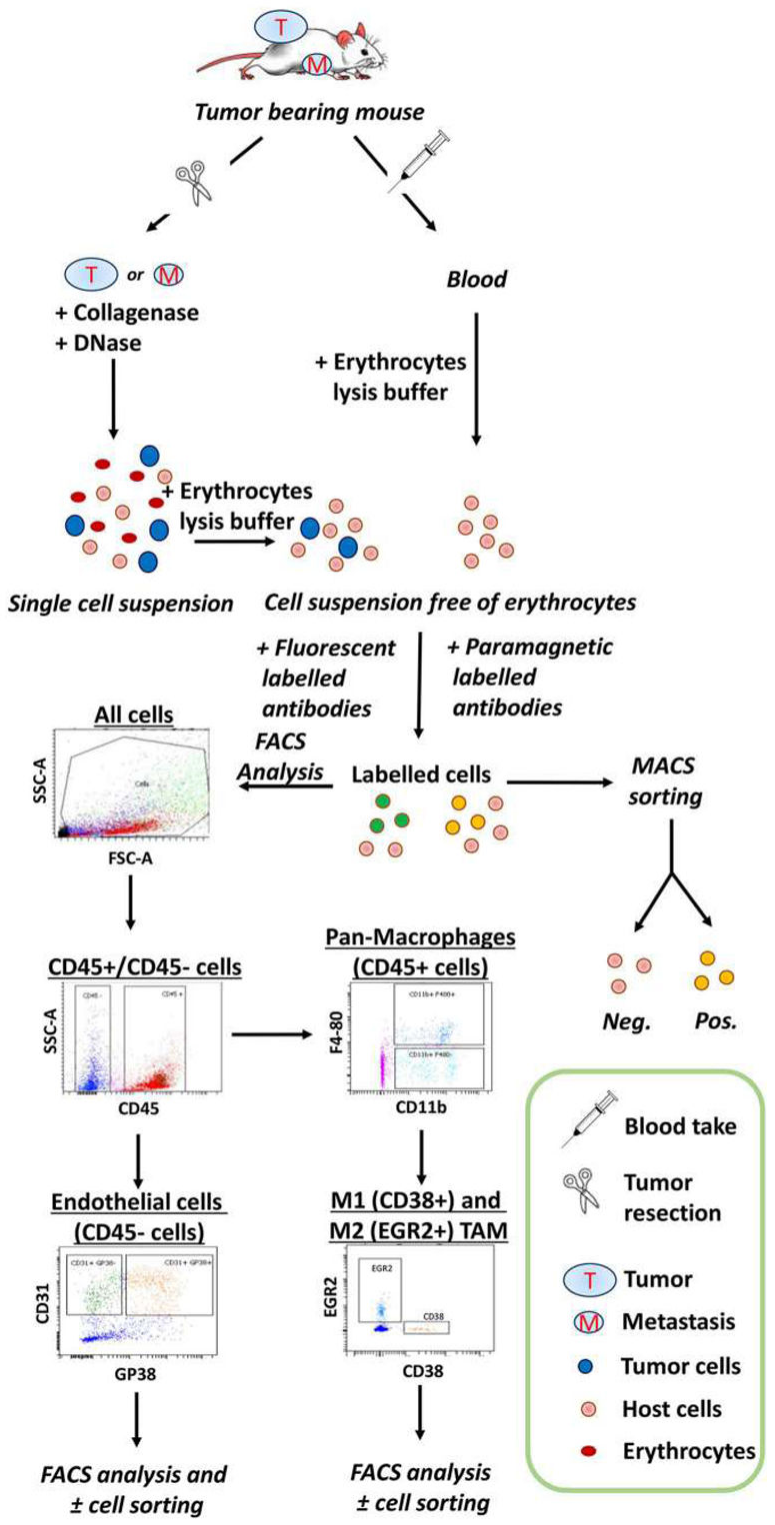
Representative flow-chart of flow cytometry and cell sorting experiments. Overview of samples collection (tumors, organs bearing metastasis, and blood) from mice and the procedure to obtain a single-cell suspension after tissue dissociation, erythrocytes lysis, and cell labeling with fluorescent or paramagnetic-coupled antibodies. Analysis of blood-circulating cells only requires a red blood cell lysis prior to incubation with antibodies of interest. Intracellular antigens can be detected by adding a cell permeabilization step. Fluorescent labeled cells are analyzed by flow cytometry and sorted by FACS while cells labeled with paramagnetic-coupled antibodies can be sorted by MACS. MACS can be used to pre-enrich cells for subsequent FACS sorting. Cell populations of interest are identified in by dot plots analysis of collected data

The critical step to analyze the cellular composition of the tumor microenvironment is to obtain a suspension of viable, single cells from the removed tumor tissue (Fig. 11). To avoid collecting cells circulating in the blood, mice are terminally perfused with physiological solution prior to tumor take. Also, care should be taken to remove the surrounding normal tissue from the tumor lesion. Harvested tumors are dissociated by collagenase and DNase treatment, and red blood cells are lysed with a lysis buffer. The cell suspension is passed through a 70-μm mesh filter to remove tissue debris and cell aggregates that can clog the FACS, followed by an incubation step with an anti-Fc receptor blocking antibody to avoid non-specific binding of relevant antibodies to immune cells. There are many commercial kits that perform well tumor dissociation and FcR blocking (e.g., from Miltenyi Biotec). Finally, cells are incubated with different antibodies for markers of cells of interest, for example, for vascular endothelial cells (CD45^−^/CD31^+^/GP38^−^), pericytes (CD45^−^/CD146^+^/CD31^−^/CD34^−^), Type 2 pro-angiogenic TAM (CD45^+^/CD11b^+^/EGR2 high/CD38 low), or MDSC (CD11b^+^, Ly6C^+^/G^+^). By using GP38 staining, it is possible to separate vascular endothelial cells (GP38−) to lymphatic endothelial cells (GP38 +). A DNA dye can be added to exclude dead cells. Analysis of blood-circulating cells only requires a red blood cell lysis prior to incubation with antibodies of interest. Cells can also be stained for apoptosis [259], DNA content (to determine cell cycle state), for the expression of intracellular proteins such as kinases (including their phosphorylation state) [260], cytokines, and pro-angiogenic factors [261] by adding a permeabilization step during the staining procedure.

The advantages of FACS-based studies are manifold: They allow the analysis of multiple markers, routinely 6–10, and up to 12 or 16, depending on the machine, laser, and configurations. This permits monitoring multiple cell populations at once with limited numbers of cells (e.g., 10^5^–10^6^). FACS experiments are easy and rapid to do and can be effectively used as a screening platform to determine the effect of drugs on endothelial cells or on tumor-associated pro-angiogenic cells. By using the right strategy and markers, one can determine the percentage of most cells of the TME. For example, we used FACS to characterize the effect of VEGFR/VEGFR-2 inhibition on angiogenesis and immune/inflammatory cells [262, 263], to identify a novel pro-metastatic/angiogenic CD11b^+^cKit^+^ cell [254, 263] and to study the contribution of CD11b^+^ cells to angiogenesis and lymphangiogenesis [247, 264, 265] in mice and human. Many factors regulating angiogenesis, including soluble proteins and microvesicles, are released from the primary tumor site and can enter the systemic circulation. FACS can be used to quantify multiple circulating cytokines at once using bead arrays (e.g., BD^™^ Cytometric Bead Array) [266]. The assay takes advantage of the broad dynamic range of fluorescence detection offered by flow cytometry and the simplicity of antibody-coated beads resulting in the efficient detection of multiple cytokines at once. This assay allows analysis of smaller sample volumes and is faster compared to traditional ELISA. Cancer-released microvesicles, encompassing exosome, apoptotic bodies, and microparticles, are small cell membrane-derived vesicles expressing cellular proteins at their surface and containing DNA, RNA, miRNA and proteins, and small molecules of the cell of origin. They have gained considerable attention recently as they allow to study tumor features through a so-called liquid biopsy approach [267, 268]. FACS can be used to study microvesicles to determine their level and the surface composition [269].

#### Fluorescent and Magnetic Activated Cell sorting (FACS and MACS)

Virtually, all stainable cells can also be isolated by FACS, including endothelial cells, immune/inflammatory cells, CAF, and tumor cells. Isolated cells can be used for functional experiments or lysed immediately to measure mRNA/DNA content by RNA/DNA sequencing or PCR or detect proteins by proteomics or antibody arrays, during natural tumor progression or in response to treatments, for example to identify pathways and their alteration by drugs [262, 264, 270]. A critical parameter for successful cell isolation is the relative density of the to-be-sorted cells in the starting population: The lower the frequency (e.g., below 1%) the longer the sorting time (which may affect viability) and the lower the purity (which can pose problems for genetic, genomic, or proteomics studies). Microvesicles can also be isolated by FACS [269]. An alternative to cell isolation by FACS is magnetic cell sorting (MACS). Cells are stained with antibodies coupled to nanosized superparamagnetic particles and isolated positively or negatively with the aid of a column and a magnet [271]. This technique allows gentle separation of a larger number of cells. While negative selection can be performed with multiple antibodies (i.e., to eliminate many different cells), positive selection can only be done with one marker at a time. MACS is often used as a first enrichment step for rare cells of interest, followed by multi-parametric FACS sorting.

### 14.2 Limitations and challenges

Despite the power of FACS to analyze multiple cellular or molecular parameters in a single experiment, there are important limitations. A main one is the absence of morphological information and position of cells. For example, the pericyte coverage of blood vessels and the organization of the vessels (size and diameter) as well as the relative positioning of immune/inflammatory cells to vessels or tumor cells cannot be obtained by FACS analysis. Having this information is important as, for example, some treatments do not decrease the number of vessels but alter their structure (“vascular normalization”) or modify the localization of immune/inflammatory cells but not their number. Thus, FACS studies should be supported by immunohistological analyses. A second limitation is that the technique is semiquantitative. Absolute cell numbers can be indirectly calculated though, by relating the relative frequency of the cell of interest obtained by flow cytometry analysis, to one main population in the test sample (e.g., CD45 ^+^cells), whose cell number was previously counted manually. Cell sorting has limitation for viability and contamination. For example, endothelial cells obtained by FACS are difficult to culture and use for ex vivo/in vitro (functional) experiments. As stated above, sorting of rare cells (< 1%) can be hampered by contaminating cells (in some cases, up to 50%). A pre-enrichment by MACS is highly recommended in such situations. As these technologies are rapidly evolving, new opportunities are emerging allowing circumventing some of these limitations or opening new opportunities. These include laser scanning cytometry for the multi-parametric study of tumor microenvironment in situ [272] and assessment of cellular/subcellular elements in individual cells [258], the use of label-free spectral measurements to analyze native individual cells [273], or the detection and imaging of circulating cells directly in blood or lymph [274], to cite only a few.

### 14.3 Concluding remarks

Flow cytometry and FACS-based experiments and analyses offer important (semi)quantitative, high content, and accurate information about tumor angiogenesis and associated cells of the TME and in the circulation. It is a remarkable powerful and robust technique, which can be used as a screening platform to test the anti-angiogenic capability of molecules and drugs for anticancer treatment or to in-depth analysis of the composition of the TME during tumor progression or in response to therapeutic interventions. FACS analysis should always be complemented with immunohistological studies to characterize the morphology of the vessels and positioning of cells of interest, particularly when investigating anti-angiogenic effects of new molecules and drugs.

## 15 Loss-of-function approaches in the developing zebrafish

In recent years, the zebrafish (*Danio rerio*) has become a highly utilized vertebrate model system for studying vascular development and angiogenesis in vivo. Vascular development in zebrafish is remarkably similar to that in mice and humans in terms of both anatomy and function. Zebrafish are genetically accessible with externally fertilized, optically clear embryos and larvae that permit high-resolution imaging of developing tissues. Fish also have large clutch sizes for experimentation, and their embryos can survive and continue to develop for several days without a functional circulation via passive diffusion of oxygen. Although the beneficial experimental attributes of zebrafish have made this an extremely appealing and approachable model system, like any model organism, there are limitations to the studies that can be performed and their interpretation. Here, we focus on loss-of-function approaches used to study vascular development in the zebrafish and address some of the pitfalls in interpreting data generated using currently available approaches.

### 15.1 Morpholinos

Prior to the advent of genome-editing technologies in the zebrafish, other than mutations fortuitously isolated in forward-genetic screens, morpholinos (MOs) were the only available reasonably well-validated loss-of-function method. MOs are specially modified stable antisense oligomers designed to block either gene translation or gene splicing when injected into embryos [275]. In the absence of reverse genetic tools, MO-based experiments rapidly gained popularity in the fish community. Although the advent of this technology was a huge step forward for the zebrafish community, over time many concerns have been raised about the specificity of phenotypes generated from MO injections, particularly the potential for off-target and/or pleiotropic effects from MO injection [275–278]. Indeed, while it is possible to show that a particular MO has the desired inhibitory effect on the target gene, it is impossible to exclude that there are (latent) unspecific effects [279]. These concerns have been greatly heightened by mounting evidence that MOs and genetic mutants for the same genes frequently do not yield comparable phenotypes [276]. In order to promote greater validity of experimental studies using MOs, attempts have been made to develop standards for their use. One recently promulgated set of “community guidelines” suggests the use of multiple MO targets, RNA/DNA rescue experiments, dose–response curves for titration of the MO, and, importantly, validation with a genetic mutant where possible [275]. It is generally accepted that proper MOs use requires rigorous confirmation of their specific blocking ability, either by assessing loss of protein production for translation-blocking MOs (if specific antibodies are available) or by assessing the generation of alternate splice variants by RT-PCR when using splice blocking MOs. MO-based experiments can also be “rescued” by co-administration of mRNA or transgenes expressing a wild-type copy of the targeted gene. However, the best and generally accepted validation for any MO phenotype is confirmation of the same phenotype in a zebrafish genetic mutant. As discussed further below, many mutants are now available from forward genetic ENU (*N*-ethyl-*N*-nitrosourea) mutagenesis screens, “TILLING” approaches [280], or genome-editing methods. CRISPR technologies have made it easy and straightforward for any laboratory to carry out reverse genetic mutation of virtually any gene of interest [278, 281–301]. In short, when possible, all MO experiments should be confirmed and validated with a genetic mutant before they are used extensively for experimental studies.

Common off-target effects noted in MOs-injected animals include p53-mediated cell death, defective circulation and “ballooning” (edema), and developmental delay/stunting of embryos and larvae. Altered vascular development and patterning should be interpreted with extreme caution in the presence of any of these phenotypes, as vessel growth and patterning in developing embryos and larvae are highly dependent on the integrity of adjacent tissues and of the animal as a whole (see below for further detailed discussion). The recently published community guidelines noted above include recommendations on how to interpret MO phenotypes, how to validate these phenotypes, and current expectations in the field for MO usage [275].

Although MOs approaches, and especially their use without proper validation, have justifiably come under a great deal of scrutiny (if not outright skepticism) in recent years due to well-documented frequent off-target effects and lack of correlation with mutant phenotypes, it is worth noting a few positive/beneficial features of MOs that make their continued use (with caution) worthwhile. First, translation-blocking MOs can block both the zygotic and maternally supplied activity of target genes, whereas maternal-zygotic mutants can be difficult or impossible to obtain. Second, MOs can be used immediately with any zebrafish line or strain unlike mutants, which need to be crossed into the appropriate genetic backgrounds (when using combinations of mutants and transgenes, this can take many generations). Third, multiple MOs can be injected simultaneously to target two, three, or more members of a gene family with potentially overlapping functions. Fourth, recent work has shown that upregulation of related compensating gene family members can sometimes occur in genetic mutants (by mechanisms that are not yet clear), while this does not appear to take place in MOs-injected animals [277], arguably making MOs a better representation of targeted loss of gene function in these cases.

### 15.2 Genetic mutants

The ability to carry out large-scale forward-genetic phenotype-based ENU mutagenesis screens for developmental mutants has been one of the major strengths of the zebrafish. Large numbers of mutants have been isolated affecting almost every conceivable developmental process, and these mutants have led to innumerable important new discoveries [302–309]. Forward genetics has been an extremely powerful approach for identifying genes necessary for developmental processes, but until recently reverse genetic approaches for targeting specific genes of interest were not available in the zebrafish. “TILLING” approaches employing high-throughput next-generation sequencing of libraries of mutagenized fish have been used to screen for ENU-induced mutations in specific genes [310] but these approaches are relatively laborious and expensive, and some genes are not as easily mutagenized. More recently, genome-editing technologies and particularly CRISPR/Cas9-based methods have made reverse genetic targeting of virtually any gene easy and cost-effective for most laboratories [281–283]. A number of resources now make identifying “ideal” CRISPR cut sites, primer selection, and mutation screening relatively simple [281, 311]. Generating specific alleles by homologous integration is still relatively challenging in the zebrafish, but the technology and resources to generate “knock-in” mutants using are also rapidly improving and are likely to soon become accessible to most zebrafish laboratories [283].

Although CRISPR mutants offer a simple and effective approach for loss-of-function analysis free of many of the caveats regarding off-target effects associated with MOs use, the resulting phenotypes must still be interpreted rigorously. Vascular phenotypes observed in animals that display significant cell death, defective circulation, edema, developmental delay/stunting of embryos and larvae—while they result from defects in the targeted gene—may represent secondary, indirect consequences of the genetic mutation and may not reflect a direct requirement for the targeted gene during angiogenesis. It is also worth noting that transient CRISPR approaches involving, for example, injection of guide RNAs into animals transgenically expressing Cas9 in specific tissues (“CRISPRi”) are subject to the same general concerns regarding off-target effects of MOs, although the spectrum of off-target effects observed with the two approaches will likely be distinct. Phenotypes observed in either MO- or CRISPR-injected animals must be interpreted with caution and ideally should be verified using a stably transmitted germline genetic mutation.

The ease and efficiency with which CRISPR mutants can be generated also makes it possible to simultaneously induce and screen for mutations in multiple genes at the same time. This is advantageous for studying the role of gene families in the zebrafish. Gene paralogues are more common in zebrafish than in mammals due to a genome duplication that occurred many millions of years ago during the evolution of teleost fish, and genetic compensation by alternate gene family members can lead to obscured phenotypes [277]. It has become more common to see reports in which double or even triple mutants have been generated in closely related gene family members [312]. As noted above, compensatory upregulation of related family members has been shown to occur in at least some genetic mutants [277], and phenotypes comparable to those noted in MO-injected animals have been unmasked by generating double or triple mutants.

### 15.3 Assessing vascular phenotypes in loss-of-function models

Whether MOs or mutants are used, assessment of vascular phenotypes involves a number of specialized considerations, since proper vessel formation can be altered or disrupted by developmental delay or by changes in circulatory flow and local or generalized defects in non-vascular tissues. Below, we discuss some general considerations in assessing vascular phenotypes and point out some potential pitfalls in utilizing and interpreting zebrafish vascular data.

### 15.4 Where is the gene expressed?

**Fig. 12:**
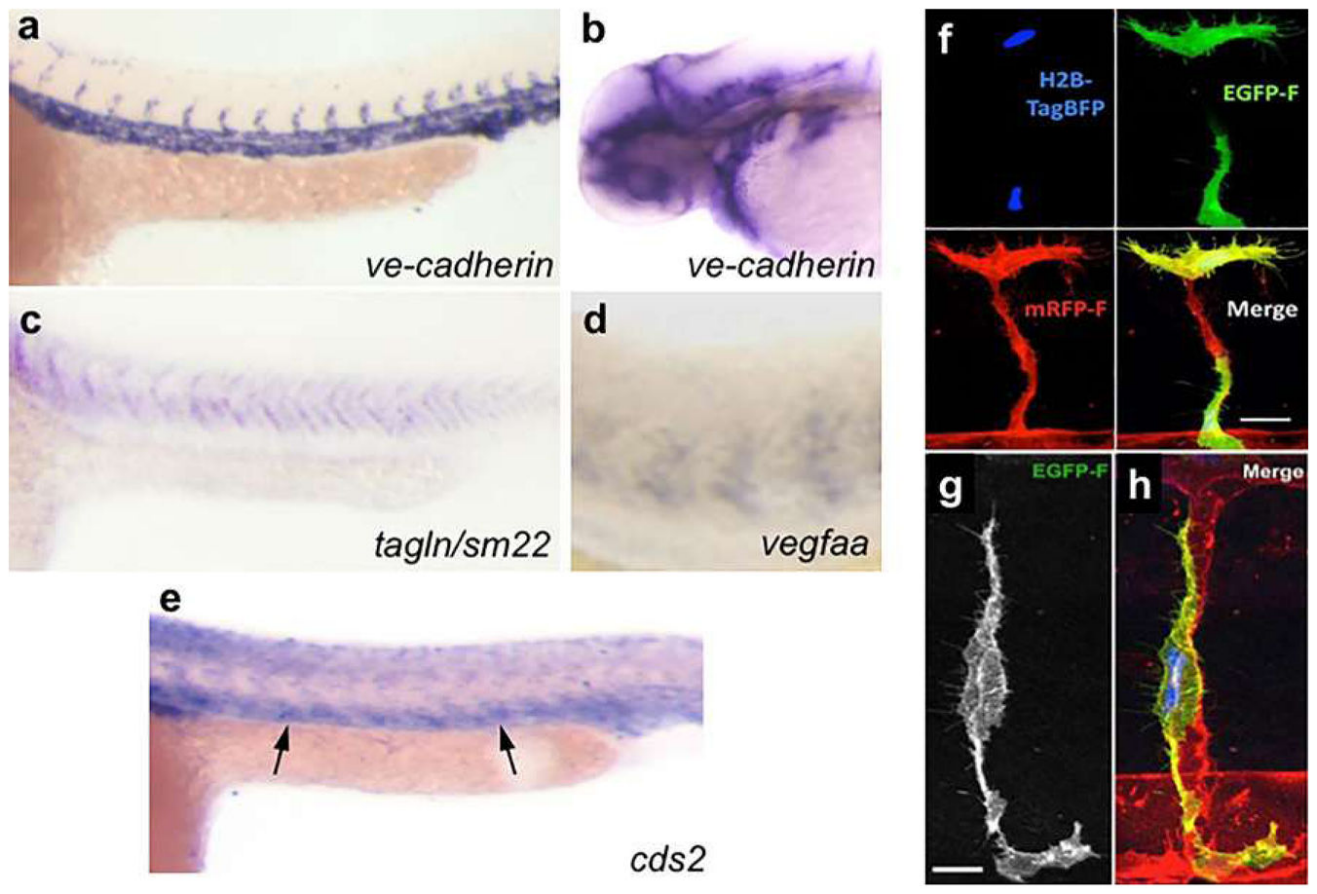
Assessing EC autonomous gene function in zebrafish. **a**–**e** Whole mount in situ hybridization of 24–48 hpf zebrafish embryos. **a**–**b** *ve*-*cadherin* labeling of the vasculature in the trunk (**a**) and head (**b**) of a zebrafish embryo showing vascular-specific labeling. **c**–**d** *tagln/sm22* (**c**) and *vegfa* [308] (**d**) showing labeling of non-vascular (somitic) tissues in the trunk. **e** cds2 [309] labeling of both vascular (arrows) and non-vascular tissues. **f** Confocal micrograph of a growing trunk intersegmental vessel in a 32 hpf *Tg*(*kdrl:mRFP*-*F*)*^y286^* embryo (red vessels), mosaically expressing *Tol2*(*fli1a:H2B*-*TagBFP*-*p2A*-*egfp*-*F*) transgene (blue EC nuclei, green EC cytoplasm), showing blue, green, and red fluorescent channels and all three merged. **g**–**h** Higher-magnification images of GFP fluorescence (**g**) and merged GFP/BFP/RFP fluorescence (**h**) in a 48 hpf *Tg*(*kdrl:mRFP*-*F*)*^y286^* transgenic animals mosaically expressing a *Tol2*(*fli1a:H2B*-*TagBFP*-*p2A*-*egfp*-*F*) transgene in a single EC in a trunk intersegmental vessel. Scale bars = 20 μm (**f**), 10 μm (**g**–**h**). Images in panels **f**–**h** are from Ref. [649]

One of the first considerations in assessing the vascular function of a particular gene, whether using MOs or mutants, is where it is experessed. This is generally assessed using whole-mount in situ hybridization (WISH) of zebrafish embryos and early larvae [313], although in older animals in situ hybridization of tissue sections can be used. If a gene shows an exclusive vascular expression pattern (Fig. 12a, b), the gene may have a vascular-specific function, and vascular phenotypes observed likely reflect a vascular-autonomous role for the gene. On the other hand, if the gene shows an exclusively non-vascular expression pattern (Fig. 12c, d), it is not reasonable to assume that the gene has a vascular-autonomous function, and any vascular phenotypes that are observed are likely indirect. In many cases, expression is observed in both vessels and in non-vascular tissues (Fig. 12e), and some commonsense judgment must be applied in assessing whether observed loss-of-function phenotypes make sense in terms of the expression pattern of the gene. If significant non-vascular expression is observed, additional experimental approaches will be needed to assess the cell autonomy of gene function, including transplantation experiments or tissue-specific transgenic expression to “rescue” the phenotype (Fig. 12f–h).

### 15.5 Assessing general morphology and development in morphants or mutants

**Fig. 13:**
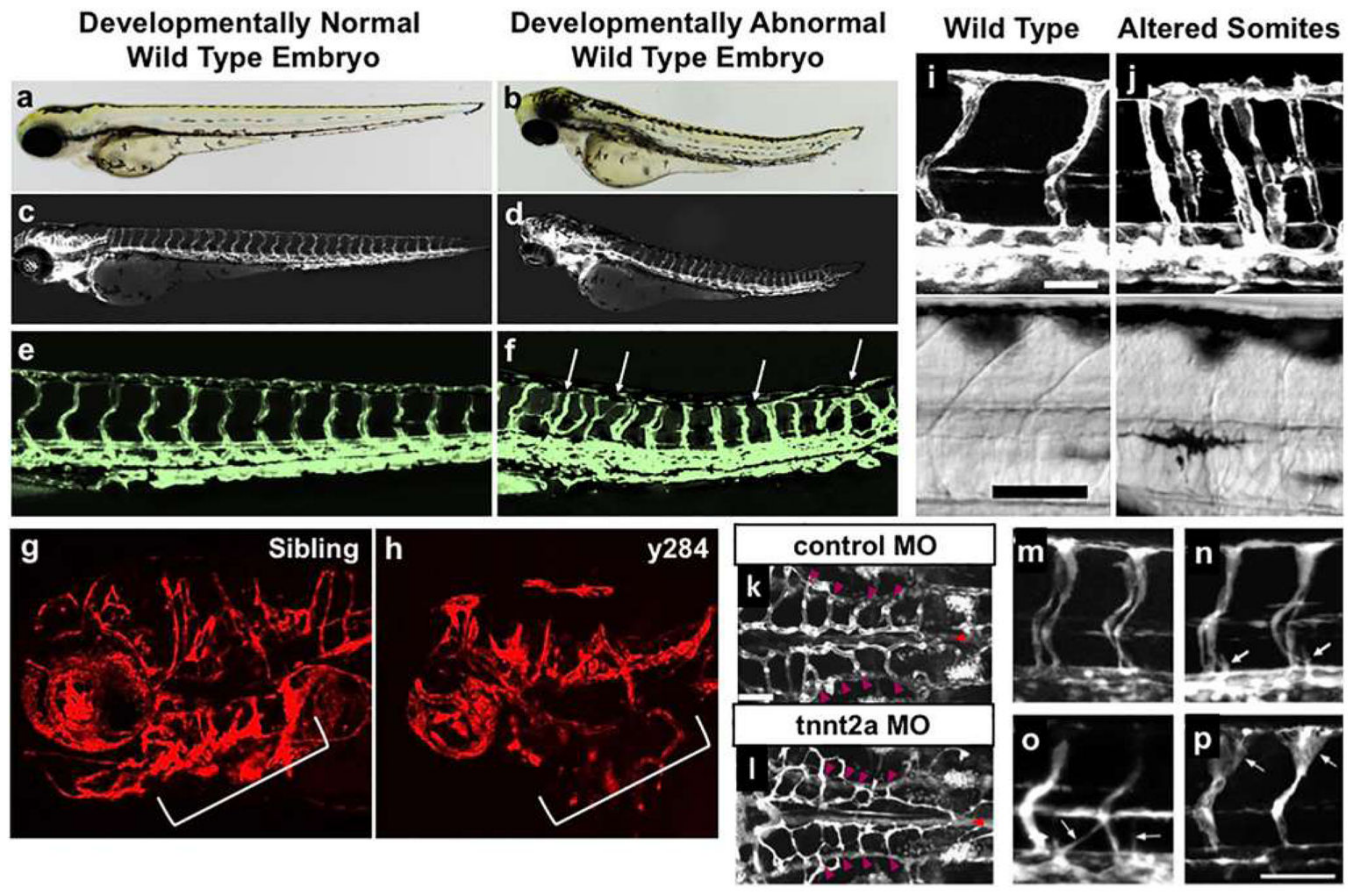
Assessing embryonic morphology and effects on vascular patterning. **a**–**f** Transmitted light (**a**, **b**), epifluorescence (**c**, **d**), and confocal (**e**, **f**) images of three dpf developmentally normal (**a**, **c**, **e**) or developmentally abnormal (**b**, **d**, **f**) embryos from the same *Tg*(*fli1:egfp*)*^y1^* transgenic “wild-type” zebrafish population. The normal animal has a normal morphology (**a**) and normal vessel patterning (**c**, **e**), while the developmentally abnormal animal has a stunted, somewhat malformed trunk (B) and also displays trunk intersegmental vessel patterning defects (**d**, **f**). **g**, **h** Confocal images of the cranial vasculature in 48 hpf wild-type sibling (**g**) and *y284* mutant (**h**) *Tg*(*kdrl:mRFP*-*F*)*^y286^* animals. The *y284* mutants have small heads heads and eyes, accompanied by reduced and abnormal formation of cranial vessels and aortic arches (brackets). The vascular defects should be interpreted with caution, as they can be primary or solely a consequence of the smaller head (or possibly both). Transplantation and/or mosaic transgenic expression can be used to assess the vascular cell autonomy of phenotypes. **i**, **j** Confocal (top) and corresponding transmitted light (bottom) images of 48 hpf *Tg*(*fli1:egfp*)*^y1^* wild-type sibling (**i**) and *fused somites (fss^y66^)* mutant (**j**) animals. Improper somite formation in *fss^y66^* mutants (lack of chevron shaped somite segments, seen in the bright-field images) indirectly results in altered intersegmental vessel patterning (**j**, top). **k**–**p** Confocal images of the hindbrain vasculature (**k**, **l**) or trunk intersegmental vessels (**m**–**p**) in *Tg*(*fli1:egfp*)*^y1^* control (**k**) or cardiac troponin T type 2a (tnnt2a) (**l**–**p**) deficient animals at 1.5 (**m**), 2 (**k**, **l**, **n**), 2.5 (**o**), or 3.5 (**p**) days post-fertilization (dpf). Control animals have normal blood flow, but tnnt2a-deficient animals have no heart beat and lack all blood flow. Although formation of the vasculature is largely normal for several days in the absence of blood flow (**l**, **n**, **o**), abnormalities in vessel growth and patterning begin to appear at later stages, such as enlargement of the dorsal intersegmental vessels at 3.5 dpf (**p**). This illustrates the need to analyze vascular phenotypes as early in development as possible in zebrafish with absent or defective blood flow. All images are lateral views, rostral to the left, except panels **k** and **l**, which show dorsal views, rostral to the left. Images in panels **i** and **j** are from Ref. [306], images in panels **k** and **l** are from Ref. [650], and images in panels **m**–**p** are from Ref. [651]. Scale bars = 50 μm

Apparent vascular-specific phenotypes often occur as secondary, non-specific consequences of general developmental delay and other localized or general changes in non-vascular tissues or organs. Staging tables are available for the zebrafish [294], and it is important to determine whether MO injections or genetic mutants result in either overall delays to development or gross embryonic/larval patterning, or localized defects in the development and morphology of specific tissues or organs (Fig. 13a, b). For morphants, this should be done by comparing animals injected with specific MOs to siblings injected with a control MOs and uninjected siblings. For mutants, they should be compared to phenotypically wild-type siblings from the same clutch of eggs. In either case, if the overall development of the animal is significantly delayed or readily apparent, gross morphological changes are noted compared to the control sibling animals and vascular phenotypes noted in these animals should be interpreted with caution. For example, reduced head size and/or extensive cell death in the central nervous system are common effects noted in both mutant and morphant models, and this secondarily results in vessel defects in the heads of affected animals (Fig. 13g, h). The timing and extent of trunk intersegmental vessel growth is frequently used as a convenient and quantitatively robust assay for assessing angiogenesis defects in zebrafish embryos, but delayed development or abnormal formation of the adjacent somites results in defects in vessel sprouting and growth that, again, are not directly linked to vascular-specific functions (Fig. 13c–f, i–j). Similarly, the formation of the thoracic duct as a readout for lymphatic development is sensitive to developmental delay and general effects on morphology; hence, the absence of the thoracic duct in itself is insufficient to demonstrate a defect in lymphangiogenesis. Staging of embryos and excluding developmental delay are critical, as is monitoring the distance between dorsal aorta and PCV in phenotypic embryos. Importantly, lack of a lymphatic system as found in *ccbe1* or *vegfc* mutant embryos [314–316] only leads to formation of edema after 120 dpf—any edema formation prior to 120dpf in experimental embryos is therefore very unlikely to result from a lymphatic defect.

### 15.6 Assessing circulatory flow and cardiac function

Even in animals with apparently normal overall development, defective blood flow (due, for example, to heart-specific defects) can affect the timing and extent of vessel growth. Assessing blood flow and cardiac function in the zebrafish is easily accomplished by direct imaging using a dissecting light microscope. For higher resolution, tracers injected into the blood stream such as quantum dots, fluorescent microspheres, lectins, and varying molecular weight fluorescent dextrans can be used to assess blood flow rates, blood flow directionality, vascular leak, vessel drainage sites, and solute uptake [317–320]. Although zebrafish can survive a number of days in the absence of blood flow, animals lacking circulation do eventually become sick, edemic, stunted in growth, and die. If a mutant or MO experiment generates zebrafish with no blood flow or decreased cardiac output, assessment of vascular phenotypes should take place as early as possible during development, during a time frame in which the animal is as healthy as possible. Although embryos and early larvae can develop reasonably normal for one or 2 days in the absence of circulation (Fig. 13k, l), overall vascular development becomes increasingly affected as time goes by (Fig. 13k, p). As a general rule, phenotypes in animals deficient in flow should be assessed prior to 6 days post-fertilization (dpf), and ideally before 3 dpf, particularly when flow is entirely absent. As noted for animals with general developmental delay or morphological defects, caution should also be taken in interpreting vascular phenotypes observed in animals that lack circulatory flow.

Quantitative assessment of blood flow and hemodynamics can be accomplished using Particle Velocimetry (PV), in which tracer particles injected into the blood stream are tracked through different vessels [321] and flow speed is determined by calculating the displacement of the particles over time [322]. Due to depth and/or the decreased transparency at later stages, PV has mainly been used in embryos or early larvae, although recent advances in confocal microscopy, ultrasound, and tomography tools [323] and methods have facilitated deeper imaging in more opaque tissues.

### 15.7 Assessing vascular patterning

Visualization and assessment of vascular patterning in the zebrafish is easily accomplished using widely available transgenic fluorescent reporter lines labeling vessels. Numerous transgenic zebrafish lines are available with cytoplasmic, nuclear, or membrane-localized fluorescent proteins expressed specifically in cardiovascular cells and tissues [324–326]. These include lines marking the heart endocardium and myocardium, blood and lymphatic vessel endothelium, hematopoietic derived cells, and perivascular cells like pericytes and fluorescent granular perithelial cells. Long-term time-lapse imaging methods have also been developed that permit continuous, real-time imaging of heart and vascular development in these transgenic lines [327]. Together, the availability of fluorescent transgenic reporter lines and methods for high-resolution, dynamic imaging of these lines has revolutionized the study of cardiovascular development.

Alternatively, or in addition, intravascular injection of fluorescent tracers such as quantum dots, fluorescent microspheres, or fluorescent dextrans, or non-fluorescent tracers such as India ink, Berlin blue, or Evans blue dyes can be used to visualize zebrafish blood vessels and their patterning [317, 319, 327–332]. Although the wide availability of vascular-specific transgenic lines has reduced the need for these traditional methods for examining the patterning of developing vessels, microangiography is still the method of choice for assessing vascular integrity [333], vessel lumenization, and flow (as noted above) in blood vessels, as well as permeability, drainage, and solute/fluid uptake in lymphatic vessels. The microangiographic techniques are fast, robust, and cost-effective methods for visualizing the vasculature and can be used to visualize vessels in non-transgenic animals or at more mature developmental stages.

The patterning of vessels in genetically or experimentally manipulated animals can be examined for alterations including reduced/absent vessel growth, excessive vessel growth or branching, altered patterning of vessels, changes in flow patterns/vascular connections, etc. Changes can occur either broadly throughout the animal or may be localized to particular vascular beds. Up to approximately 7 dpf, the pattern of blood vessels observed should be compared to the published staged atlas of vascular anatomy [328]. Although the positioning of major vessels (e.g., dorsal aorta, cardinal vein, and intersegmental vessels in the trunk, or basilar artery in the head) is relatively invariant in normal animals at given stages, the precise location and paths taken by many smaller and/or later forming vessels (e.g., the central arteries of the hindbrain) are less stereotypic, and minor changes in the patterning of these vessels may not be significant. As noted above, it is important to assess vessel defects in genetically or experimentally manipulated animals in the context of the animal as a whole, including whether any developmental delay or gross morphological defects are present. The timing and extent of intersegmental vessel sprouting is frequently used to assess angiogenic phenotypes in developing zebrafish, but their patterning can be strongly affected by developmental delay, non-vascular morphological defects, and reduced or absent circulatory flow (Fig. 13).

### 15.8 Concluding remarks

A variety of approaches for reverse genetic targeting of specific genes for loss-of-function analysis are now available in the zebrafish, most notably MOs and CRISPR-generated mutants. Although MOs have been somewhat discredited in recent years as a “first line” tool for assessment of loss-of-function phenotypes, they remain valuable when rigorously validated by a corresponding mutant with the same phenotype. Furthermore, in some cases, mutants are susceptible to genetic compensation by upregulation of related genes, while morphants are not. Detailed “community guidelines” for the use of MOs were recently published and should be referred to [275].

Any researcher generating loss-of-function vascular phenotypes in the zebrafish (or in any other model organism, for that matter) needs to critically assess their data in light of some key questions. Where is the gene expressed? Do mutant or morphant phenotypes make sense when compared to the expression pattern of the gene? The more “vascular-specific” expression is, the more likely the associated vascular phenotypes are specific. Conversely, if a gene is ubiquitously expressed throughout the animal, the burden of proof is on the researcher to determine the vascular cell-autonomous function of the gene, using transplantation, tissue-specific transgene “rescue” experiments, or other methods. Do mutants or morphants show significant developmental delay or gross morphological phenotypes, and do the vascular phenotypes most likely reflect developmentally “younger” animals or problems in non-vascular tissues? Do the mutants or morphants have absent or strongly reduced circulatory flow? Are vessel phenotypes being assessed in these animals at a time point prior to lack of flow causing significant general defects in development? Taking all of these criteria into consideration when assessing vascular phenotypes resulting from gene knockdown or genetic mutants is important to ensure that reliable, valid conclusions are made when studying regulators of angiogenic development utilizing the zebrafish.

## 16 Chorioallantoic membrane assays

**Fig. 14:**
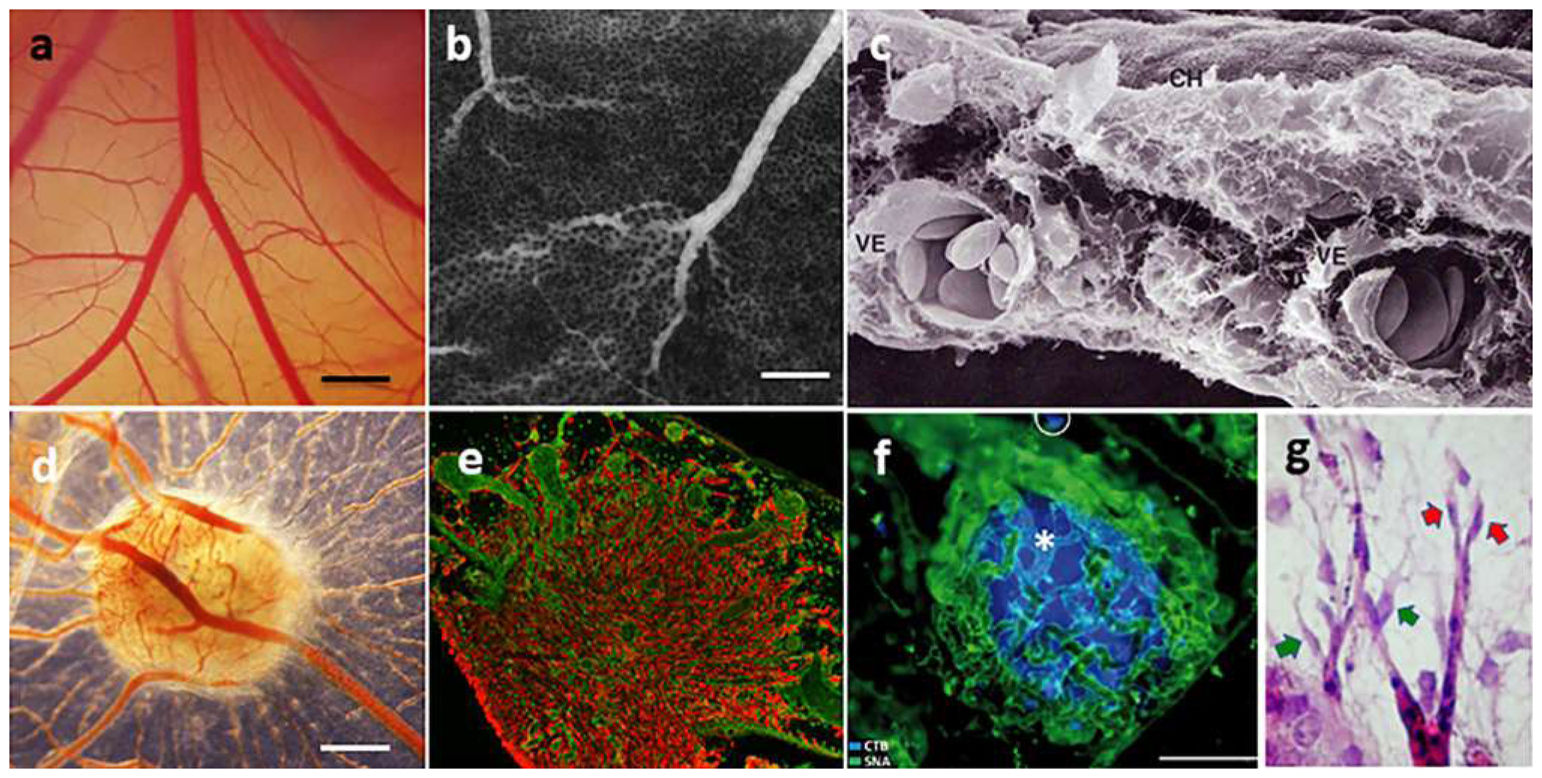
Chorioallantoic membrane of the chicken embryo (CAM). **a** Bright-field image of the CAM vasculature presenting the functional vascular network from capillaries to big vessels imaged at embryo development day 10 (E10). Bar corresponds to 500 μm; **b** Vascular network of the CAM presented on the fluorescent angiography with FITC-dextran and an intravenous contrast agent. Bar corresponds to 500 μm; **c** Scanning electron microscopic picture showing the chorionic epithelium (CH) and two large vessels (VE) in the intermediate mesenchyme of a CAM at day 5 of incubation (reproduced from [652]); **d** A 12-day CAM incubated on day 8 for 4 days with bioptic specimen of ACN/neuroblastoma cell line tumor xenograft, showing numerous blood vessels around the graft (reproduced from [653]). Bar corresponds to 500 μm; **e** Immuno-histology of a glioma (U87 tumor) implanted on the CAM with tumor cells in red (vimentin staining and vessels in green(SNA isolectin staining), magnification ×4; with permission from [362], copyright (2005) National Academy of Sciences, USA; (**f**) a pancreatic adenocarcinoma (BxPC3) nodule (blue, Hoechst) inside of the CAM surrounded by blood vessels (green, SNA isolectin staining). bar corresponds to 100 μm; with permission from [363], copyright, Elsevier (License number 4307171269037); **g** MCF7-derived tumor implanted on CAM induces angiogenic response. Tip cell (red arrow) and incompletely attached pericytes (yellow arrow)

Although the chicken chorioallantoic membrane (CAM) assay (Fig. 14a) appears to have been first reported by Rous and Murphy in 1911 [334], more than a century ago, to study xenoplastic growth of mammalian tumors, the chick embryo itself has been a target of scientific study beginning with Aristotle. Due to the fact that fertilized chicken eggs are readily available and the embryos are easily visualized, chicken embryos became a favorite target for experimental biologists and, not surprisingly, the CAM assay became one of the classic procedures for students, being included in one form or another in virtually every college laboratory manual. Due to these advantageous properties, the CAM has been widely used not only for direct vascular biology studies, but also in bioengineering, cosmetics testing, transplant biology, drug and vaccines development, vaso-occlusive therapies, or cancer research [335–343]. There are certainly several hundreds of variations in the experimental protocol, all of them involving placing a test material on the CAM or an intraveneous injection. The variables, however, often largely unbeknownst to the user, lead to highly significant differences in results and a striking diversity in observations and conclusions. Several aspects of the diversity are described below.

### 16.1 In ovo CAM assays

The “standard” (i.e., “classic”) assay consists of exposing the CAM in the incubated egg by making a window through which the CAM can be accessed and placing a test graft, implant, or substance on, in, or through it. After resealing the window, periodical examination of the test area can be performed, ultimately measuring the result, frequently by harvesting the area for chemical or histological analysis.

#### Incubation temperature

The standard Hamburger and Hamilton stages of chick embryo development for stages 14–35 were based on incubation at 39.4 °C using White Leghorn chickens [344]. The other stages were based on incubation temperature of 37.5 °C. Stage 33 (7 days) is when many investigators place a graft or sample on the CAM. This may sound trivial, but at stage 33 the CAM is slowing its growth. If the CAM is accessed on day 7 of an embryo incubated at 37 °C (common in most laboratories), CAM cells are still rapidly proliferating [56]. Moreover, depending on the source and strain of eggs used, there may be a 12–18 h variation in developmental age.

#### Making a window

The original method used a handheld saw blade, which worked well but frequently led to debris falling on the developing CAM. Such debris can provide artifacts difficult to distinguish from results due to planned experimental protocols. Ed Zwilling (in 1952) had a Dremel drill sanding disk in his garage which worked well and is now used nearly universally, but even so, debris, often too fine to see, can materially influence the results [56].

#### Placement of test material/grafts

The CAM itself is, as indicated by its name, multilayered. Placing a filter or graft on the membrane is quite different from inserting it part way (preferred by many) or penetrating the CAM completely so that it projects through the entire membrane. Results may be completely different depending on the mode of insertion as well as on the physical forces used [345]. A second variable is the choice of location on the membrane. The CAM at stage 30–35 is highly vascularized (hence, a good choice for transplants), but there are vessels of all sizes. Choosing a site near a large vessel sets up conditions quite different from choosing a less vascular site. Sprouting will depend on placement as well rate of dispersion of test substances and the elution from disks and filter membranes. The reader is referred to several excellent comprehensive reviews that describe a wide range of modifications and adaptations of the basic CAM assay [335, 346–351].

Day 7 of the CAM incubation represents the proper time to place grafts on the CAM surface for investigation of angiogenic responses. There are several reasons why this time for grafting is preferential: (1) Areas between big vessels are less vascularized and the assessment of angiogenic events is more objective, (2) the immune system is not yet developed, and (3) interaction between immature developing mesoderm of the CAM and a graft gives a proper microenvironment for the survival, development, and vessels acquisition by angiogenic grafts.

### 16.2 Ex ovo CAM assays

Once it was demonstrated that one can carry out CAM assays in shell-less egg cultures [352, 353], this modification became a staple variant of the assay. Again, there have been numerous improvements and variants, but all involve transferring the egg content at an early stage (day 2–3) into a container, allowing the CAM to develop and then carry out procedures as in the standard in ovo protocol. The major modification from the original petri dish method is the use of a curved rather than flat-bottomed dish, and modifications have ranged from specially designed watch glass-type vessels to plastic wrap loosely fastened over emptied food cans. Major advantages over in ovo methods are better visibility, potential to use transmitted light, ready application of several test sites, time-lapse monitoring, and multiple manipulations. Disadvantages include significant mortality, greater risk of infection, the need for more rigorous control of the incubation environment, and materially greater expense.

### 16.3 Angiogenesis platform using a cubic artificial eggshell with patterned blood vessels

One of the newest models using the chick embryo chorioallantoic membrane as a tool for blood vessels assessment and dynamics was recently described by Huang et al. [354]. The authors provided a methodology to direct blood vessel formation on the surface of a three-dimensional egg yolk using a cubic artificial eggshell with six functionalized membranes. By using this method, blood vessel assessment of the CAM is improved by controlling their growth directions in special microfluidic chambers with a width of 70, 250, and 500 μm. Since the blood vessels with blood flow in microchannels are still alive, several culture chambers for implanted cells/tissues can be fabricated in the patterned surface during one experiment. Because the cubic eggshell with patterned surfaces is transparent, reagent injection and screening is possible in each of the chambers separately, and the development and spreading of blood vessels into the cultured tissues in the chamber can be observed using a microscope [354].

### 16.4 Assessing vascular patterning

Visualization and assessment of vascular patterning in the CAM model is one of the most challenging aspects of the assay. Quantification of angiogenesis by appearance (number of vessels and their hierarchy, degree of branching, etc.), whether by simple inspection or quantitative image analysis, is highly variable. To obtain statistically significant data requires multiple samples. Monitoring during the course of the experiment is often foiled by the spreading and hence moving of the test site from the observation window. Most importantly, many of the visible measurements cannot distinguish well between neovascularization and vasodilation or between inhibition and vasoconstriction. Several semiquantitative or quantitative imaging [355, 356] or immunohistochemistry-based [357] methods for vascular and capillary pattering assessment have been developed. The descriptors include, for example, branching points/area, vessel or capillary density, or mean mesh size. Apart from intravenous injection of fluorescent dextrans (Fig. 14b) [358, 359] or non-fluorescent tracers such as ink [360, 361], MRI and PET imaging, scanning electron microscopy (Fig. 14c), or optical Doppler tomography are now available to facilitate the visualization of the CAM vasculature (see a comprehensive review [335]). For some of these techniques, however, the resolution might be an important limitation and should be considered prior to imaging technique selection. Another point to take into account in imaging technique selection is the vascular development stage, vascular permeability, and lymphatic uptake. Recently, imaging was facilitated by the replacement of the original eggshell by an artificial one [354].

### 16.5 The experimental chicken embryo tumor model

Tumor cells can easily be grafted on the chicken CAM and develop vascularized tumor (Fig. 14d). The way tumor implantation is done is dependent on the tumor type that is studied, see Table 2. In the case of glioblastomas, when implanted onto the CAM [362] (Fig. 14e), a plastic ring is placed on the surface of the chorioallantoic membrane at embryo development day 10 (E10). Gentle lacerations of the CAM surface are carried out with scalpel blades in order to facilitate and promote tumor engraftment. Implantation of four million tumor cells inside of the plastic ring in serum-free culture medium is usually used with daily monitoring over seven to 8 days. At E17 or E18, remove the plastic ring by cutting out the CAM area and proceed for analysis (OCT inclusion, snap-frozen, etc.). Tumors such as the highly angiogenic glioblastomas (U87 etc.) are growing very well with an angiogenic response that starts at day 3–4 post-implantation.

In case of highly metastatic tumors such as pancreatic ductal adenocarcinomas (PDAC), the procedure is a little different [363]. In this case, tumor cells are deposited directly inside the plastic ring without previous laceration of the CAM. Tumor nodules are invading deeply the CAM tissue and are surrounded by blood vessels [363] (Fig. 14f). CAM tumor samples can then be analyzed using various methods (qPCR, Western blot, immunolabeling, etc.).

Another variant is the CAM metastasis assay [364]. This assay can be used either to determine spontaneous metastasis of cells deposited on the CAM or to determine experimental metastasis after intravenous injection of tumor cells in the chorioallantoic vein. If the metastasis assay is not strictly speaking an angiogenesis assay, it allows, however, the study of tumor cell dissemination through the vasculature.

### 16.6 Functional genomics analysis using the chicken CAM model

#### Transcriptomic analysis of the CAM

Transcriptomic analysis can be performed on the CAM tissue during development, after stimulation by exogenous factor or after tumor implantation [362, 363]. CAM tissue can either be processed as a whole, or vessels can be micro-dissected and analyzed by qPCR, micro-array, or RNAseq. In case of tumor implantation, the signals coming from the tumor and the stroma can de differentiated because there is only 5% overlap. Bioinformatics analysis may help to identify the vascular cell signature of genes identified by determining vascular-specific ESTs and an angioscore [365].

#### Proteomic analysis of the CAM

Proteomic studies can be done on the whole CAM tissue (snap-frozen after dissection) or can be carried out by in vivo biotinylation of the chick embryo using shell-less embryo cultures [366]. The experimental procedures can be divided into a number of independent steps: (1) embryo cultivation; (2) perfusion and biotinylation of chicken embryo and its extra-embryonic vasculature; (3) lysis of selected tissue or organs and purification of proteins; (4) deglycosylation of proteins; (5) proteolytic (trypsinization) digestion of proteins; (5) mass spectroscopy of peptides obtained from proteolytic digestion of proteins and measurement of peptide masses and their relative representation within the sample; and (6) statistical identification of original proteins from known genetic database after calculation of nucleotide sequence from acquired peptide masses. Also, in this case, bioinformatics analysis may help to identify the vascular cell signature of genes identified by determining vascular-specific ESTs and an angioscore. A detailed protocol can be found in [367].

### 16.7 Limitations and challenges

The CAM assay is a model of developmental angiogenesis. Exponential growth of new blood vessels and capillaries occurs between embryo development days 5 and 9. For testing drugs or transplants, it should always be realized that effects are observed in the context of this developmental background. After Day 9, the CAM vasculature is fully established and allows investigation under conditions without this background angiogenesis. By evolutionary point of view, chickens and humans are far from each other, but a paper published by the International Chicken Genome Sequencing Consortium [368] highlighted a high similarity between human and chicken genome, proving that 60% of chicken genes have counterparts in humans. Although this might be a an argument in favor of the use of the chicken CAM, we need to be conscious for species differences and consider other models to validate experimental results. It should also be realized that the chicken model is genetically further remote from the human system, which in some cases induces difficulties using the CAM. Although the model is very attractive for many researchers, the chicken embryos hatch around development Day 21. It is therefore recommended not to exceed day 18 for experimental testing. In some countries, regulatory rules apply after development day 14–15, resulting in limitations of experimental testing for only 10–11 days. Nonetheless, there is little doubt that the classic CAM assay has produced a remarkable array of highly valuable information about neovascularization, inhibition of angiogenesis, normal xenograft behavior, and tumor growth and development. Its relative simplicity, its avoidance of many of the regulatory rules placed on live animal research, as well as its low cost have made it a reasonable assay for preliminary screening. In the case of proteomic analysis when using the biotinylation technique, there may be problems with biotin penetration since biotin may diffuse in the surrounding tissue in excess. This may lead in picking-up proteins not strictly linked to the vasculature. Thus, the duration of the biotinylation procedure is critical. Harsh conditions of protein elution, which can be a problem, which may lead to contamination of the protein pool by non-specific proteins. Despite the lack of its immunity, chick embryo CAM does not support implants from tissues rich in lymphocytes as colon and lymph nodes, nor implants derived from bone marrow. This is considered a limitation of the model, but also a challenge to find alternative ways to use such tissues in the CAM model.

### 16.8 The CAM as a screening platform

The CAM model has been used for various applications in various fields of research. Many excellent papers and reviews summarize these applications, showing that each application demands the use of specific protocols (Table 2).

### 16.9 Concluding remarks

Even in the last 3 years, close to a thousand publications have employed CAM assays. Whereas classically trained embryologists are well aware of the complexity of embryogenesis and are almost overly cognizant of the problems encountered when working with a rapidly changing target, researchers less acquainted with the vagaries associated with developing embryos may need to develop a grudging respect for the intricacies underlying the CAM assay. This being said, it is deservedly a most important technique, major contributions have resulted from its use, and no doubt it will continue to be an important tool in angiogenesis research.

## 17 Murine allantois assay

The allantois is an extra-embryonic structure, which undergoes vasculogenesis, vascular remodeling, and angiogenesis and is pivotal in establishing the chorioallantoic placenta and umbilical circulation. The allantois is characterized by a mesenchymal core and by an enveloping mesothelium and appears at E8.0. Vasculogenesis starts in the distal allantois and forms a plexus that connects with the dorsal aorta of the embryo (E8.25) before the chorioallantoic fusion (E8.5), which is instrumental in placenta formation (E9.5). This plexus undergoes a deep remodeling resulting in the formation of umbilical artery and vein, which invade the chorion by sprouting angiogenesis. These processes recapitulate the molecular and genetic distinct features of developing vasculature in embryo and in adult life, including the role exerted by signaling and transcriptional pathways triggered by established angiogenic inducers and modulators [369, 370], such as VEGFs, PDGFs, ANGPTs, ephrins, NOTCH ligands, and WNTs.

Even if less known than other angiogenesis assays, the murine allantois explant assay represents a powerful tool to investigate general mechanisms of blood vessel formation, including the remodeling of a primitive vascular plexus, the angioblast differentiation, the arterial and venous fate, the sprouting angiogenesis, and the maturation of the capillary network by mural cell recruitment. In particular, this assay allowed reaching seminal contributions to vascular biology. For example, it was exploited to investigate the role of VE-cadherin, vascular endothelial protein tyrosine phosphatase, and sphingosine-1-phosphate in angiogenesis [371–374].

### 17.1 Overview assay

The allantois is dissected from E8.5 mouse embryos using tungsten needles and is placed individually on collagen- or fibronectin-coated coverslips in eight-well culture dishes (BD Biocoat). Explants are cultured in 0.5 ml of culture medium (DMEM 4.5 g/l glucose, 10 mM L-glutamine, Pen-Strep), containing 15% fetal calf serum for 18 h. Explants are then washed and fixed in 4% paraformaldehyde or methanol:DMSO (4:1) for 20 min at room temperature and processed for immunohistochemistry or TUNEL assay.

Pregnant female mice are killed on day 8 of gestation (approximately E7.75) or the following day (approximately E8.75). It is important to synchronize mice mating to dissect the allantois at the proper stage [375, 376]. Uteri are placed in Dulbecco’s A phosphate-buffered saline for isolation of decidua that is placed in Hepes-buffered DME medium with 7.5% fetal calf serum. With the aid of fine forceps and a scalpel, the embryo is isolated from the uterine tissues under a stereomicroscope. In the embryo, two structures can be recognized: embryonic tissues (clear and cylindrical shaped,) and the ectoplacental cone (reddish, cone shaped, and located at the end of embryonic tissue in the uterine mesometrial region). Allantoises are mouth-aspirated into a hand-pulled glass microcapillary (60–120 μm diameter). The ectoplacental cone/chorion should cleanly break off, leaving an opening to the exocoelomic cavity for the easy aspiration of the allantois. Immediately after inflating the exocoelom, which allows better visualization of the allantois through the yolk sac, the tip of the microcapillary is aimed toward the distal tip of the allantois and, while gently aspirating, “sheathe” the entire length of the allantois with the tip of the microcapillary. While maintaining minimal aspiration, lift the allantois, still in the exocoelom, toward the meniscus. At the meniscus, gently suction the allantois into the microcapillary, thus leaving behind the embryo, which will drop to the bottom of the dish. The allantois can now be manipulated [375].

Three different methods can be used to maintain the allantois in culture. It can be kept in suspension in rolling cultures allowing the development of a 3D spheroid characterized by a vascularize core enveloped by a mesothelial layer. Explants are cultured in 0.5 ml of DMEM medium containing 50% rat serum or 5% (“low serum”) or 50% fetal calf serum. To culture longer than 1 day, explants are given completely fresh gas-equilibrated medium at 24 h intervals [375, 376].

A second system is based on the hanging drop system. The allantois is suspended in 0.03 ml of DMEM supplemented with 10% FCS and located onto the underside of a lid of a plastic bacterial dish. The hanging drop cultures are placed into a 5% CO_2_ incubator, and after 18 h, the originated 3D spheroids can be resuspended in the above medium containing angiogenic inducers (e.g., VEGF) and the hanging drop procedure is repeated [377]. Finally, allantois can be easily maintained in adherent conditions (50% rat serum in DMEM medium) up to 72 h on glass surface or on plastic surface coated with fibronectin or poly-lysine and differently stimulated [370, 374, 378]. Allantois isolated at E8.0 adheres within 12 h, and after 18–20 h of culture, the explant adopts a circular shape with a vascular plexus, which covers the central area.

### 17.2 Limitations and challenges

The main positive feature of this assay is that it can analyze vasculogenesis or angiogenesis dependent on the explant isolation. Actually, when the allantois is isolated early (E8.0, headfold stage), explants undergo vasculogenesis, and when isolated later (E9.5, 22–26 somites), undergo angiogenesis [370, 371]. Second, allantois explants can be imaged with the use of time-lapse microscopy to follow the sequence of events that occur during vessel formation in vitro [372]. Finally, cultured allantois explants can be immunostained for markers of vessel formation and can be sectioned for histological analysis. It is further evident that allantois isolated from genetically manipulated mouse models can be useful to understand the role of specific genes in vascular development. Finally, because the allantois is implicated in placenta vascularization, this model can be specifically exploited to investigate pathogenetic mechanisms of placental diseases. Negative aspects of the murine allantois assay are the short time of culture viability (72 h) and a solid experience in manipulating embryo mice.

### 17.3 Concluding remarks

The allantois angiogenesis assay is not so popular and widely used in the vascular biology community but it is useful to discriminate if a vascular phenotype is primitive or secondary to other embryonic genetic defects, in particular when the genetic manipulation is early lethal. The robustness of the assay also validated by computer-assisted analysis [379] can be exploited in drug evaluation as recently reported [380]. Thus, the allantois is an important tool at infancy to address relevant issues of vasculogenesis and angiogenesis in development and disease.

## 18 In vivo angiogenesis plug assay

A widely used in vivo assay for the evaluation of pro- or anti-angiogenic factors is the in vivo angiogenesis plug assay, which makes use of basement membrane extracts (BME) or Matrigel. This assay is reliable, easy to perform without the need for special equipment, reproducible, quantitative, and quick [381–383]. The assay is performed by injecting, for example, 0.1 ml BME/Matrigel in the subcutaneous space of an animal. The liquid solidifies to form a plug at body temperature. Over time, blood vessels will sprout into the plug. With two plug sites per animal, the setup is quick and allows for multiple test compounds or concentrations to be tested. Thus, drug screening can also be evaluated for effects on the activity of angiogenic or anti-angiogenic factors [384–387]. The drug can either be placed in the plug together with the test factor by mixing with the Matrigel matrix or be given to the host animal. Cells or even exosomes can also be tested when mixed into the gel for their production of angiogenic factors. Furthermore, the assay is highly versatile. For example, the role of certain genes can be evaluated using genetically modified mice (overexpressing or ablating a protein gene), or animal models of diseases, such as aged or diabetic animals. Also, the effect of certain drug treatments can be evaluated. Matrigel is a mixture of basement membrane components derived from an animal tumor [388, 389]. It contains predominantly laminin-111, collagen IV, heparan sulfate proteoglycans, and various growth factors. Matrigel is a liquid at 4 °C and gels at higher temperatures. It is used in various angiogenesis assays, including the tube formation, aortic ring, and plug assays.

### 18.1 The in vivo plug assay method

In this assay, the test compound and/or growth factor is mixed with a basement membrane extract at 4 °C, known as Matrigel, and then injected, while still cold, subcutaneously into mice where it will gel. After approximately 1 week, the plug is excised and assayed for angiogenesis by various methods, including histology, the Drabkin assay (hemoglobin content), immunofluorescence, quantification of fluorescent Dextran injected intravenously just before plug harvest, etc. [381, 382, 384–387]. Complete protocols have been published [382, 387].

**Fig. 15:**
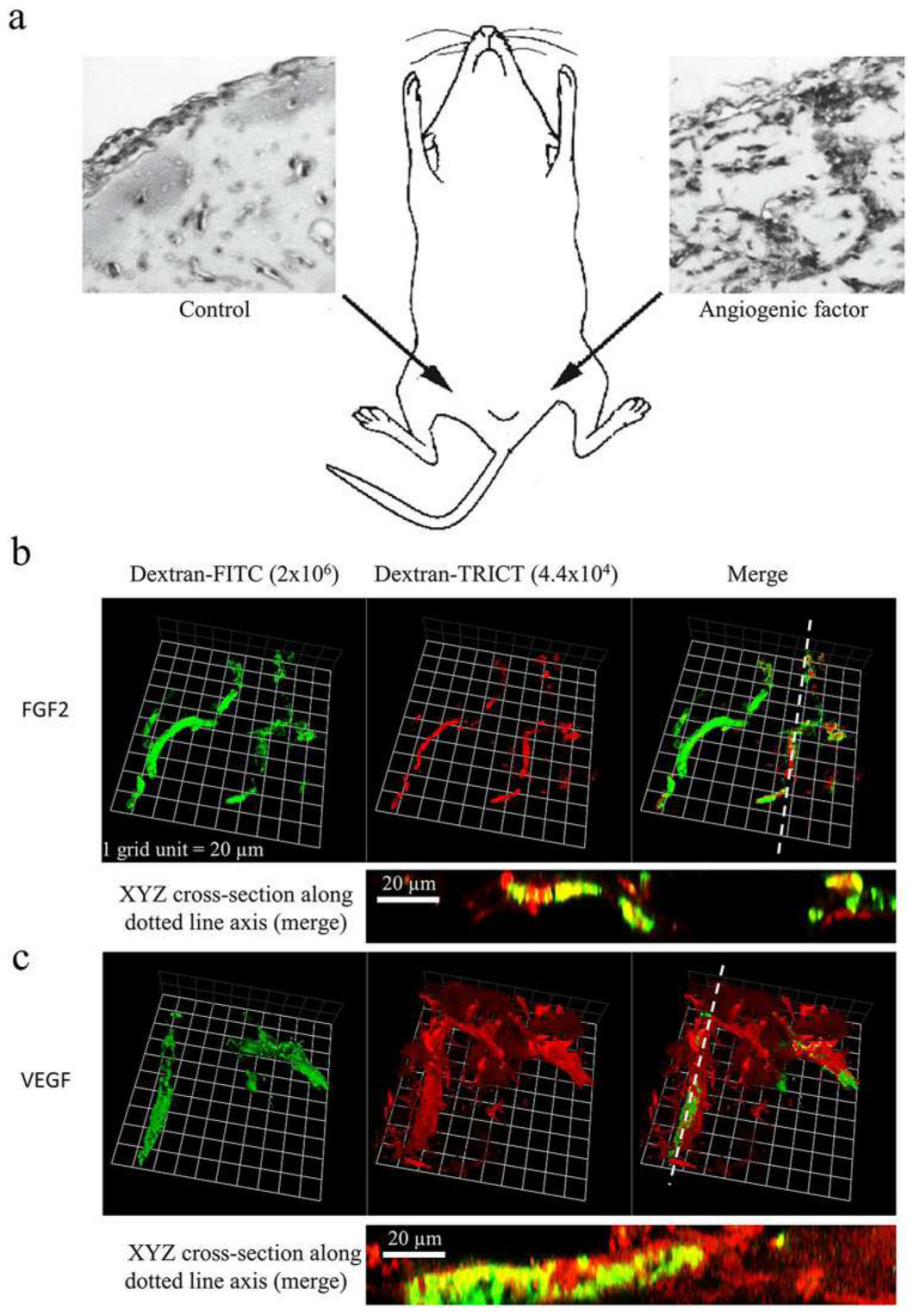
In vivo BME/Matrigel plug assay in mice. Injection of BME/Matrigel in the groin/abdomen areas of a mouse. The left image is a plug without growth factors; the right image represents a plug with an angiogenic growth factor. **b**, **c** Confocal microscopy images of whole-mount Matrigel plugs, with labeled dextran tracers. Matrigel plug angio-genesis induced by (**b**) FGF-2 resulted in stable vessels, with co-localization of large FITC-dextran and small TRITC-dextran within the vessels, which appear as yellow (XYZ planes). Matrigel plug angio-genesis induced by (**c**) VEGF resulted in highly permeable vessels, with most TRITC-dextran extravasated outside the FITC-dextran positive vessels. Images were captured using a Carl Zeiss LSM510 META or LSM 780 confocal microscope. Volocity^®^ software (Perkin Elmer) was used to reconstruct 3D images of the vessels from serial Z-sections

The assay requires the use of BME/Matrigel without phenol and with low content of growth factors. BME/Matrigel is thawed overnight on ice in the refrigerator, because it gels at a temperature above 4 °C and is not usable. BME/Matrigel can be supplemented with an angiogenic inducer, and it is mandatory to mix it well (do not vortex as bubbles will form) with the BME/Matrigel at 4 °C without diluting the matrix as this reduces the gelling properties. The test mixture or the control without test materials should be cold when injected as it will gel quickly in the subcutaneous location due to the warmth of the animal. Injection is done with 1-inch 21–25 g needles, which are changed after 2–4 injections as they may become dull. Mice (*n*= 3) are slowly injected with 0.1 ml into both groin areas (Fig. 15) with the tip of the needle as far as possible from the injection site to prevent leakage. A bump should appear at the site where the test material is released from the tip of the needle. It is preferable to hold the syringe in place for about 30 s to allow the test material to gel. The syringe is gently rotated to remove the needle to help further seal the injection site hole.

At the end of the experiments, mice are killed. For the harvest, a square segment of skin containing the plug is excised. Alternatively, the skin can be cut around the plug leaving plenty of space so as not to damage the plug. Then, the underside is exposed. The plug is small and yellow in color but it may also be pink or red depending on the degree of angiogenesis. The control plug depending on the assay method will be colorless. The plug is safely and gently removed with scissors, embedded in HistoGel, and fixed for histology, followed by sectioning and staining with Masson’s trichrome. Most of the angiogenesis will be from the edges of the plug, and the quantitation can be based on the density of the vessels and/or extent of in growth toward the center of the plug. At least three fields per plug located at approximately the same distance from the edge of the plug are analyzed. Alternatively, Drabkin reagent can be used to assess to the amount of blood in the plug or fluorescein isothiocyanate dextran (Mw 150,000–200,000) can be injected into the tail vein of the animals and extracted from the plug for quantitation. However, these approaches can be flawed by either the presence of compressed non-perfused vessels or by the presence of leaky blood vessel or hemorrhagic areas. Some researchers isolate RNA from the plugs and use qPCR of EC genes [390] as a quantitation assay.

### 18.2 Key steps in 3D imaging and analyses

Matrigel is very amiable to a variety of fluorescence labeling techniques and generation of 3D images, due to being optically translucent and highly permeable. Intravenous injection of large (200,000 Mw) fluorescein isothiocyanate (FITC)-labeled dextran identifies functional vessels within Matrigel, while smaller (4400 Mw) tetramethylrhodamine (TRITC)-labeled dextran can be used to highlight the permeability of these vessels. Figure 14 shows an example of differences in vascular permeability detected with this method. Stable vascular networks with low permeability are induced by FGF-2 (Fig. 14b), whilst highly permeable vessels are induced by VEGF (Fig. 14c). To achieve these images, labeled dextrans at 10 mg/ml each are mixed in a 1:1 ratio in 100 μl, and injected i.v. 15 min prior to euthanasia. Excised Matrigel plugs can be fixed in 4% PFA for 2 hours and then stored in 70% ethanol. Using a scalpel, all skin or connective tissue is removed and then Matrigel plug slices of ~ 300–500μm are prepared for whole-mount imaging using confocal microscopy. Antibody labeling, such as VE-cadherin (clone BV13, eBioscience) and endomucin (clone V7C7, Santa Cruz) can be performed over-night using a permeabilization buffer containing 1 % BSA, 0.5 % Triton X-100. High resolution imaging is carried out using confocal microscopy, to acquire a series of Z stacks, which can be used to generate 3D projections using software such as Volocity. Permeability can be identified by the presence of extravasated TRITC-dextran, which is quantified and normalized against FITC-dextran [391].

### 18.3 Limitations and challenges

As the age and gender of mice can influence the results of this assay, it is important to standardize these parameters. Young C57BL/6 mice are recommended as Matrigel is derived from this mouse strain. Immunodeficient mice can also be used (when heterologous cells are mixed in the gel), but are more costly. It is important to note that there can be considerable variability in the plug responses under the same treatment; therefore, the use of 3–6 mice per data point is recommended, with two injections in each mouse. For pro- and anti-angiogenic agents that are tested as a local application in the gel, it is important to establish their residence time in the gel. Some known pro-angiogenic factors are known to leak out too fast to be effective.

The Matrigel should be handled according to the instructions. If the Matrigel has solidified or contains particulate matter after thawing, it should not be used, because this may interfere with the even distribution of the test material and can yield artefacts. When mixing the test material with the Matrigel, it is not recommended to vortex, as bubbles may be formed that will distort the gel in vivo. Letting the mixture stand on ice for 10 min will reduce bubbles. Diluting the Matrigel matrix with too much test substance volume will reduce the gelling capacity and therefore the ingrowth of blood vessels. Purchasing Matrigel that is at least 12–14 mg/ml will reduce this problem. A common problem may be that in certain cases the plug may be hard to see when harvesting. If this happens it may be best to take the larger area of skin around the injection site for histology. It should be noted that fixation can affect some antibody epitopes therefore, frozen sections may also be considered. HistoGel (Thermo Scientific) is recommended for preservation of the fragile plug before fixation. Cracks or bubbles in the matrix may require selection of areas in the plug that are clear of these artifacts.

An adaptation of the assay is an easy to use kit: DIVAA, Directed In Vivo Angiogenesis Assay, Trevigen Inc., that avoids many of the injection and artifact problems [392].

### 18.4 Concluding remarks

Among the various in vivo angiogenesis assays, the plug assay is the most widely used because it is the most versatile, least costly, and easiest to perform. Furthermore, results are available within a week. Care must be taken at all steps in the assay, including in the selection of the recipient mice, handling and injecting the Matrigel, harvesting the plug, and counting the vessels.

## 19 In vivo vascular network forming assay

**Fig. 16:**
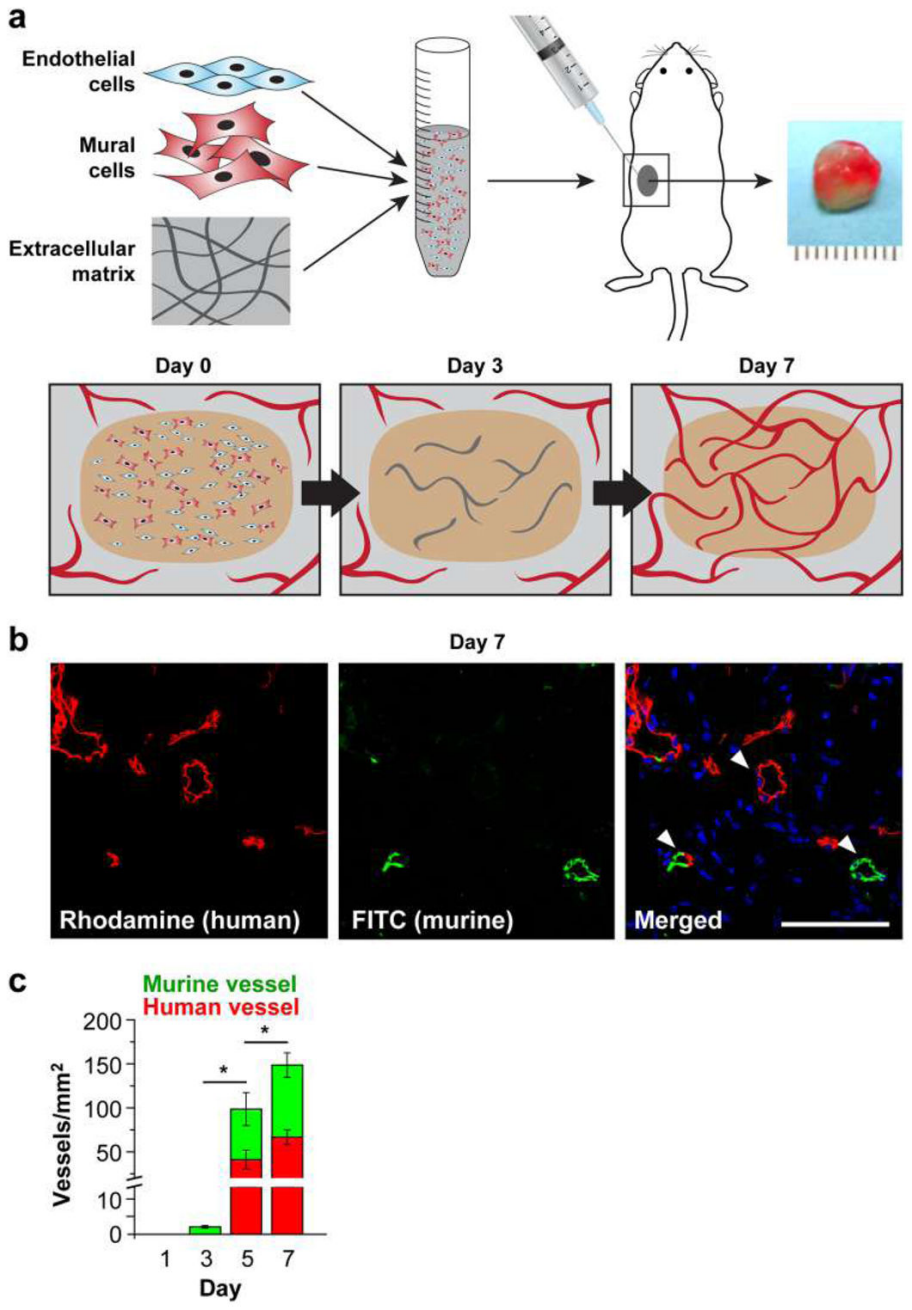
In vivo vascular network formation assay. **a** Schematic of the two-cell model. **b** Tail vein injection of rhodamine-UEA-I and FITC-GS-B_4_ labels perfused human (red) and murine (green) vessels on day 7 after cell/Matrigel subcutaneous injection. **c**) Quantification of lectin-labeled human and murine vessels shows that perfused human vessels present at day 5 and day 7

In this assay, human endothelial cells (ECs) and mesenchymal cells are combined to form perfused vascular networks as rapidly as 4 days after subcutaneous injection into immune-deficient mice. Thus, this assay provides an in vivo system to study human blood vessel formation and function with the option to manipulate the human cells in vitro prior to implantation. Briefly, human ECs are suspended in a liquid extracellular matrix (ECM) or hydrogel with supporting mesenchymal cells and the mixture is then subcutaneously injected into immune-deficient mice (Fig. 16a). The selected ECM or hydrogel should be compatible with vascular morphogenesis and should ideally polymerize soon after injection to confine the cells. The vascular networks formed within the implant are lined with the human ECs and surrounded on the abluminal side by human perivascular cells [393–396] (Fig. 16b). This assay is simple and essentially relies on three key components: (1) human ECs, (2) supporting mesenchymal cells, and (3) hydrogel. Nevertheless, the nature of these three elements can vary considerably, conferring wide versatility to the assay. For example, the human ECs can be derived from progenitor cells (e.g., endothelial colony-forming cells (ECFCs) isolated from umbilical cord blood or adult peripheral blood), from primary tissues (e.g., human umbilical vein ECs (HUVECs), human dermal microvascular ECs, or white adipose tissue-derived ECs), or from differentiated human embryonic or induced pluripotent stem cells [393, 394, 397, 398]. The supporting mesenchymal cells may originate from a plethora of sources as well, including progenitor cells (e.g., mesenchymal progenitor or stem cells (MPCs, MSCs) from bone marrow, cord blood [394, 399], or adipose tissue [400]), and primary cells (e.g., saphenous vein smooth muscle cells (HSVSMC) [393] and human dermal fibroblasts [401]). Moreover, murine sources of mesenchymal cells can alternatively be used, including MSCs from bone marrow, white adipose tissue, skeletal muscle, and myocardium [402] as well as the murine embryonic mesenchymal precursor 10T1/2 cell line [403]. Lastly, this assay is compatible with a variety of ECMs and hydrogels that support formation of human vascular networks including Matrigel [393], Type I collagen [395], fibrin [395], PuraMatrix [395], and photo-crosslinkable methacrylated gelatin (GelMA) hydrogels [404]. In summary, this two-cell model for building human vascular networks in the mouse is versatile, rapid, and relatively simple to perform.

### 19.1 Background/history

Seminal studies by Schechner and colleagues [405, 406] set the stage for the assay described above. They suspended HUVECs in collagen/fibronectin gels wherein the HUVECs formed tubular structures in vitro within 20 h. They then placed the cell-laden gel pieces by incision into the abdominal wall of severe combined immune deficiency (SCID) mice. Human EC-lined vessels were detected in the implants by 30 days, although the investigators found that HUVECs needed to be transduced with the anti-apoptotic gene Bcl-2 to achieve meaningful survival. After 60 days, basement membrane deposition, EC-EC junctions, recruited host α-smooth muscle actin + mural cells, and TNF-α induced E-selectin and VCAM-1 were evident [406]. In summary, HUVECs formed well-developed, functional vessels, albeit slowly. The next logical step was to supply mural cells 1) to avoid reliance on host mural cell recruitment and 2) as a source of growth factors for the implanted ECs. In this regard, including the murine mesenchymal precursor cell line 10T1/2 was shown to significantly increase vessel density beginning at day 14 after implantation [407]. In summary, these early studies represented a “tissue-engineering” proof-of-concept that collectively demonstrated that pre-assembled human vascular networks transplanted into mice were able to connect with host vessels.

### 19.2 Assay overview

We adapted these concepts to develop a rapid, simple, and non-surgical approach for the vascular network forming assay. Our prototypical assay consists of 2 × 10^6^ human ECFCs + human bone marrow-derived MSCs combined at a ratio of 2:3 and suspended in 200 microliters of ice-cold Phenol Red-free Matrigel for each implant. The cell/Matrigel suspension is then injected subcutaneously using a 26 gauge needle; two implants are placed on each dorsal side of 6–8-week-old athymic nu/nu mice. Soon after implantation, Matrigel forms a gel at 37 °C such that cells are confined at the site of injection. Perfused vessels are detected as early as day 4, have a robust presence by day 7 (~ 100/mm^2^), and are long lived [394, 396]. Inclusion of 1 μg/ml basic fibroblast growth factor (bFGF) in the cell/Matrigel suspension significantly increases the number of perfused vessels detected on day 4 [408]. Negative controls usually include injecting the ECM or hydrogel without cells or with ECs alone. Detailed descriptions of the methods including endothelial and mesenchymal cell isolation procedures are provided in these publications from our laboratories [95, 394, 408]. Also, a detailed video protocol of this assay is available [409].

### 19.3 Visualizing and quantifying vessels

At desired time points, the cell/hydrogel implants are removed by making a dorsal incision adjacent to the implantation site to expose the subcutaneous space. The implants are 3–5 mm in diameter and easily distinguishable from surrounding subcutaneous adipose tissue by shape, consistency, and pink/red (vascularized) color (Fig. 16). Digital photographs provide initial data on the macroscopical aspect of the implants. The excised specimens are then fixed in 10% neutral buffered formalin overnight, followed by paraffin embedding and sectioning (5–7 μm sections) for histological analysis. Hematoxylin and eosin (H&E) staining is used to identify red blood cell-containing lumens; empty lumens can be counted separately to track non-perfused vascular tubes [396]. This analysis will capture human vessels as well as host vessels that have sprouted into the gel to form connections with the human vessels [410]. Typically, vessels are counted in ten randomly selected areas from two H&E-stained sections, ~ 150 μm apart, for a total of 20 areas for each implant. Ideally, counters should be blinded to the experimental groups to reduce potential bias. Image analysis software such as ImageJ can also be used to facilitate the counting. As noted above, this assay typically produces a total vessel density between 50 and 150 vessels/mm^2^.

### 19.4 Distinguishing human from murine vessels

Three strategies have been used to visualize and quantify human versus murine blood vessels in the implant. The first is to use human-specific antibodies such as the monoclonal antihuman CD31 antibody (clone JC70A, Dako, Agilent) that has been shown by numerous groups to specifically label human vessels in histological sections. The second approach is to genetically label the human ECs prior to implantation with green fluorescent protein or an equivalent fluorescent marker using lentiviral or retroviral constructs. The supporting human mesenchymal cells can be labeled with a different fluorescent marker for dual in vivo tracking [394]. The third approach is to use plant lectins—*Ulex europaeus agglutinin I* (UEA-I) which is specific for human endothelium and *Griffonia simplicifolia* isolectin B_4_ (GS-B_4_), which binds to mouse endothelium but has no reactivity with human cells because humans lack the α-linked galactose residue needed for GS-B_4_ carbohydrate binding. For instance, tail vein injection of rhodamine-labeled UEA-I and FITC-labeled GS-B_4_ 10 min before removing the implants has been shown to effectively label perfused blood vessels and shows the connections between human and mouse vessels within the grafts [410] (Fig. 16b). Using this lectin approach, three distinct patterns can be seen: UEA-I-positive human vessels, GS-IB_4_-positive murine vessels, and UEA-I/GS-IB_4_-double-positive chimeric vessels; each can be quantified and reported as vessels/mm^2^ (Fig. 16c) [410–412]. Alternatively, biotinylated forms of UEA-I and GS-B_4_ can be used to stain the vessels in histological sections.

### 19.5 Longitudinal analyses

Vascular volume within the implants can be measured longitudinally by contrast-enhanced ultrasonic imaging using a high-resolution system such as the Vevo 2100 (Visual-Sonics Inc, Toronto, Canada) [396, 410]. This technique requires imaging before and shortly after tail vein injection of an echogenic microbubble contrast agent, preferably with a syringe pump to achieve consistent levels of contrast among the different animals. In addition, to assess cell retention over time, the human ECs can be genetically modified for luciferase expression (e.g., Lenti-pUbiquitin-firefly luciferase-GFP); this allows visualizing the location of the human ECs over time in individual mice [394, 411, 412]. This procedure simply requires an intraperitoneal injection of the substrate luciferin followed by live bioluminescence imaging 30–40 min later using a system such as the Xenogen IVIS 200.

### 19.6 Advantages

This in vivo vascular network model is ideally suited for studies on the cellular and molecular mechanisms of human vascular network formation. The assay is amendable to both loss-of-function and gain-of-function experiments. For example, specific genes of interest can be tested by carrying out shRNA/siRNA knockdown or CRISPR/Cas9 gene editing in the ECs and/or the mesenchymal cells prior to implantation. Conversely, specific genes can be expressed using retroviral or lentiviral constructs, providing a gain-of-function test strategy. Alternatively, the human cells can be treated with drugs prior to implantation, which restricts drug exposure to the cell of interest and avoids confounding systemic effects. These types of experiments have been used to study human vascular tumors and vascular malformations [413–415]. Furthermore, Melero-Martin and colleagues showed that human ECs can be genetically engineered to provide controlled release of a protein of interest from the newly formed human vascular networks. In their study, human ECFCs that were genetically engineered to overexpress erythropoietin (EPO) were shown to form vascular networks that increased erythropoiesis and corrected anemia in the recipient mice [416], revealing the engineered network’s potential to serve as a drug delivery device. An additional advantage is that the human ECs and mesenchymal cells, as well as host cells, can be easily retrieved from the implant at any desired time point and sorted into purified populations for molecular and biochemical analyses, or for transplant into secondary, recipient mice. Finally, it is worth noting that hydrogel suspensions of human ECs and mesenchymal cells have also been shown to form vascular networks in murine ischemic myocardium and ischemic hind limbs, providing physiological benefit to those tissues [411, 412]. This demonstrates that the blood vessel-forming ability of the cells can be recapitulated in settings beyond the subcutaneous implant site.

### 19.7 Limitations and challenges

Implanting human vascular cells necessitates the use of immune-deficient recipient mice, which precludes the use of many available transgenic murine models. The use of murine cells would solve this, although obtaining robust and phenotype-stable murine ECs remains a challenge. Murine mesenchymal cells are less problematic; murine MSCs from four different tissue sources have already been shown to support vessel formation in vivo [402]. Therefore, it should be feasible to switch to syngeneic murine cells for implantation into desired murine strains. Second, this assay is not well suited for high-throughput studies because of the relatively large number of human cells required, the expense of immune-deficient mice, and the time-consuming analytical procedures. Alternatively, in vitro 3D microfluidic models in which perfusable human vascular networks form can provide a valuable intermediary between this in vivo assay and 2D cell culture models [417].

### 19.8 Concluding remarks

This in vivo vascular network forming assay is technically simple, reliable, and experimentally accessible; it provides a functional test for assessing the blood vessel-forming potential of human ECs in vivo. The assay allows wide versatility in the use of cell sources. The human cells can be manipulated genetically and/or pharmacologically prior to implantation to address a variety of questions. Human (and murine) cells can be retrieved from the implants at desired time points for cellular and molecular analyses. The assay is also amendable to the use of human ECs from patients or ECs genetically engineered to express a human mutation to model specific vascular pathologies.

## 20 Developing mouse retinal vasculature—tip cells

The developing mouse retina is an extensively used model to study angiogenesis. Vessels originate around birth from the optic nerve and grow radially to the peripheral retinal margin during the first postnatal week as a flat and easily imaged plexus. Subsequently, the superficial capillaries start sprouting downwards to form the deep and intermediate vascular plexus. The network also undergoes some remodeling, and in approximately the third postnatal week, all vascular layers are completely mature. Several aspects of vascular development can be studied in this system, such as endothelial tip/stalk behavior, sprout anastomosis, lumen formation, vessel pruning, network remodeling, formation of arteries and veins, and others. The model is particular useful for the analysis of genetically altered mice.

### 20.1 Visualization of cellular markers and gene expression

To appreciate spatial relationships and network topology, the retinal vasculature is best studied in retinal whole-mount preparations. This, however, contains some challenges. Dissecting the retina in one piece requires practice. Furthermore, depending on fixation conditions and antibodies used, immunohistochemistry can be difficult. The likely reason for this is the formation of diffusion barriers at the retina–vitreous interface. Two fixation protocols have emerged minimizing these problems. The first approach uses fixation for just a few minutes in paraformaldehyde followed by a longer incubation in ice-cold methanol (which can also act as storage medium) [418]. Alternatively, paraformaldehyde fixation is carried out for several hours on ice. Different antibody stains may behave differently depending on the fixation method used. Furthermore, in situ hybridization (ISH) can also be used on retinal wholemounts. Here, care needs to be taken to prevent the retina from curling up during the hybridization step [419]. By far the most simple and robust method of staining the retinal vasculature is based on labeled isolectin B4 (IB4), which binds a carbohydrate epitope on mouse endothelial cells, but also on microglia and macrophages.

### 20.2 Analysis of tip cells

**Fig. 17:**
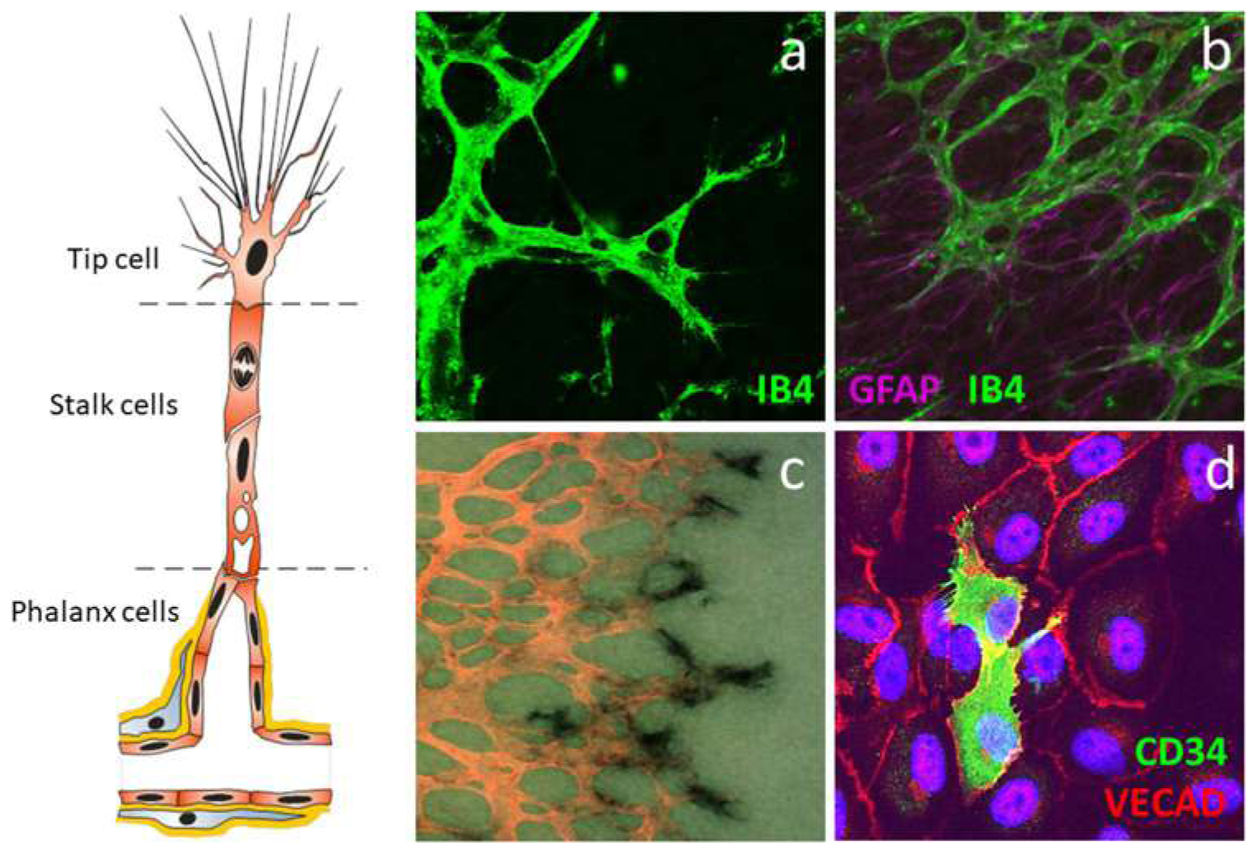
Identification of tip cells. The tip cell is the leading cell of an angiogenic sprout with long filopodia extensions, followed by stalk cells that proliferate and phalanx cells that form a matured new capillary. **a** Tip cells in the developing mouse retina can be identified by staining with isolectin B4 (IB4). **b** The mouse retinal vasculature (IB4) follows the astrocytic meshwork (GFAP) when forming the superficial vascular plexus during development. **c** A combination of collagen type IV IHC (in pink) for general staining of blood vessels and ISH for PDGFB (black) for specific identification of tip cells in the developing mouse retina. **d** Tip cells in endothelial cell cultures are identified by CD34

Endothelial tip cells are defined by their position at the tips of vascular sprouts (Fig. 17). They also have long and dynamic filopodia, migratory behavior, and low proliferative activity. On the other hand, stalk cells, following the tip cells, proliferate and form the vessel lumen. Manipulations that change the balance between the tip and stalk phenotype alter branching frequency of angiogenic sprouts. The basis for this concept was set by Gerhardt et al. [420], who used lectin labeling (Fig. 17a, b) and ISH against PDGFB (Fig. 17c) or VEGFR2 to identify tip cells. The Notch ligand DLL4 plays an important role for maintaining the tip cell state and is also a tip cell marker. In the search for additional tip cell-specific markers, whole genome genetic profiling strategies were used [421, 422] and many other useful markers have emerged such as CD34, apelin (APLN), angiopoietin-2 (ANGPT2), chemokine receptor type 4 (CXCR4), and endocan (ESM1) [419–423]. ESM1 is probably the most specific tip cell marker in mouse retinal vasculature, labeling additionally only some arterial ECs, whereas labeling of CXCR4 is high in tip cells, but also to a lesser extent present in some EC of the vessel plexus, in arterial EC and in perivascular cells [423].

The actin cytoskeleton provides a driving force for tip cell movement during angiogenesis, and labeling F-actin with phalloidin [420] is another way of assessing endothelial tip cells, especially by highlighting and counting their filopodia. As an indirect method to distinguish tip cells from other EC phenotypes, labeling with antibodies against the adherens junction protein VE-cadherin, the adhesion protein PECAM1/CD31 or against proteins in the basal lamina (fibronectin, collagen, laminin) is used in combination with IB4, in which all ECs are labeled, but tip cell filopodia exclusively with IB4 [420, 424]. The use of markers of the basal lamina is based on the concept that formation of new sprouts requires degradation of extracellular matrix to allow migration of tip cells, thus showing reduced staining in proximity of tip cells. Indeed, it was recently shown by triple labeling of tip cells with F-actin, cortactin, and collagen type IV that tip cells use so-called podosomes to degrade the extracellular matrix [425]. Labeling with anti-Ki67 or BrdU [420, 424] is used as marker of proliferation, a property that is greatly reduced in tip cells.

Several transgenic mouse models are also available today to specifically study tip cells in the mouse retina. Lifeact-EGFP labels actin associated with cell–cell junctions, and apical and basal membranes, and highlights actin-based structures such as filopodia and stress fiber-like cytoplasmic bundles [426]. Also, a Cre transgenic strain was developed (*Esm1*-*CreERT2*) [423], mimicking tip cell-specific expression of ESM1 in the retina [421]. Combining this transgenic strain with the *R26*-*mTmG^T/^*^+^ Cre reporter generated, a conditional knockout strain that can be cross-bred with global knockout strains for tip cell genes as *Cxcr4*, or stalk cell-specific genes as *Notch1*, and *Jag1* to study tip cell fate [423]. Furthermore, mosaic mutant mice can be generated by injecting morulas or blastocysts of mice with DsRed or eGFP expressing embryonic stem cells that have undergone gene targeting. The ability of the mutant embryonic stem cells to contribute to EC in the tip position is a direct readout of the importance of the mutated gene for EC tip cell function. Mosaicism can also be achieved by using very low amounts of tamoxifen in tamoxifen-inducible, endothelial specific Cre transgenic strains. Interestingly, even heterozygous gene deletions, with no phenotype in globally targeted mice, can result in a competitive disadvantage in a mosaic background, resulting, for example, in a reduced proportion of mutant EC in tip position [427, 428].

Tip cell behavior can also be studied in ex vivo and in vitro models, which have the advantage over in vivo models that they are suitable for an array of interventions and readouts and allow a rapid and low-cost screening of potential anti-angiogenic molecules. These models, such as retinal or dorsal aorta explants, and EC-coated beads embedded in fibrin gels are well suited to study tip cells and are described in detail in other chapters of this article. It has recently also been demonstrated that tip cells can be identified in EC monolayer cultures such as primary HUVECs, HMVECs and in immortalized endothelial cultures by staining for CD34, an endothelial and hematopoietic stem cell marker, which stains the tips of vascular sprouts but also the luminal side of all EC in vivo [429]. In contrast, in vitro CD34 reliably and specifically stains endothelial cells with almost all characteristics of tip cells in vivo (Fig. 17d). This in vitro model of tip cells allows studies into the differentiation and regulation of tip cells, as well as the discovery of novel tip cell genes [422].

### 20.3 Network analysis of retinal vasculature

The size and topology of the developing retinal vasculature can be used as a general readout to assess angiogenic processes. The overall diameter or the plexus covered during the initial expansion phase of retinal vasculature development is widely used as an indicator of decreased or increased angiogenesis. Moreover, branching frequency, branch length, and vessel diameter are strongly affected by tip/stalk imbalances and can easily be assessed by image analysis (e.g., using tools in ImageJ or Fiji) [430]. The plexus central to the leading front is unstable and remodeled via the pruning of capillaries, leading to reduced vessel density. In addition, capillary free spaces form in the vicinity of arteries. These readouts can indicate changes in vessel stability [431]. Furthermore, artery–vein differentiation is particularly noticeable in the developing retinal vasculature because of the radially alternating patterning of arteries and veins. Arteries are easily labeled with an antibody against alpha smooth muscle actin, which is strongly upregulated in arterial smooth muscle cells. Endothelial specific arterial (e.g., ephrin B2 or DLL4) and venous markers (e.g., Eph B4) can also be used.

### 20.4 Limitations and challenges

Retinal whole-mount IHC and ISH can be tricky, and the accessibility of antibodies in the retina may be dependent on the protein or mRNA targeted. Transgenic reporter mice partly overcome these problems greatly reducing background and show a more equally distributed expression, but are not affordable for every laboratory. A further limitation to the use of tip cell markers is that none of the current markers exclusively labels tip cells, as they can also be expressed in other EC of the vascular plexus, arteries, or perivascular cells. Therefore, identification of tip cells relies mainly on their position, morphology, and their filopodial protrusions. To confirm the endothelial identity of tip cells, staining with multiple antibodies is necessary to distinguish between EC and perivascular cell types.

Another major limitation of the developing mouse retinal vasculature as a model system is that experimental manipulations are challenging. Although intraocular injections are possible, they are prone to artefacts and requires highly skilled and experienced operators. Genetic models are more robust but usually require an inducible approach (e.g., the tamoxifen-inducible Cre-lox system) because the retinal vasculature develops postnatally and cannot be studied when vascular abnormalities lead to embryonic death. The associated mouse breeding can be very costly and time-consuming. Thus, ex vivo and in vitro assays may be in many instances more appropriate as they are faster, cheaper, and ethically more responsible. However, more complex network development depends on in vivo approaches and can so far not be studied in vitro.

### 20.5 Concluding remarks

The developing retinal vasculature is a very well-defined and widely used model to assess vascular phenotypes in mutant mice. It has been particularly useful in the study of endothelial tip/stalk behavior in physiological context. However, inducible genetic tools are usually required and the associated mouse breeding makes it a resource and time-intensive model.

## 21 Corneal angiogenesis assays

In the early 1970s, the Folkman’s group firstly set up an in vivo angiogenesis by exploiting the anatomical features of rabbit cornea and in particular the absence of blood vessel and the easy access to monitor the neovascularization by a slit lamp after implanting tumor cells or tissues or slow-release pellets containing angiogenic inducers [432]. This strategy was further extended to rodents (mouse and rats), which are smaller and less expensive than rabbits and allow studying specific molecular correlations by taking advantages of using genetic engineered mouse models [433–435]. Corneal assays are instrumental to characterize angiogenic inducers and inhibitors, interactions between different factors, and to study cellular and molecular mechanisms of angiogenesis [436]. Interestingly corneal assay can also be used to study other biological processes. For example, studies of lymphangiogenesis were made possible through the implantation of low-dose bFGF pellets, which allowed the visualization of lymphatic vessels through specific molecular markers [437].

### 21.1 Assay overview

The protocol is based on the creation of a small pocket in the cornea to introduce tissue samples, cells, or slow-release pellets incorporating growth factors. Surgical procedures are performed on anesthetized animals, in sterile conditions to avoid inflammatory reactions and with the help of a stereotaxic microscope. Generally, the resins used are etylenvinyl acetate (Elvax 40W, 40% by weight ethylene–vinyl acetate comonomer content with a “W” amide additive, Dupont) or a mixture of sucralfate and poly(2-hydroxyethyl methacrylate) (Hydron). To produce slow-release Elvax 40 pellets, 1 g of the polymer is washed in 100 ml of absolute alcohol for at least 15 days and then dissolved in 10 ml of methylene chloride to prepare 10% casting stock solution (60 min in at 37 °C). Two hundred microliters of this solution is layered on a Teflon film, and the methylene chloride is allowed to evaporate under a laminar flow hood. The resulting film of polymer is cut under a stereomicroscope into 1 × 1 × 0.5 mm pieces, which are used as implants. Ten implants (see above) are monitored for 14 days after implantation. The casting solution is eligible for the use when the implants performed with a specific casting solution do not induce reactive inflammatory injury at microscope and histological examination of the cornea. To incorporate the test substance, a predetermined amount of casting solution is mixed with a given amount of the molecule and processed as above described. Alternatively, pellet is incubated with a solution of the testing molecule dissolved in sterile phosphate buffer solution for 40 min at room temperature in a plate rotator and then dried in a laminar airflow hood under sterile condition.

Elvax-40 containing testing molecules can be stored up to 1 month at −80 °C. Empty pellets of Elvax-40 are used as negative control, while positive control contains VEGF-A (200 ng/pellet). In the case the experiment protocols require the use of cells or tissues, it is used to implant 5 μl of medium containing 2–5 × 10^5^ cells, or a fragment of 2–3 mg of fresh tissue.

A second resin is hydron stabilized by sucralfate. Ten milligrams of sucralfate is added to solutions of sterile saline (20–50 μl) containing the appropriate amount of testing substances (e.g., 0.05 mg VEGF-A as positive control) that are then speed vacuumed until the mixture is dry (30–60 min). To this suspension, 10 μl of 12% hydron in ethanol is added. This suspension is layered on a sterilized 1.5 × 1.5 cm piece of nylon mesh (pore size 0.4 × 0.4 mm). Both sides of the mesh are covered with a thin layer of hydron and allowed to dry in sterile conditions. Then, the fibers of the mesh are pulled apart under a microscope, and only uniformly sized pellets of 0.4 × 0.4 × 0.2 cm are selected for implantation. All procedures are performed under sterile conditions. Such pellets can be stored at −80 °C for 1 month without loss of bioactivity.

Pellets are implanted in anaesthetized animals, and the eye is further anesthetized with few drops of local anesthetic. A central incision (1.5 × 3 mm in rabbit; 1.5 × 1.5 mm in mouse or rat) is produced into the corneal 3 mm (rabbit) or 1–1.5 mm (mouse, rat) from the limbus, and using a von Graefe knife (2 × 30 mm), a micropocket is dissected to insert the pellet with forceps. This procedure allows the diffusion of test molecule in the tissue, with the formation of a gradient for the ECs of limbal vessels. The distance of the pocket from the limbus can influence the angiogenic response, which depends on the features of the angiogenic inducer [438].

Observations of the implants are made with a slit lamp stereomicroscope over a set period of time on anaesthetized animals. Images are recorded for subsequent analysis. In rabbit, an angiogenic response is positive when budding of the vessels from the limbal plexus occurs after 3–5 days and capillaries progress to reach the implant in 7–10 days. Implants with an inflammatory reaction or that are unable to induce angiogenesis within 10 days are discarded. The angiogenic activity is evaluated on the basis of the angiogenic score calculated by the formula vessel density/cornea × distance from the limbus [439, 440]. The number of positive implants over the total number is also scored.

In the mouse or in the rat, the vascular response is measured as the maximal vessel length [441, 442]. By using the slit lamp microscope with an ocular grid, the y-axis of the reticule is located along limbal vessel directly beneath implant. It is possible to refer the eye as a clock with intervals from 1 to 12, and the measurement of the number of clock hours with new vessels is performed during each observation. Two measurements are obtained for each eye: the linear response representing the maximal vessel length (an average of the five longer vessels) extending from the limbus toward the implant and the circumferential response representing the neovascularization zone (measured in clock hours). The vessel area is calculated according to this formula: vessel length × clock hour × 0.2π. At the end of the experiments, cornea is fixed and analyzed for the presence of inflammatory cells and to investigate the vessels features.

### 21.2 Limitations and challenges

Corneal assays present advantages and disadvantages in different species. The rabbit’s size enables an easy manipulation of both whole animal and the eyes, and the inflammatory reactions are easily detectable as corneal opacity. However, the experimental cost is higher in rabbit than in mouse or rat. Multiple observations are easier in rabbit than in mouse or rat and can be done in not anaesthetized animals. Furthermore, the angiogenic response in mouse and rat is highly variable and a large number of animals are required. Thanks to its size, the rabbit eye offers a large area for the placement of stimuli in different forms including the presence of two pellets. All species can be also used to test the effect of local or systemic drug treatment on the local angiogenic response. Obviously, the systemic drug treatment of rabbit is more expensive than the treatment of mouse or rat. There are some critical steps in performing a successful corneal assay. The first issue is the preparation of pellets, which need to be uniform, without any inflammatory activity and able to ensure a good distribution of the angiogenic molecule. Surgical procedure is another challenge issue of the assay, and it is important to calibrate the appropriate depth of the incision to avoid the eye rupture. In mouse, this assay permits to induce neo-angiogenesis on different genetic backgrounds, which can be useful to evaluate the effect of a specific gene on angiogenesis.

### 21.3 Concluding remarks

Corneal angiogenesis assay, which requires high technical competences, represents a powerful tool to monitor and quantify in vivo neo-angiogenesis, to test specific drug treatment and the effect of genetic manipulation. To possibility of final histological examinations allows an initial description of the cellular mechanisms sustaining the process.

## 22 Mouse oxygen-induced retinopathy model

Pathological angiogenesis plays a pivotal role in the most common causes of blindness including neovascular age-related macular degeneration (AMD) [443], polypoidal choroidal vasculopathy (PCV) [444], or diabetic retinopathy and retinopathy of prematurity (ROP) [445]. Animal angiogenesis models are an essential tool to understand pathways involved in physiological and pathological angiogenesis for basic comprehension of the biology and to determine the basis for in vivo anti-angiogenic drug development in translational cancer and ophthalmic research. Moreover, they are critical in preclinical drug testing.

The oxygen-induced retinopathy (OIR) model was originally developed to reflect the pathological stages of human ROP, vaso-obliteration of retinal vessels with oxygen exposure (phase I), followed by vaso-proliferation (phase II). Oxygen exposure of neonatal dogs, cats, rats, and mice creates a relative hypoxic state in the retina with stimulation of growth factors resulting in eventual vaso-proliferation as is seen in preterm infants with ROP [446]. A major distinction of this model is that mice, rats, dogs, and cats are born with incompletely vascularized retinas, similar to humans born preterm allowing the study of retinal vascular development. Mice are the least expensive and most genetically manipulative animal model and therefore the most commonly used. Murine retinal angiogenesis occurs postnatally in a tightly regulated, predictable manner similar to humans allowing for manipulations seen postnatally in preterm babies such as oxygen exposure [445].

Since the introduction of the current mouse OIR model in 1994, it has been extensively used in many studies of angiogenesis and retinopathy. A Google Scholar search with the keywords “oxygen-induced retinopathy mice” revealed approx. 45,000 publications with an increase of 9500 publications in the last year, reflecting the feasibility and practicality of this in vivo model in many areas of vascular biology research. The major advantages lies in its reproducibility, affordable costs, short duration (10 days), and accessibility of the retinal vasculature for isolation, imaging, and interventions [447, 448]. Further, the well-established platforms for creating systemic or conditional transgenic strains and access to a wide availability of recombinant proteins and antibodies make it a powerful tool for basic angiogenesis research.

### 22.1 Assay overview

**Fig. 18:**
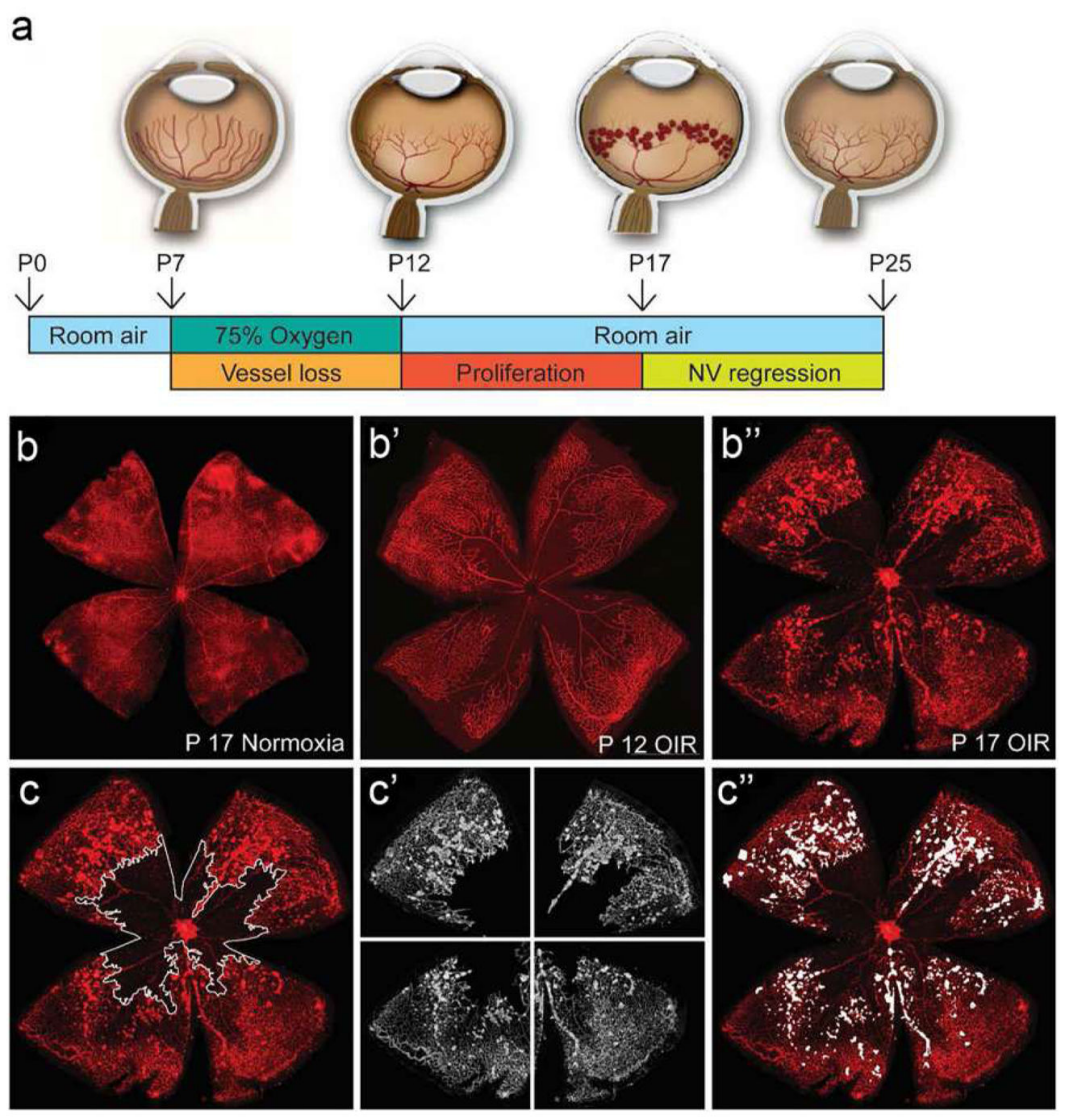
Mouse model of oxygen-induced retinopathy (OIR). **a** The mouse model of oxygen-induced retinopathy (OIR). Neonatal mice and their nursing mother are placed into 75% oxygen from P7 to P12, which induces loss of immature retinal vessels, leading to a central zone of vaso-obliteration (VO). After returning to room air at P12, the central avascular retina becomes hypoxic, inducing vascular regrowth with pathologic neovascularization (NV). At P17, the maximum severity of NV is reached. NV starts to regress shortly after P17 and almost no VO or NV remains visible by P25. **b** Retinal whole mount stained with isolectin-B4-Alexa (red) displaying a normal vascular development at P17 under normoxic condition. **b**′ OIR P12 retinal whole mount showing an extensive VO area without NV. **b**″ OIR P17 retinal whole mount showing a decreased VO with NV at its maximum. **c** Quantification of vaso-obliteration (VO) by manually outlining the avascular area with image-processing software (Photoshop, Adobe Systems) **c**′ For computer-aided NV quantification, both the original image and the VO image generated with Adobe Photo-shop were imported into NIH’s free-access ImageJ software. The SWIFT_NV macroset isolates the red color channel, subtracts background fluorescence, and divides the VO image into four quadrants. **c**″ SWIFT_NV then allows the user to outline NV tufts but not normal vessels by setting a fluorescence threshold for each quadrant. The macroset then quantifies all NV pixels from all four quadrants, reports the result as neovascular total area, and creates an overlay of NV and original image

The current OIR model was developed by Smith et al. [446] and optimized by Stahl et al. in 2009 [449]. In brief, neonatal mice with nursing mothers are placed into 75% oxygen from postnatal day (P)7 until P12 and returned to room air (21% oxygen) from P12 to P17 (Fig. 18a). Due to the high pO_2_ levels during the first hyperoxic phase (P7–P12), retinal vessels stop developing and immature capillaries in the central retina regress resulting in a central zone of vaso-obliteration (Fig. 18a, b′) [450]. Interestingly, the oxygen-induced vaso-obliteration (VO) develops very rapidly with peak VO 48 h after onset of oxygen exposure [446]. The retina starts revascularizing slowly under remaining oxygen exposure from P9 to P12 [446, 451], likely reflecting increasing oxygen demands of the developing retina. On return to room air at P12, the VO area becomes hypoxic [452] and significant upregulation of HIF-1-dependent, pro-angiogenic pathways including VEGF ensues resulting in neovascularization (NV) with its peak at p17 [453–455] (Fig. 18a, b″). A comprehensive step-by-step protocol of this mouse model has been published [456].

The two main quantifiable components in the OIR mouse model are the NV (maximum at P17) and VO areas. If a study is designed to determine the amount of vasculature lost during the hyperoxic phase, the VO area can be measured at P12 or even as early as P8 by manually outlining the VO in an image software such as Adobe Photoshop (Fig. 18c). In most cases, NV and VO are measured at P17 when abnormal vessel growth is at its peak. It has to be kept in mind that the VO area on P17 can differ between experiments with same VO area at p12 depending on the extent of vascular repair, which can be determined by examining the difference between VO at P12 and P17. The current method to quantify NV is the SWIFT_NV method, which is a computer-aided semiquantitative technique detecting NV tufts and clusters on retinal wholemounts using the free NIH software ImageJ [449]. In brief, using different macros, retinal flat mounts are divided into four quadrants and background fluorescence is removed allowing NV structures to stand out clearly (Fig. 18c′, c″). This enables the user to set a fluorescent threshold marking NV, but not normal vessels. Artifacts can be manually excluded during this step. Recently, a novel Web-based image analysis tool (http://oirseg.org/) has been developed based on deep learning segmentation, which greatly facilitates the quantification of NV structures [457]. This method greatly improved efficiency, reliability, and objectivity compared to the prior methods such as cross-sectional techniques and whole-mount grading systems based on predefined characteristics.

### 22.2 Limitations and challenges

Despite standardization of the OIR mouse model over the last 23 years, several essential factors can result in substantial phenotype variability if not controlled. One of these factors is postnatal weight gain (PWG) during the OIR period. A study from Stahl et al. [458] revealed that pups with poor postnatal weight gain (PWG) (defined as pups weighing 5 g or less at P17) have a significantly delayed and prolonged vaso-obliteration and NV compared to medium (5–7.5 g) and extensive (≥ 7.5 g) PWG. Therefore, the weight of each pup at P7 and again at P17 should be measured. Mice below 6 g and over 7.5 g at P17 should be excluded from the analysis. To avert poor PWG during OIR, several adjustments can be considered. We recommend avoiding large litter sizes over eight pups by removing some pups (or if pups are too heavy by adding pups to maintain appropriate weight gain) and surrogating C57BL/6 pups at P2 or P3 with S129 lactating mice due to their lower susceptibility to oxygen toxicity if weight gain is low. It is important to note that nursing mothers are no longer fertile after exposure to OIR. Under hyperoxia exposure and especially after transfer to normoxia, we found that mothers cannibalize their pups. Therefore, we recommend careful handling after return to normoxia as stress leads to less lactation and increased cannibalization.

Another important variable, which needs to be considered, is the phenotype variability between different strains and even within one strain due to vendor-related substrain differences. These differences can be easily addressed by using the same strain from a single vendor. To correct for age and genetic heterogeneity as well as environmental differences, the use of littermate controls is strongly recommended. When using transgenic mice, controls with exactly the same background will help to minimize strain-dependent phenotype variability.

Last, it has to be noted that the OIR mouse model does not cover all aspects of human ROP since mice are not born preterm even though retinal development occurs postnatally as it does with preterm infants. Thus, systemic factors resulting from preterm delivery are not taken into account, such as low IGF-1 seen after preterm birth in humans. Also, mice have no macula (the area of acute central vision) and have fewer cones than humans. However, vascular development is very similar.

### 22.3 Concluding remarks

The OIR mouse model is a robust, reliable, and quantifiable model to investigate developmental and pathological angiogenesis when variables are carefully controlled.

## 23 Laser-induced choroidal neovascularization mouse model

Age-related macular degeneration (AMD) is one of the major causes of vision impairment in the elderly [459]. Although AMD does not lead to complete blindness, loss of central vision makes it difficult for patients to recognize faces, drive, or read. Nonexudative or non-neovascular AMD includes early and intermediate forms of AMD, as well as geographic atrophy, in which progressive loss of retinal cells leads to some loss of visual function. In 10–20% of AMD patients, nonexudative AMD progresses to neovascular AMD, which accounts for ~ 90% of AMD-associated vision loss with deterioration of central vision [460]. Neovascular AMD is characterized by choroidal neovascularization (CNV), with blood vessels from the choriocapillaris penetrating through Bruch’s membrane into the normally avascular subretinal space [461]. However, in some patients, pathological angiogenesis develops from retinal vessels known as retinal angiomatous proliferation (RAP). In vitro EC culture models of CNV lack complex in vivo cellular interactions of photoreceptors, retinal pigment epithelium, pericytes, inflammatory cells, and glial cells [462]. Therefore, reproducible animal models that mimic the pathological angiogenesis in the retina and choroid are needed to study AMD [463]. No models, however, mimic the aging aspects of the disease, nor do non-primates have a macula.

### 23.1 Laser CNV model advantages and limitations

A laser-induced in vivo model of CNV, first described in 1979, uses photocoagulation to disrupt Bruch’s membrane, inducing the growth of new choroidal vessels into the sub-retinal area [464]. This model is similar to the majority of neovascular AMD in which pathological angiogenesis arises from the choroid. However, it differs from AMD as it is a wounding model unlike neovascular AMD that is initiated with aging changes. The laser-induced CNV model has been used successfully to predict the clinical efficacy of anti-vascular endothelial growth factor (VEGF) therapy to suppress neovascular growth in AMD [465]. Laser-induced CNV has been used in mice, rats, rabbits, pigs, and non-human primates. Non-human primates’ retinal anatomy (with a macula) is most similar to humans, but primates are costly to maintain, and rarely develop AMD and only do so after decades. Rodent models offer the advantages of lower cost but lack an anatomical macula [466]. Several gene-targeted mouse models, such as the very low-density lipoprotein receptor knockout (*Vldlr* −/−), superoxide dismutase (SOD) knockout (*Sod1* −/−), and apolipoprotein E (APOE) e4 transgenic mice, demonstrate some aspects of CNV such as spontaneous subretinal neovascular lesions [462]. However, these models are difficult to further manipulate genetically to determine pathways involved in AMD progression, whereas the laser-induced CNV model can be used in transgenic animals to explore the molecular mechanisms of CNV formation.

C57BL/6J mice are usually recommended for laser-induced CNV experiments, because only pigmented mice absorb laser energy well and respond reliably to laser burns. Previous studies suggest that both gender and age of animals influence the outcome of laser-induced CNV [467]. In one study, mice of both genders more than 12 weeks old developed more severe CNV than mice less than 8 weeks old. Gender difference was only significant in 12–16-week-old mice, but not in the younger 6–8-week-old mice [467]. However, Zhu et al. [468] observed larger CNV lesions in female mice at 5–8 weeks old. This discrepancy may be due to differences in analysis time points and fluorescent methods between the studies. Especially noteworthy, the older female mice developed significantly larger CNV lesions than both older male and younger female mice. The larger area of CNV in older female mice is suggested to be related to their high circulating levels of estrogen, which increases pro-angiogenic functions of both EC and smooth muscle cells in vivo and promotes wound healing in both human and animal models. Moreover, compared with the younger mice, the lesion area in the older mice had increased variability. These studies suggest that mice of either gender weighing 15–23 g at 6–8 weeks of age are optimal for the laser-induced CNV model for testing efficacy of drugs, although age- and gender-matched mice may be essential for specific experiments.

### 23.2 Assay overview

**Fig. 19:**
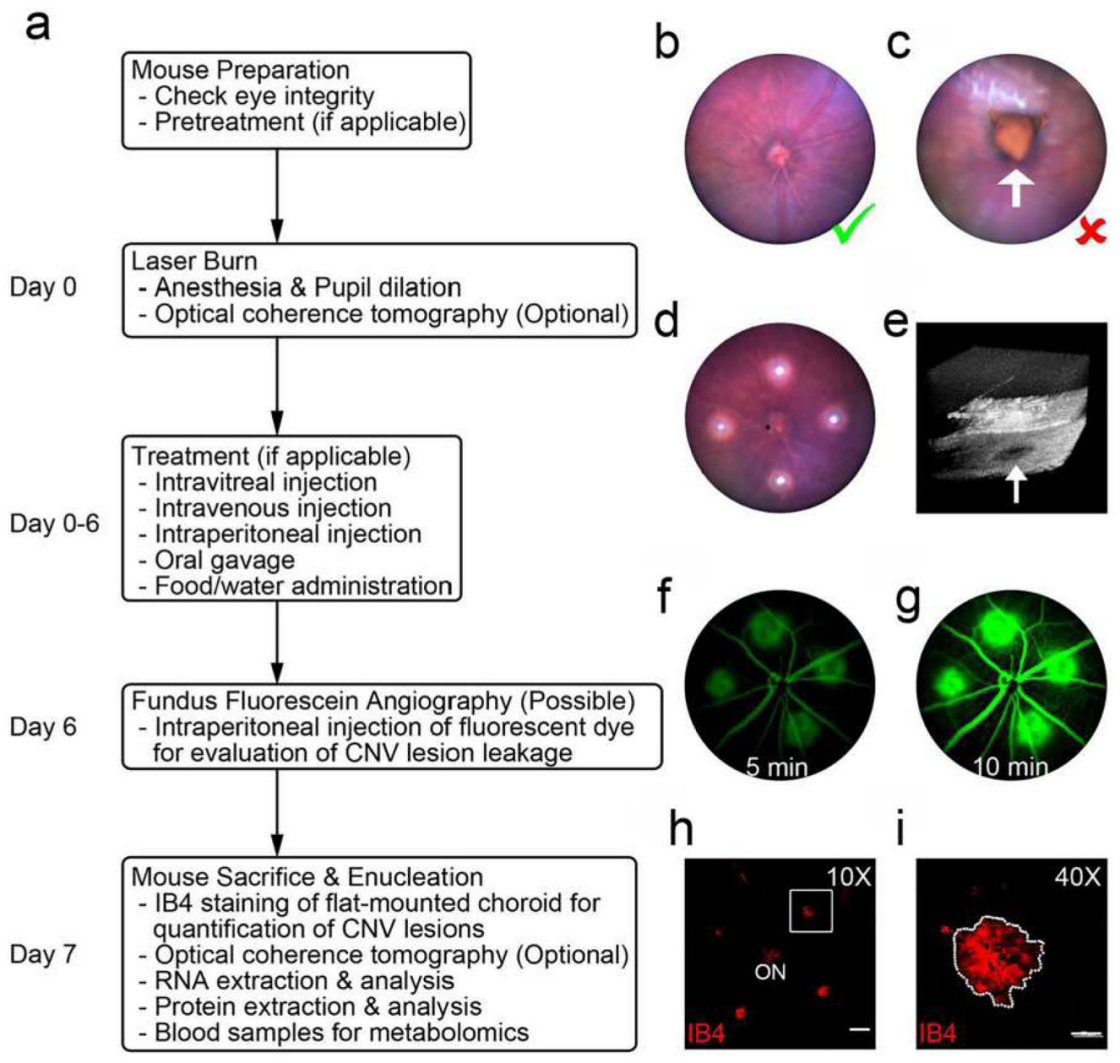
Experimental flowchart of the image-guided laser-induced CNV model and data collection. **a** Overview of the procedure for CNV induction involving mouse preparation and followed by experimental treatment, sample preparation, and analysis. **b** Representative image of normal fundus (Green check mark). **c** Representative image of anomalous structure (white arrow) in the eye, which is not suitable for laser photocoagulation (Red X). **d** Representative image of normal fundus with four laser burns shown as bright white spots. **e** Representative image of a successful laser burn (white arrow) with 3D optical coherence tomography. **f**, **g** Representative ocular fundus fluorescein angiography images at 5 and 10 min after the injection of fluorescent dye at day 6 after laser burn. **h** Representative images of flat-mounted choroid with IB4 staining at day 7 after laser photocoagulation. Scale bar: 200 μm. ON: optic nerve. (**i**) Higher magnification of the laser-induced CNV lesion highlighted in panel H. Scale bar: 50 μm. Reproduced with permission from [467]

The general procedure of laser-induced CNV induction involves careful mouse anesthesia, mouse positioning, laser burn, with optional optical coherence tomography and fundus fluorescein angiography, eye dissection, choroid staining and imaging, and CNV lesion quantification (Fig. 19a). Only intact eyes (Fig. 19b) without observable structural or morphological abnormalities are used for the laser-induced CNV model. Eyes with anomalous structures (Fig. 19c), cataract, or visible defects of the cornea or fundus are excluded. Mice are anesthetized with a mixture of xylazine and ketamine, and pupils are dilated with topical drops of Cyclomydril. 2 min after pupil dilation, lubricating eyedrops are applied to the cornea. Four laser burns at equal distance from the optic nerve (which optimally is approximately twice the diameter of the optic nerve) are induced one by one in each eye by a green Argon laser pulse with a wavelength of 532 nm (Fig. 19d). The distance between laser burns must be at least double the diameter of the optic nerve to avoid fusion of lesions. Major retinal and choroidal vessels should be avoided to prevent potential bleeding. The formation of a vaporization bubble immediately after laser photocoagulation indicates the success of a laser burn, which correlates with rupture of Bruch’s membrane. Optical coherence tomography immediately after laser photocoagulation may be used to confirm the success of the laser burn with visible rupture of Bruch’s membrane (Fig. 19e). After laser photocoagulation, the eyes are gently rinsed with sterile saline to remove the lubricating eyedrops and treated with an antibiotic ointment, erythromycin. Mice are then placed on a pre-warmed warming plate at 35 °C after the laser treatment until they are fully awakened. Mice with or without treatment can be subjected to fundus fluorescein angiography to evaluate the levels of vascular leakage from CNV lesions 6 days after laser burn (Fig. 19f, g). The in vivo retinal structure may also be examined by optical coherence tomography, if applicable, to determine the cross-sectional area of CNV lesions 7 days after laser burns. To measure the surface area of CNV lesions, the fluorescence-stained retinal pigment epithelium/choroid/sclera flat mounts are imaged (Fig. 19h, i) and quantified by researchers masked to treatment. The choroidal CNV samples may also be analyzed for RNA or protein.

### 23.3 Limitations and challenges

The laser-induced CNV model in mice has been often characterized as variable and inconsistent. Establishing a set of consistent exclusion criteria is necessary for ensuring reliable data analysis. In a typical study, ten mice per group with four lesions per eye would optimally provide 80 data points for each experimental condition. To account for data or mouse loss, including (1) cataract and corneal epithelial edema before laser photocoagulation, (2) unsuccessful laser burn without Bruch’s membrane rupture, (3) odd lesion shape due to mouse movements during laser induction, (4) death of mice post-laser treatment, or (5) damage of the CNV lesions during tissue dissection and processing, more mice may be needed and should be considered in a power analysis to account for an anticipated intervention effect.

To accurately evaluate the laser-induced CNV, some lesions should be excluded. Severe hemorrhages will cause much larger CNV lesions, whereas choroidal damage will yield a CNV lesion much smaller than the fellow CNV lesions in the same eye. First, choroidal hemorrhages encroaching on the lesion should be analyzed and classified carefully: (1) If the diameter of the bleeding area is less than that of the lesion, the lesion will be eligible for inclusion of analysis; (2) if the diameter of the bleeding area is more than that of the lesion but less than two times of the lesion diameter, the lesion should be excluded from quantification; and (3) if the diameter of bleeding area is more than two times the lesion diameter, all lesions in the same eye should be excluded from analysis. Second, excessive laser burns that damage not only Bruch’s membrane but also the choroid and retinal pigment epithelium should be excluded. These excessive burns can be seen clearly as a solid “hole” in the bright field of choroid imaging. Lesions should also be excluded if (1) the lesion is fused with another lesion, (2) the lesion is either more than five times larger than the mean of the lesions under the same experimental conditions, or (3) the lesion is the only one eligible for statistical analysis among all lesions in an eye. Previous studies provide the optimal settings and conditions to make use of the laser-induced CNV model for the goal of improving the consistency and reproducibility of experimental results for AMD and other pathological angiogenesis research [467].

### 23.4 Concluding remarks

The laser-induced choroidal neovascularization model is an attractive model to study AMD. The model is available in many species and is, despite the fact that the initiation of the pathology is through wounding and not because of aging, suitable for the development of drugs for the treatment of AMD.

## 24 Transparent window preparations for angiogenesis studies in mice

Our ability to observe living tissues with the microscope improved dramatically in the 1920s when Sandison began implanting transparent windows over wounds in rabbit ears. This allowed him to view the underlying tissue noninvasively and longitudinally [469, 470]. In the 1940s, Algire modified Sandison’s method to visualize the dorsal skin in mice [471]. With this powerful tool, he was able to perform detailed studies of angiogenesis during wound healing and tumor growth. Intravital imaging techniques have provided unprecedented insight into tumor vessel formation and function [472–474]. By implanting transparent windows in various anatomical locations in the mouse, it is possible to observe angiogenesis associated with tumor development, wound healing, and immune/inflammatory responses with high resolution, and in the native microenvironment. To date, transparent window preparations in mice have been developed to observe angiogenesis and lymphangiogenesis in the dorsal skin [475], brain [476, 477], cerebellum [478, 479], liver [480, 481], lung [482], pancreas [483, 484], breast [485], and lymph node [486], Table 3.

### 24.1 Development and capabilities

Advances in intravital microscopy have come with the development of new window models, but also with innovations in microscopy. Multiphoton laser scanning microscopes provide high resolution, 3D images of vessel anatomy, gene expression, and network topology, even in deeper regions of tumors [487]. More recently, frequency domain optical coherence tomography (OCT) imaging has been applied in window chambers to improve depth penetration and contrast, providing unprecedented images of vasculature noninvasively and longitudinally [485]. Another imaging technology, short-wave infrared imaging (SWIR), has been adapted from the defense industry to improve imaging depth penetration and reduce scattering [488]. The development of novel probes for improving contrast has also improved the ability to monitor angiogenesis in vivo [489, 490]. Furthermore, advanced imaging technologies such as second-harmonic generation imaging allow us to see important components of the angiogenic process such as ECM fibers [491]. This ability has improved our understanding of the involvement of matrix components in the angiogenic process [491–493].

Chronic window preparations allow analysis of vessel function, including hemodynamics [494–496], immune cell trafficking [497], transport of materials in the vascular and interstitial spaces [472, 487, 498–501], the binding kinetics of drugs in vivo [502], and other microenvironmental parameters such as perivascular pH and pO_2_ [503–505]. Using intravital reporters, it is possible to study spatiotemporal expression of genes [506], to quantify vessel structure, function, and dynamics [485, 493], and to probe the abnormal organization, structure, and function of angiogenic vasculature (e.g., hyper-permeability, heterogeneous, and compromised blood flow) [475, 477, 487, 507, 508]. When combined with sophisticated imaging and analysis techniques, we can quantify blood velocity, hematocrit, and shear rate in vivo [495].

**Fig. 20:**
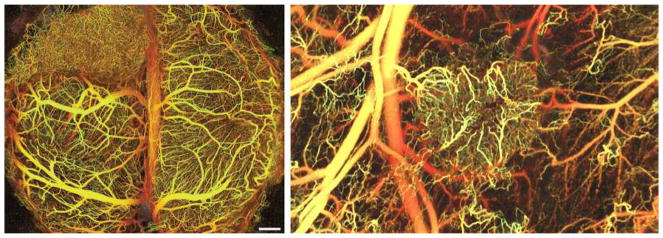
Tumor angiogenesis imaging. Vasculature in the brain (left) and the dorsal skin (right) visualized using IR frequencies to image deeper into tissue; blood flow creates the contrast, so it is noninvasive (from [485])

Intravital microscopy through transparent windows has been used to study many aspects of neovascularization and vessel dynamics (Fig. 20). In addition to tumor angiogenesis and wound healing, they have been valuable for understanding the integration of engineered tissue grafts into existing vascular beds [398, 407]. By following various cell populations as angiogenic host vessels make new connections with those of the graft, it is possible to understand the cellular mechanism of anastomosis [509].

Intravital imaging also allows assessment of therapies that affect the vasculature. For example, it is possible to quantify the abnormalities in structure and function that are hallmarks of tumor angiogenesis, and then assess how these abnormalities change when the vasculature is normalized with anti-angiogenic or anti-fibrotic therapies [478, 500, 508–513]. Intravital optical microscopy [514] has also been useful for calibrating clinical imaging modalities in patients [515].

With information about the structure and dynamics of the angiogenic network, it is possible to analyze performance and efficiency of the vasculature at a deeper level. These analyses allow for further probing of the pathophysiology of tumor angiogenesis and the implications of abnormal vasculature for drug and nutrient delivery [516–519].

**Fig. 21:**
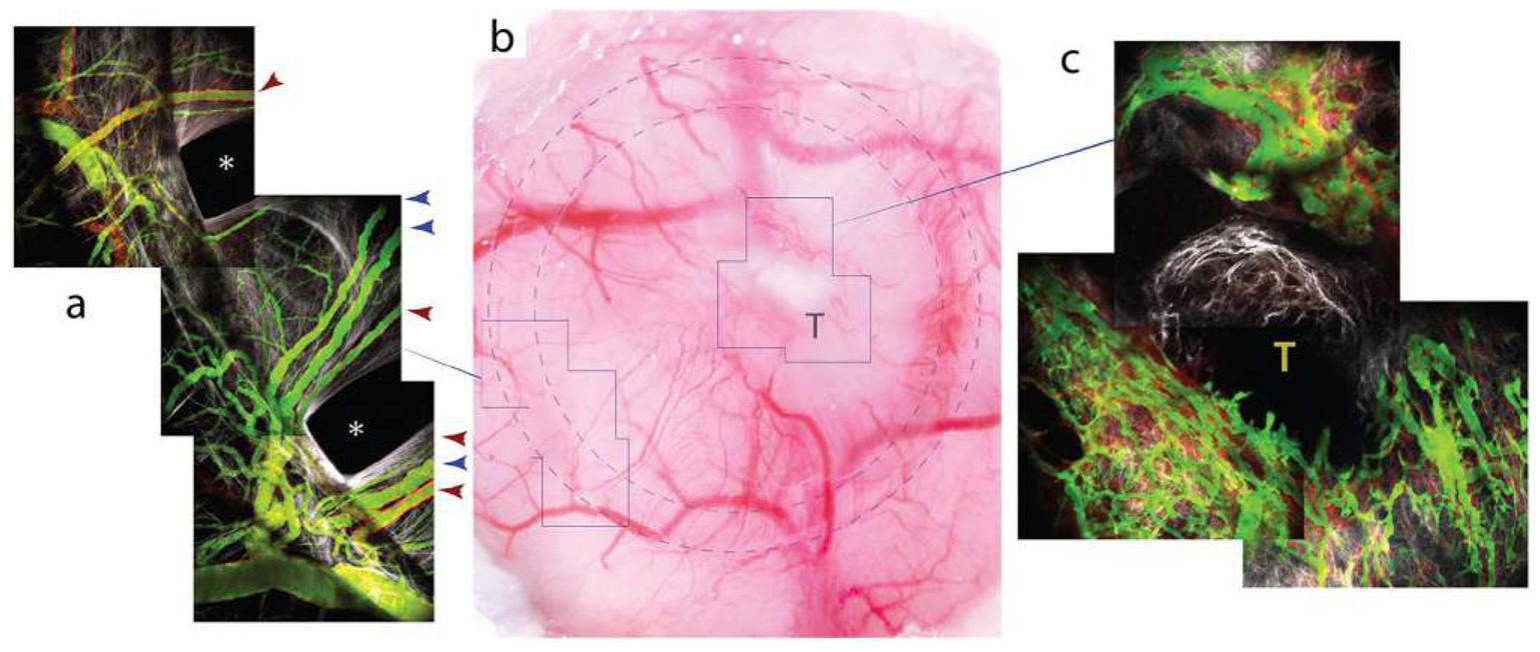
MMTV tumor vasculature in the cranial window pillar TIC. **a** MPLSM/SHG image of the indicated region in the bright-field image (**b**). Near the edge of the PDMS, the vasculature extends radially into the central chamber. At this time point (day 7 after implantation), the vasculature is mature and has normal morphology in the regions far from the tumor. Four feeding arterioles (red arrowheads) and three venules (blue arrowheads) are indicated. These vessels have significant flow and have acquired smooth muscle cells in their walls (red, *αSMA*^+^-*DsRed*). Note that the arterioles generally have more *α*SMA signal than the venules, as expected. **c** The vasculature near the growing tumor has dramatically different morphology and flow, as observed in other animal models and human tumors. The tumor was not fluorescently labeled in this group, but is visible as the mass extending from the central tumor (T) in **b** (from [493])

Microfabrication technologies popularized in the past two decades have allowed the design and implementation of more sophisticated chamber designs. By creating custom-designed window chambers from polydimethylsiloxane, a clear, biocompatible polymer, it is possible to control the interactions between host and implanted tissues and to improve imaging by partially constraining the growing tissues [493] (Fig. 21).

### 24.2 Advantages of intravital microscopy/chronic window chambers for angiogenesis studies

**Fig. 22:**
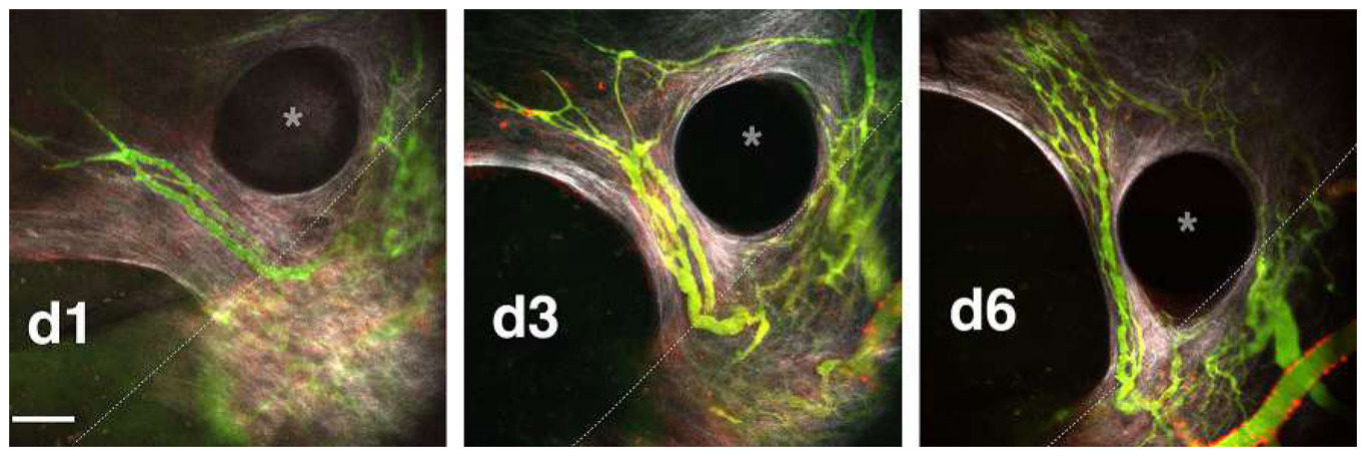
Imaging of vasculature in the cranial window preparation. Vascular sprouts entering a cranial window tissue isolation chamber. Time sequence of new vasculature (green) migrating toward the top left into a cranial window TIC, past the edge of the PDMS disk (dashed line) (imaged using MPLSM and SHG). Alignment of collagen fibers (white) is evident, and alpha-SMA+ cells can be seen on the PDMS surface (red). The vasculature (green, FITC-dextran) extends by forming perfused loops and sprouts. As the matrix remodels, the vessels also remodel as they advance. **a**: D1: day 1, D3: day 3, D6: day 6 post-TIC implantation. A pillar structure, which defines the height of the chamber, is indicated with * (from [493])

Intravital microscopy in window chambers is characterized by the following features: (1) It allows studies in an in vivo microenvironment, while ex vivo or in vitro studies require more extensive validation; (2) it enables visualization and measurement of the dynamics of the same vessels and cells as the new vasculature forms and changes in response to various treatments (Fig. 22); (3) it permits a variety of functional studies of angiogenic vessels which are being increasingly recognized in the angiogenesis field; (4) it allows dissection of the role of various mechanical (shear stresses, hoop stresses, etc.) and biochemical signals known to govern angiogenesis, (5) it allows investigation of the role of different organ microenvironments on vessel formation and function; and (6) a wide range or reagents are readily available for the murine system (Abs, genetic manipulation, etc.).

### 24.3 Limitations and challenges of intravital microscopy and window chambers

Intravital microscopy and window chambers experience some limits: (1) They are labor-intensive, making screening studies expensive; (2) creating the window may cause transient inflammation and inappropriate implantation of the window can induce tissue damage; the use of compromised windows affects angiogenesis in the windows (wound healing response); (3) for single-photon microscopes, depth penetration is limited to a few cell layers due to light scattering. Multiphoton microscopes permit deeper imaging but still limited to a few hundred micrometers. OCT, Doppler OCT, and SWIR permit deeper imaging, but are more expensive; (4) commercially available advanced imaging techniques are very expensive and the most advanced imaging techniques are not widely available.

### 24.4 Concluding remarks

Optical microscopy provides the high-resolution imaging needed to distinguish cell dynamics, ECM components, and intracellular features. By placing transparent windows over various organs in animal models, we can take advantage of optical microscopy to follow the formation of new vasculature noninvasively for time periods of weeks or months. This enables studies of neovascularization in normal processes such as wound healing and diseases such as cancer and allows detailed analyses of responses to drugs that target blood vessels.

## 25 The RIP1-Tag2 transgenic mouse model

The RIP1-Tag2 mouse model was one of the earliest oncogenic mouse models used in pioneering studies by Hanahan and Folkman to identify and characterize the angiogenic switch and multi-step progression to pancreatic neuroendocrine tumors. Due to its high vascularity, and synchronous and rapid tumor development, it became a valuable preclinical tool for developing and evaluating response and relapse from anti-angiogenic therapy from its earliest stages to the current day. Studies in the RIP1-Tag2 model predicted efficacy and helped to motivate clinical trials that led to the approval of two compounds, everolimus and sunitinib, for human pancreatic neuroendocrine tumors (PNET) in 2011.

### 25.1 The angiogenic switch

**Fig. 23:**
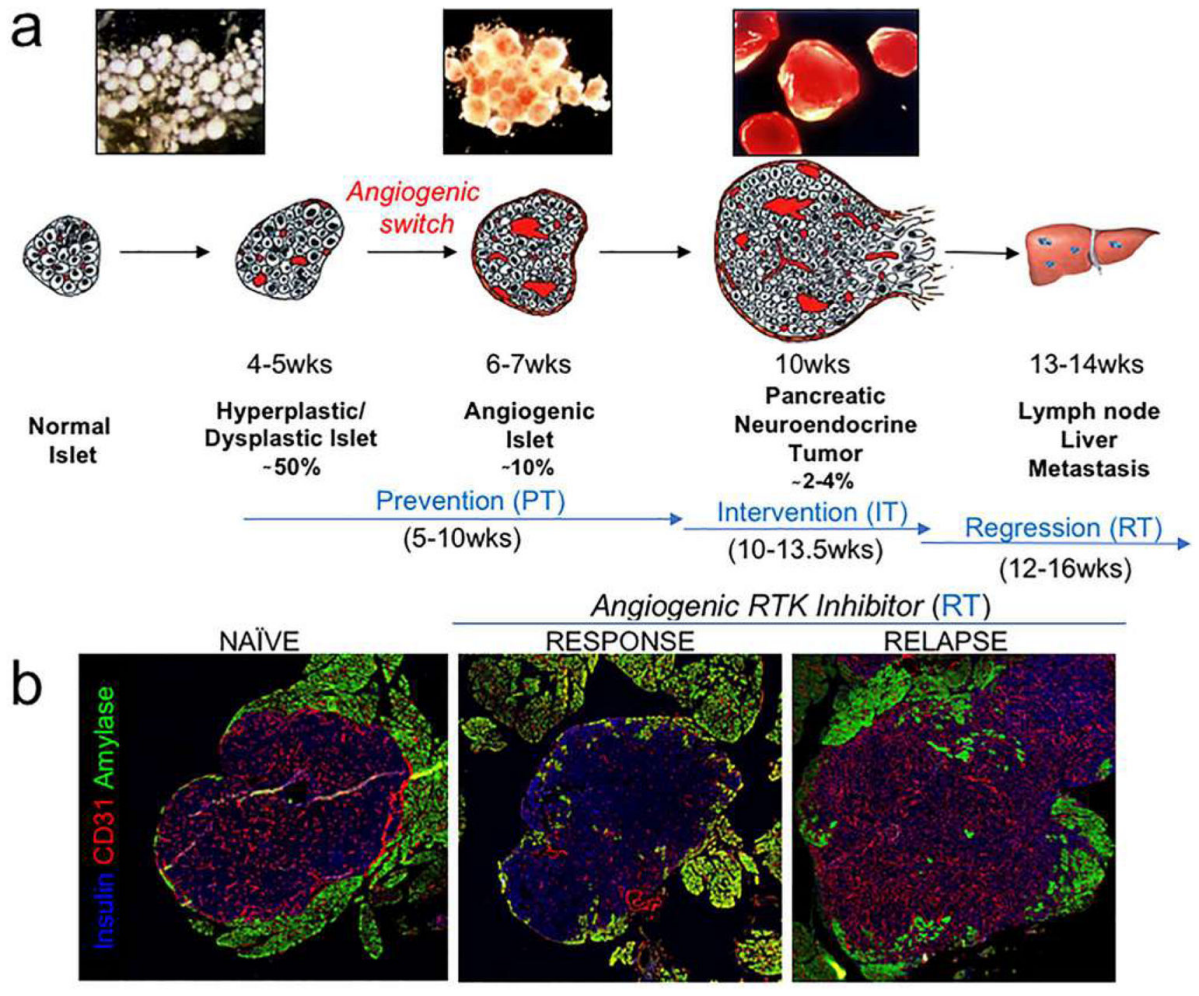
RIP1-Tag2 mouse model. **a** Multi-step progression to tumors in RIP-Tag2. Although oncogene expression begins during embryonic development (E8.5), the pancreatic islets initially have a normal anatomical and histological appearance (“normal” stage). Beginning at 4–5 weeks of age, hyperplastic and dysplastic islets begin to appear to comprise about 50% of islets by 10 weeks. Angiogenic islets appear beginning around 6 weeks of age, and represent 10% of all islets at 10.5 weeks. Angiogenic islets are recognized by their dilated blood vessels and microhemorrhages. Tumors form beginning at 9–10 weeks and represent 2–4% of all the islets by 14 weeks. About half of the tumors at end stage evidence either focal or widespread invasion to the surrounding acinar tissue. RIP-Tag2 mice die at approximately 14 weeks of age primarily due to hyperinsulinemia. **b** Anti-angiogenic Therapy Response and Relapse. Tumors treated with anti-angiogenic therapy using an RTK inhibitor starting at 12–15 weeks “Response,” or 12–20 weeks “Relapse,” when vessels have significantly rebounded. Tumors are stained with anti-insulin in blue, vessels are stained with anti-CD31 in red, and surrounding exocrine pancreas is stained with amylase in green

The RIP1-Tag2 mouse was developed by Douglas Hanahan as a result of his interest in using newly developed transgenic technology to use a well-characterized rat insulin promotor to express a viral oncogene, SV40 T antigen, in the beta cells of the pancreatic islets of Langerhans in order to study oncogenic transformation of normal tissues [520]. The first transgenic mice died at an early age from hypoglycemia, consequent to the rapid proliferation of beta-islet cells, which produced high insulin levels. Tumors from viable mice were characteristically bright red and highly vascularized, and it was immediately proposed that this was likely the result of what was later called the “angiogenic switch,” which Judah Folkman had postulated, must occur for tumors to grow beyond a small size [521]. But when and how is this switch activated? Co-culture of the total islet population with EC in a collagen matrix resulted in proliferation and migration of EC toward only a small subset of “angiogenic” islets to form capillary tubes, elegantly confirming that the angiogenic switch occurred in hyperplastic islets prior to tumor formation [522]. This also implies that a soluble factor [521], later identified as VEGF through multiple lines of evidence, was critical. In normal adult mammals, the vasculature is quiescent, and new blood vessels are formed through angiogenesis, wherein new capillaries sprout from existing vessels in a process largely driven through VEGF signaling. Hanahan and Folkman proposed that this angiogenic switch is a discrete early step in tumorigenesis and that the stages of progression that characterize RIP1-Tag2 tumorigenesis, where progressively smaller subsets of quiescent islets sequentially become hyperplastic, angiogenic, and finally progress to invasive carcinomas (Fig. 23a), also exist in human tumors [523]. In contrast to the popular hypothesis at that time that the angiogenic switch is induced by upregulation of pro-angiogenic factors, RT-PCR analysis of all VEGF ligands and their receptors, flt-1 and flt-2, as well as acidic fibroblast growth factor (FGF1) indicated that their expression levels were similar in islets before and after the angiogenic switch [524, 525]. The fact that normal non-transgenic islets already contained high levels of various pro-angiogenic factors suggested another mechanism in the activation of angiogenesis. Indeed, the switch from vascular quiescence to an angiogenic state involved the matrix met-alloproteinase MMP-9, which was secreted by infiltrating myeloid cells. MMP-9 rendered ECM-sequestered VEGF bioavailable to its receptor, thus triggering an angiogenic switch; this identification was confirmed using co-culture studies of angiogenic islets derived from MMP-9 KO RIP1-Tag2 mice and EC in collagen matrices—in contrast to wild-type islets, the MMP-9 KO islets did not induce EC capillary formation [526]. The result that VEGF-A is a pivotal factor in the angiogenic switch was further confirmed with an islet beta cell-specific knockout of VEGF-A in RIP1-Tag2 mice, which impaired both angiogenic switching and subsequent tumor growth [525].

### 25.2 Preclinical and clinical studies

These and many other concurrent studies identifying the VEGF/VEGFR2 axis as a powerful therapeutic target led to the development of numerous anti-angiogenic compounds. Sarah Parangi and colleagues were the first who used the RIP1-Tag2 model to perform preclinical studies with a combination of three early compounds with anti-angiogenic activity—AGM-1470, the antibiotic minocycline, and interferon alpha/beta [527]. The combination can reduce tumor growth, but not prevent it, and was an early preclinical success for anti-angiogenic therapy that helped to propel this class of inhibitors into the clinic [527]. The rapid and synchronous multi-step tumor progression that characterized the RIP1-Tag2 model made it then ideal for designing three different trial formats (Fig. 23a): early treatment at the hyperplastic stage to block the angiogenic switch commencing prior to tumor formation (prevention trial), treatment of mice bearing small tumors to determine whether tumor growth and progression can be stopped (intervention trial), and treatment of mice with substantial tumor burden and near death to test whether drugs can induce tumor regression and promote survival benefits (regression trial). These trials were first tested with AGM-1470, batimastat (BB-94), Fc-endostatin, Fc-angiostatin, or a combination of the latter two compounds and produced distinct efficacy profiles in the various trial formats [528]. These studies suggested that those anti-angiogenic compounds needed to be fine-tuned to target specific stages of disease progression and that combinatorial strategies can broaden the effects and enhance survival. Subsequent studies illustrating the importance of targeted therapies that disrupt both tumor EC and pericytes brought this notion to fruition in combination therapy trials with two different Sugen inhibitors, SU5416 and SU6668, that target VEGFR2 and PDGFR, respectively [529]. While the VEGFR inhibitor SU5416 was most potently effective against early-stage disease, SU6668 was shown to block further growth of end-stage tumors suggesting that PDGFR+pericytes in tumors present a complimentary target to EC for efficacious anti-angiogenic therapy. Combination therapies of these compounds were more efficacious against all stages of islet carcinogenesis than either single agent [529]. Congruently, subsequent studies with sunitinib (SU11248), which targets both VEGFR2 and PDGFR, showed remarkable efficacy in preclinical studies in RIP1-Tag2 mice and produced increased survival relative to monotherapy targeting VEGFR2 [530]. Collectively, these studies were used to incentivize the use of sunitinib in clinical trials for PNET. These clinical trials produced an impressive extension in progression-free survival that leads to the approval of sunitinib in 2011 [531], along with everolimus that primarily targets mTOR signaling. Interestingly, mTOR inhibitors also are very efficacious and produced increased survival in the RIP1-Tag2 model [532]. Thus, the RIP1-Tag2 model predicted the beneficial effects of both sunitinib and everolimus. However, significantly increased overall survival of RIP1-Tag2 mice, but not PNET patients treated with these inhibitors, can be reflective of the consequence of the different life spans of mouse versus human (approximately 15 weeks versus 70 years); therefore, a relatively short life extension can be significant. Also, preclinical studies in the RIP1-Tag2 mice are confounded by the fact that tumors are multifocal and that mice die from hypoglycemia, with relatively low (collective) tumor burdens and little metastasis relative to that characteristic of other mouse tumor models or human cancer. However, it is also noticed that evaluation of overall survival in clinical studies is confounded by the fact that placebo-treated PNET patients (unlike RIP1-Tag2 mice) elect to “cross-over” to the treatment cohort, likely reducing the calculated overall survival benefit in patients [533].

### 25.3 RIP1-Tag2 versus PNET tumors

Another major complication in interpreting preclinical studies using the RIP1-Tag2 mouse lies in fundamental biological differences from PNET. Tumors in the RIP1-Tag2 model are rapidly progressive and rarely form metastases, in contrast to the more indolent PNET tumors, although a subset of PNET tumors is more aggressive and metastasized [534]. Further, the tumor gene driving RIP1-Tag2 oncogenesis is a viral protein that interferes with p53 and retinoblastoma tumor suppressor function, while the genes associated with human PNET are predominantly chromatin-remodeling genes, DNA repair genes, mTOR-PI3K pathway genes, and menin mutations [534]. Notwithstanding these differences, expression profiling of human PNET tumors split them into three distinct groups, and remarkably two of them correspond to expression profiles identified in RIP1-Tag2 tumors [535]. Interestingly, human PNET tumors rarely adapt to culture, and while many cell lines from RIP1-Tag2 tumors (βTC cell lines) have been generated, they are surprisingly difficult to culture in comparison with other mouse tumor cell lines in spite of their aggressive growth in vivo.

### 25.4 Adaptation to anti-angiogenic therapy

The RIP1-Tag2 mouse represents a powerful model to assess therapeutic response and relapse to anti-angiogenics due to their very synchronous tumor progression, and this has made it a potent model to study adaptation to anti-angiogenic therapy. The response phase is characterized by tumor stasis and markedly reduced vascularity and vessel normalization blocking hemorrhage formation that produces whitish tumors, while the angiogenic relapse phase is characterized by tumor regrowth and revascularization (Fig. 23b). McDonald and colleagues investigated the kinetics and mechanisms underlying vessel regrowth upon withdrawal of anti-angiogenic therapy and found that although two different VEGFR inhibitors, AG-013736 or AG-028262, significantly regressed tumor vasculature, they left behind empty sleeves of pericyte-covered basement membrane which functioned as a scaffold for rapid revascularization after treatment withdrawal. They concluded that targeting pericytes and the sleeves of basement membrane appeared to be an important strategy to produce an enduring response to anti-VEGF therapy [536]. Casanovas et al. [537] performed preclinical studies in the RIP1-Tag2 model, which demonstrated that after a period of response to continuous anti-VEGFR2 monotherapy, tumors rebounded although blockade of VEGFR2 signaling persisted. They found that the mechanism for regrowth was induction of alternative pro-angiogenic factors, including the fibroblast growth factor family (FGF) and ephrins. In addition, the response phase is characterized by the angiostatic and immunostimulatory polarization of myeloid cells that contributes to pruning and normalization of the vasculature [538]. Tumors respond to these changes by activating and repolarizing myeloid cells to an angiogenic and immunosuppressive phenotype and by initiating an adaptive immune response by upregulating the negative immune checkpoint regulator, programmed cell death ligand PDL1; both immune adaptations limited the efficacy of VEGF/VEGFR inhibitors [538–540]. Another form of evasion from anti-angiogenic therapy that was characterized in the RIP1-Tag2 model (and glioblastoma) is that of increased invasion and metastasis following pharmacological and genetic targeting of the VEGF signaling axis [530], the consequence of increased hypoxia induced by vascular fallout. While increased invasion has not yet been found in PNET treated with anti-angiogenic therapy, it appears bevacizumab treatment can cause increased invasion in a subset of patients with glioblastoma multiforme [541], again highlighting the predictive power of this model.

### 25.5 Limitations and challenges

In spite of the fact that preclinical studies in the RIP1-Tag2 model predicted the efficacy of two approved therapies, the model is multifocal, rapidly progressive, and driven by a viral oncogene unlike its human counterpart. Because some PNET patients progress on available targeted therapies, it is desirable to further develop biomarkers of therapeutic response together with Patient Derived Xenograft (PDX) models from resistant tumors in humanized mouse models in order to identify beneficial secondary or concurrent therapies [533].

### 25.6 Concluding remarks

The RIP1-Tag2 model of beta islet-cell carcinogenesis has been instrumental in studying mechanistic underpinnings of tumor angiogenesis and in revealing adaptations to the environmental stresses elicited by anti-angiogenic therapies, leading to valuable insights and predictions regarding clinical successes and failure.

## 26 The MMTV-PyMT breast cancer model

The MMTV-PyMT mouse breast cancer model was described in 1992 by Guy, Cardiff, and Muller [542] and has been broadly employed for studying several aspects of breast cancer progression, such as the regulation of angiogenesis, cancer cell intravasation and dissemination, and metastatic colonization. In this transgenic model, the polyomavirus middle T antigen (PyMT) is expressed in the mammary epithelium from the long terminal repeat (LTR) promoter of the mouse mammary tumor virus (MMTV). The transforming activity of the PyMT is dependent on its association with several oncogenic proteins, resulting in the multifocal growth of mammary adenocarcinomas that metastasize to the lung [542].

### 26.1 Tumor angiogenesis in MMTV-PyMT mice

Lin et al. have examined breast cancer progression in MMTV-PyMT mice [543–545]. Early malignant transition occurs between 8 and 12 weeks of age and is associated with increased nuclear pleomorphism, peri-acinar infiltration by leukocytes, and angiogenesis [544]. The angiogenic switch—the conversion of a quiescent, non-angiogenic vasculature into one that is actively growing and infiltrative—generally demarcates the malignant progression of benign lesions [5]. Although the transition from hyperplasia to the adenoma/mammary intraepithelial neoplasia (MIN) involves the enlargement of the tumor mass in MMTV-PyMT mice, the density of functional blood vessels remains constant in these premalignant stages and is comparable to that of normal mammary glands [545].

A change in blood vessel distribution and density occurs during the transition from premalignant to malignant stages. In early adenocarcinomas, the acinar structures are replaced in the core of the lesion by small solid nodules displaying increased nuclear pleomorphism and cytologic atypia [545]. A high-density vascular network becomes evident in the solid nodular area, whereas surrounding premalignant acini are not associated with angiogenesis. Therefore, the development of adenocarcinomas, but not premalignant adenoma/MIN, is accompanied by angiogenesis in MMTV-PyMT mice. Of note, both high- and low-density vascular networks are found in early or late adenocarcinomas [545]. Variation in the features and density of blood vessels in transgenic (autochthonous) tumors is likely controlled by a multitude of parameters, including the spatiotemporally regulated expression of pro- and anti-angiogenic factors and inflammatory mediators, as well as biophysical forces, which dynamically control vessel functionality, growth, and regression in distinct tumor microenvironments [5].

### 26.2 Role of macrophages in MMTV-PyMT tumor angiogenesis

Monocytes and macrophages have been implicated in MMTV-PyMT tumor angiogenesis and progression [543, 545]. MMTV-PyMT mice carrying a homozygous mutation in the colony-stimulating factor-1 (*Csf1*) gene (*Csf1^op/op^*) recruit fewer tumor-associated macrophages (TAMs) than *Csf1*-proficient mice [543]. The macrophage-deficient phenotype of MMTV-PyMT/*Csf1^op/op^* mice is associated with delayed tumor progression (decreased incidence of invasive adenocarcinomas and pulmonary metastasis) and defective tumor angiogenesis [543, 545]. Conversely, the genetic overexpression of *Csf1* in the mammary epithelium of MMTV-PyMT mice results in the premature accumulation of macrophages around hyperplastic lesions and adenomas and accelerates both the development of an angiogenic vasculature and tumor progression [545]. These findings are consistent with results obtained using a macrophage-depleting CSF1 receptor (CSF1R) inhibitor [546] and agree with studies in other mouse tumor models, such as RIP1-Tag2 transgenic mice [547].

Conditional overexpression of vascular endothelial growth factor-A (*Vegfa*) in the mammary epithelium of 4–6-week-old MMTV-PyMT mice was sufficient to induce the formation of enlarged, angiogenic blood vessels around premalignant acini [548]. Also, VEGF-A overexpression rescued macrophage infiltration, stimulated the formation of a high-density vascular network, and restored tumor progression in MMTV-PyMT/*Csf1^op/op^* mice [548]. These findings suggested that macrophages can promote tumor angiogenesis in MMTV-PyMT mice, at least partly, through VEGF-A [548]. In another study, conditional deletion of *Vegfa* specifically in myeloid-lineage cells, which encompass macrophages, altered vascular patterning in MMTV-PyMT mice [549]. This phenotypic conversion involved decreased vessel tortuosity and density and increased pericyte coverage, which are consistent with an attenuated angiogenic response. While overall VEGF-A levels were unabated by loss of myeloid-derived VEGF-A (likely due to substantial VEGF-A production by cancer cells), the phosphorylation of endothelial VEGF receptor 2 (VEGFR2) was decreased in the tumors [549]. These findings suggest that myeloid cells, or subsets thereof, provide a non-redundant source of bioactive and pro-angiogenic VEGF-A in the perivascular microenvironment of MMTV-PyMT tumors. Macrophages enwrap angiogenic blood vessels in MMTV-PyMT tumors [550, 551], which may explain the importance of macrophage-derived VEGF-A for tumor angiogenesis [549]. However, it should be noted that attenuation of angiogenesis does not necessarily translate into tumor inhibition. Indeed, Stockmann et al. observed accelerated tumor progression in MMTV-PyMT mice with myeloid-specific *Vegfa* deletion [549]. This finding can be attributed to the “normalized” structure of the blood vessels, which may have led to improved tumor oxygenation and cancer cell growth [552].

Besides VEGF-A, TAMs secrete additional pro-angiogenic factors [5]. For example, the genetic deletion of *Wnt7b* in TAMs reduced the expression of WNT/β-catenin mitogenic target genes in vascular endothelial cells and decreased the MVD in MMTV-PyMT adenocarcinomas [553].

MMTV-PyMT mice have also been instrumental for studying macrophage–vascular interactions in the mammary tumors. The genetic inactivation of *Tie2*/*Tek* in TAMs did not block their recruitment to tumors, but impaired their ability to associate with immature blood vessels and sustain tumor angiogenesis [551]. Further to supporting angiogenesis, perivascular TAMs enhance vascular permeability through VEGF-A, which facilitates cancer cell intravasation and metastasis in MMTV-PyMT mice [554]. The association of macrophages with tumor blood vessels is increasingly recognized as an important determinant for early breast cancer metastasis in both MMTV-PyMT mice and patients with breast cancer [554–556].

### 26.3 Anti-angiogenic therapy in MMTV-PyMT mice

The pharmacological inhibition of VEGF-A signaling attenuates tumor angiogenesis in both transgenic MMTV-PyMT mice and mice carrying orthotopic MMTV-PyMT tumor transplants [557, 558]. More marked anti-angiogenic effects are observed when VEGF-A and angiopoietin-2 (ANGPT2) are neutralized concomitantly in the mammary tumors [557, 558]. Dual VEGF-A and ANGPT2 blockade also leads to substantial tumor inhibition and significantly extends the survival of transgenic MMTV-PyMT mice [558]. Further to suppressing tumor angiogenesis and abating the density of the vascular network, combined VEGF-A and ANGPT2 blockade normalized the structure of the blood vessels that survived in viable tumor regions and facilitated the extravasation and perivascular accumulation of activated, interferon-γ (IFNγ)-expressing CD8^+^ T cells [558]. These tumor phenotypes were likely due to the complementary actions of ANGPT2 and VEGF-A inhibitors on the tumor microenvironment: Whereas VEGF-A blockade has more marked vascular-pruning activity, at least acutely [559], ANGPT2 blockade limits angiogenesis [551] and enforces the maturation and stabilization of the remaining vessels, making them permissive to T cell extravasation and trafficking [560, 561]. Thus, MMTV-PyMT mice provide a useful model for studying the kinetics of angiogenesis inhibition and vascular normalization in response to anti-angiogenic drugs.

### 26.4 Limitations and challenges

Although angiogenesis has been extensively studied in primary adenocarcinomas, little is known of the mechanisms controlling vascularization of pulmonary metastases in MMTV-PyMT mice. Because macroscopic pulmonary metastases become evident only in aging mice (12–16 weeks of age), the development of multifocal primary tumors may impose significant constraints on the investigator’s ability to examine metastatic lesions whose mass would be large enough to stimulate angiogenesis. In this regard, it is increasingly appreciated that metastatic tumors may rely, at least partly, on vessel co-option and be less dependent on angiogenesis than primary tumors [562]. Further studies are required to establish whether pulmonary metastases that develop in MMTV-PyMT mice vascularize through angiogenesis, vessel co-option, or both.

## 27 Tumor implantation models

### 27.1 Implantation of angiogenic tumors

Tumors may be implanted in mice as xenografts or in a syngeneic context. Implantation may also be done subcutaneously or orthotopically at the correct organ site where the tumor usually grows. There are many angiogenic tumor models to cite only a few such as glioma (U87 human glioblastoma, mouse GL261 glioma), breast (MDA-MB-231, 66cL4 mouse mammary tumor), lung (Lewis lung carcinoma, human LNM35 lung) melanoma (B16 mouse melanoma), prostate (human prostate CWR22Rv1 tumor) [563]. Tumor cells may be injected as single-cell suspension, embedded in matrix such as matrigel or implanted as spheroids or organoids. Cells may be transduced with a suitable reporter construct (such as luciferase), and tumor development may be followed by imaging. Usually at a given time point, animals are killed and tumors are processed for immunohistochemistry, RNA expression analysis, or protein detection (immunoblot). The angiogenic response is analyzed by immunohistochemistry or immunofluorescence using appropriate antibodies for endothelial cells (CD31, CD34) or pericytes (alpha2SM actin, desmin). This analysis will give information about vessel density, vessel size distribution, and vessel quality (tight or loose pericyte coverage). The angiogenic response is usually high in the viable core of the tumor. At the invading front, tumor cells may use preexisting vessels for invasion.

It is of note that tumors may exhibit different angiogenic responses when they are either implanted in orthotopically or subcutaneously as it has been shown in the case of astrocytomas [564].

### 27.2 Detection of “non-angiogenic” tumors, which rely instead on “vessel co-option”

While there is an overwhelming body of preclinical/experimental and clinical evidence accumulated over 40 years showing the importance of sprouting neo-angiogenesis in tumor growth, progression, and metastasis, it is increasingly appreciated that there are many circumstances in which tumors can grow and expand without inducing any new blood vessel capillaries. This realization has major implications for understanding some of the clinical efficacy limitations including failures of anti-angiogenic drugs. Thus, new blood vessels can be formed by a process of the “splitting” of existing vessels, a process known as intussusception, as discussed above. Or, certain tumors, especially neural crest-derived cancers, may form blood forming channels by “vasculogenic mimicry” whereby tumor cells line the vessel lumen, rather than authentic host EC. However, the most dominant manifestation of non-angiogenic tumors is likely “vessel co-option” and it is particularly prominent in vascular-rich organs that are the most common sites of metastatic disease, namely lung, liver, lymph nodes, and brain [117].

Broadly speaking, vessel co-option refers to the ability of tumors to parasitize preexisting blood vessels of the tissue, bypassing the requirement of developing new ones. The modern era of vessel co-option may have begun in 1996–1997 with the publication of two pathology reports by Pezzella and colleagues. Therein, a pattern of tumor growth of non-small cell lung cancer and lung metastases was recognized which made use of alveolar air spaces for expansion and alveolar vessels for blood supply without any indication of angiogenesis [121, 565]. Histopathological descriptions of similar growth pattern in the lungs and analogous patterns in the brain and liver date to the late 1800s and early 1900s. The distinction between so-called newly formed versus pre-existing blood vessels was noted in the past, but the biological significance of each form was generally not appreciated. This has changed in the era of anti-angiogenic therapy—for example, targeting the vascular endothelial growth factor (VEGF) pathway to impair and cause regression of new vessel sprouts does not deplete preexisting vessels. Tumor exploitation of vessel co-option has recently been shown to account for instances of intrinsic and acquired resistance to anti-angiogenic drugs, both preclinically [122, 566, 567] and clinically [119]. Moreover, growth patterns associated with vessel co-option are emerging as important for patient prognosis [120, 568].

There is currently no “assay” as such to quantitate the extent of vessel co-option in tumors. Conventional measurement of tumor microvessel densities (MVD) within tumors has high potential for error when one considers that normal tissue vessel densities—as well as tumors where cancer cells surround these vessels—may be high [569]. Rather, histopathological analysis is currently used with certain key features being looked for. Some examples are briefly described below; more detailed descriptions can be found elsewhere [117, 569].

### 27.3 Some “hallmarks” of detecting vessel co-option in tumors growing within the lungs, liver, and brain

Though the histological structures of these organs are distinct, tumors within these sites that co-opt preexisting blood vessels—either as part of its overall mass or throughout—often have some of the following features:

1. *The architecture of the normal host tissue is preserved within the tumor.* This is in contrast to angiogenic tumors, which tend to destroy the surrounding tissue. Tissue architecture preservation has been aided by immunohistochemistry for basement membrane and epithelial markers showing continuity of form across the tissue–tumor interface.
2. *Lack of surrounding capsule or desmoplastic tumor boundary.* Angiogenic tumors often form defined masses or nodules, a symptom of the inflammatory response of the host to the tumor, but this is lacking in “purely” vessel co-opting tumors.
3. *Invasive, infiltrative, and replacing tumor growth.* Minimal compressive tumor growth and compression of normal tissue structures. Rather, tumor cells tend to infiltrate along preexisting vessels, often in direct contact with epithelial–endothelial basement membranes. Epithelial cells may become destroyed or stripped from their vessels during this process. Tumor borders are irregular.
4. *Incorporation of characteristic cells and structures of the tissue into the tumor.* Parenchymal epithelial cells (e.g., lung pneumocytes, liver hepatocytes, neuralglia) and larger intact structures (e.g., lung bronchioles and large vessels in the lungs, portal triads in the liver) are taken up into the expanding infiltrative tumor mass and get locked into the tumor.
5. *Maintained vessel morphology and marker expression.* Co-opted vessels are known to be remodeled by surrounding tumor tissue and therefore may not in all cases appear “normal.” Newly recruited co-opted vessels in particular may maintain morphological and molecular characteristics of tissue endothelium (e.g., GLUT-1 and p-glycoprotein endothelial expression in brain tumors).
6. *Low rates of endothelial cell proliferation relative to angiogenic tumors.* Specific molecular markers of coopted blood vessels are not currently known; thus, several criteria are used to distinguish between vessel subtypes and differential growth patterns of tumors. Vessel co-option was first identified in tumors from patients; however, experimental mouse models have also been shown to recapitulate the growth patterns observed in human tumors.

The major question now for future studies is whether “anti-vascular” therapeutic strategies can be developed that target co-opted vessels, in addition or instead of angiogenic vessels, without impacting the normal vasculature; some possibilities have recently emerged, for example using antibody–drug conjugates [570].

**Fig. 24:**
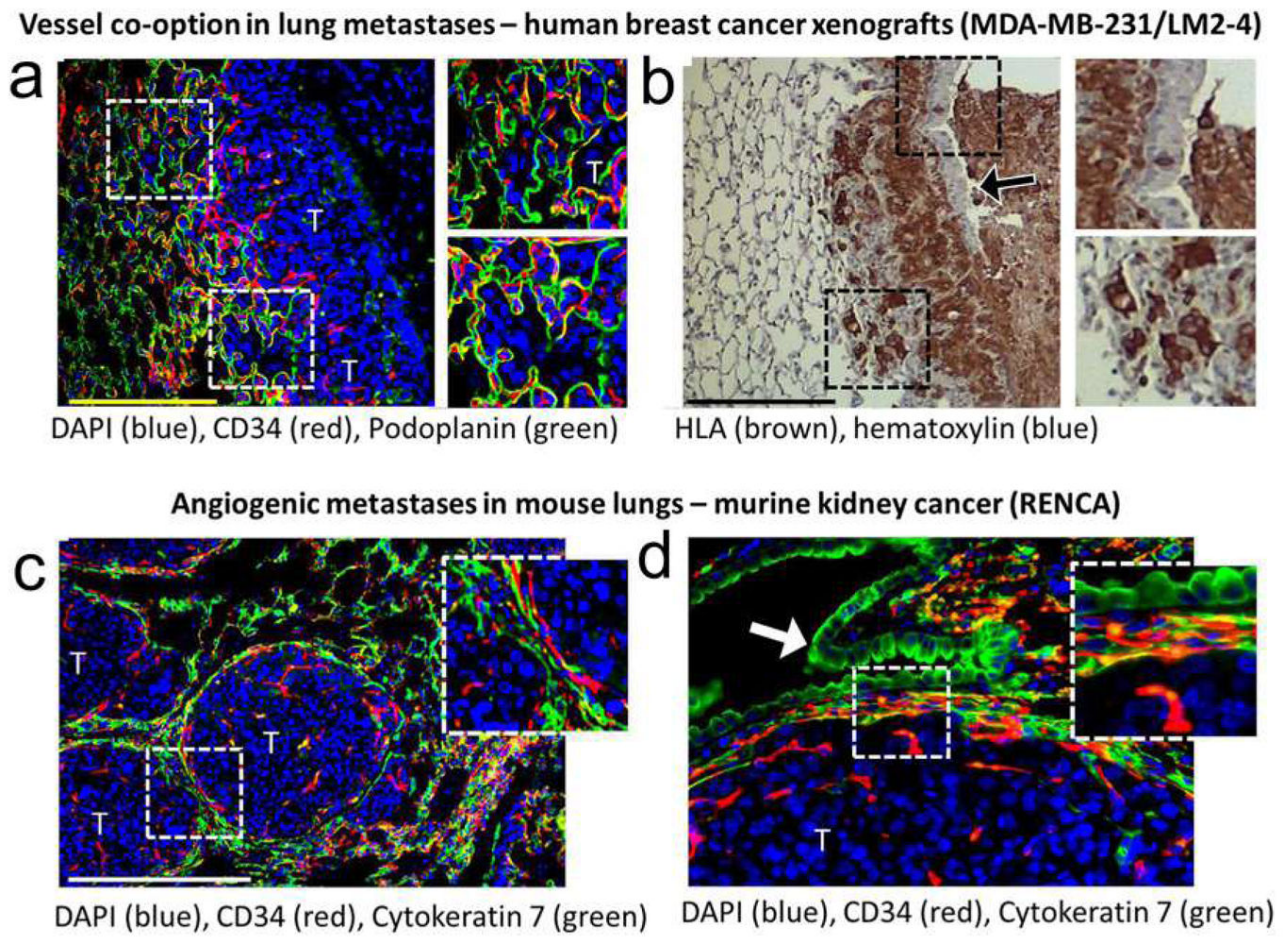
Visualization of vasculature in transplanted mouse models. Immunofluorescence and immunohistochemical staining of forma-lin-fixed mouse lung samples to enable differentiation between vessel co-option and angiogenesis in tumors. **a**, **b** Vessel co-opting tumors are observed in spontaneously formed lung metastases from mice with orthotopically implanted then surgically resected MDA-MB-231/LM2–4 breast tumors. In **a** sections are stained for alveolar cell marker podoplanin, EC marker CD34 and nuclei marker DAPI. Tumor cells can be seen filling alveolar spaces along the border and incorporating alveolar capillaries into the tumor core. In **b** is the corresponding section stained for HLA human cell marker and hematoxylin to show the presence of tumor cells with respect to host stroma and lung parenchyma. A bronchiole is also seen to be taken into the tumor and gradually filled with tumor cells. The tumor border is irregular. **c**, **d** Angiogenic growth is observed in spontaneously formed lung metastases from mice bearing intra-renal implanted RENCA tumor cells that later underwent nephrectomy. Sections are stained for alveolar and bronchial epithelium cell marker cytokeratin 7, EC marker CD34 and nuclei marker DAPI. RENCA tumors grow in “cannonball” shape, compressing lung tissue and excluding them. The lung–tumor interface of another nodule is shown at high magnification in **d**. Lung tissue is compressed or “pushed” aside to allow tumor expansion. The tumor border is smooth, and microvessels are not associated with alveolar epithelium within the tumor. Scale bar represents 200 μm. Regions in dashed boxes are expanded on the right. “T” = tumor. Arrow = columnar bronchial epithelium

Shown in Fig. 24 are preclinical examples of both vessel co-opting and angiogenic tumors. Lung metastases from mice with breast cancer cell (MDA-MB-231/LM2–4) vs. kidney cancer (RENCA). The former develops purely non-angiogenic vessel co-opting lung metastases, whereas the latter forms both angiogenic (shown here) and vessel coopting lung metastases. Staining for lung-specific epithelial and EC markers shows the architecture of blood vessels in lung tissue and metastases. In vessel co-opting lung metastases, alveolar cells surrounding capillaries are taken up by the expanding mass at the tumor border, but alveolar epithelial cells are gradually shed from vessels toward the tumor center while capillaries remain.

### 27.4 Limitations and challenges

Implantation models are generally very useful in deciphering mechanisms of the tumor angiogenesis and the role of given angiogenesis stimulator or inhibitor, receptors or modulating intracellular factors. The only drawback is that the adaptive immune system in xenotransplantations is absent and thus the data must be interpreted with care. The other challenge is the implantation site. Usually, it is better to orthotopically implant tumor cells since the correct host tissue is present in this case. Another challenge is whether or not to implant cell suspensions or spheroids. Spheroids seem in many cases better suited because they already adopt a 3D tissue configuration which preserves some of their genetic program that is encountered in a 3D tissue.

Co-optive growth may be induced by anti-angiogenic therapy as it is encountered in glioblastoma for instance. Thus, a switch from angiogenesis to co-option may occur in this case. It would be important to develop therapies against co-opted vessels but this may be damaging because there are for the moment no specific markers discovered for coopted vessels.

### 27.5 Concluding remarks

Tumor implant models are of great value to decipher tumor vessel interactions. Despite several limitations, this is still important methodology that is useful in characterizing many molecular mechanisms of tumor angiogenesis.

## 28 Mouse hind limb ischemia model

Peripheral arterial disease (PAD) is caused by atherosclerosis and occlusion of peripheral blood vessels and results in intermittent claudication or in more severe stage in critical limb ischemia. The prevalence of PAD increases with age, affecting 6% of individuals aged 50–60 years, and 10–20% of individuals aged > 70 years [571, 572]. In the upcoming years, the number of PAD patients will increase even more due to the aging of the population and the increase in number of patients with obesity and type 2 diabetes. Unfortunately, current therapies for improving the perfusion of the lower limbs in patients with PAD are hardly effective [573, 574]. Therefore, much effort has been put in the last decade in defining new strategies to improve the blood flow to the lower extremities and to promote blood vessel growth. These strategies that are based on the concept of therapeutic neovascularization are often also called therapeutic angiogenesis or more general, neovascularization [573].

Neovascularization comes in three anatomical and physiological forms; angiogenesis, arteriogenesis, and vasculogenesis [574]. Angiogenesis describes an expansion of the microvasculature, because of sprouting of EC from preexisting capillaries, followed by their proliferation, migration, and capillary formation and is mainly hypoxia driven [246]. By contrast, arteriogenesis describes the remodeling of existing arterioles into collateral arteries so that they can deliver more blood flow to the limb [575]. Finally, adult vasculogenesis describes the incorporation of circulating (progenitor) cells into the regenerating microvasculature [573]. In PAD, neovascularization primarily occurs in the form of arteriogenesis or collateral formation, although angiogenesis in more distally located ischemic areas does occur.

To test new therapeutic approaches to induce neovascularization, as well as unraveling the complex cellular and molecular mechanisms involved in the regulation of the neovascularization process, mouse models mimicking critical limb ischemia have been developed. Although these models may vary in crucial aspects, commonly they are referred to as the mouse hind limb ischemia model. Being in vivo models they are complex and not always straightforward to interpret, but they faithfully reproduce the physiology of blood recovery in PAD after stenosis of the original conduit. In this section, we will discuss the critical aspects of the variations in the use of the mouse hind limb ischemia model, their consequences for interpretation of the data obtained, and the limitations and pitfalls in their use.

### 28.1 Mouse model for hind limb ischemia

**Fig. 25:**
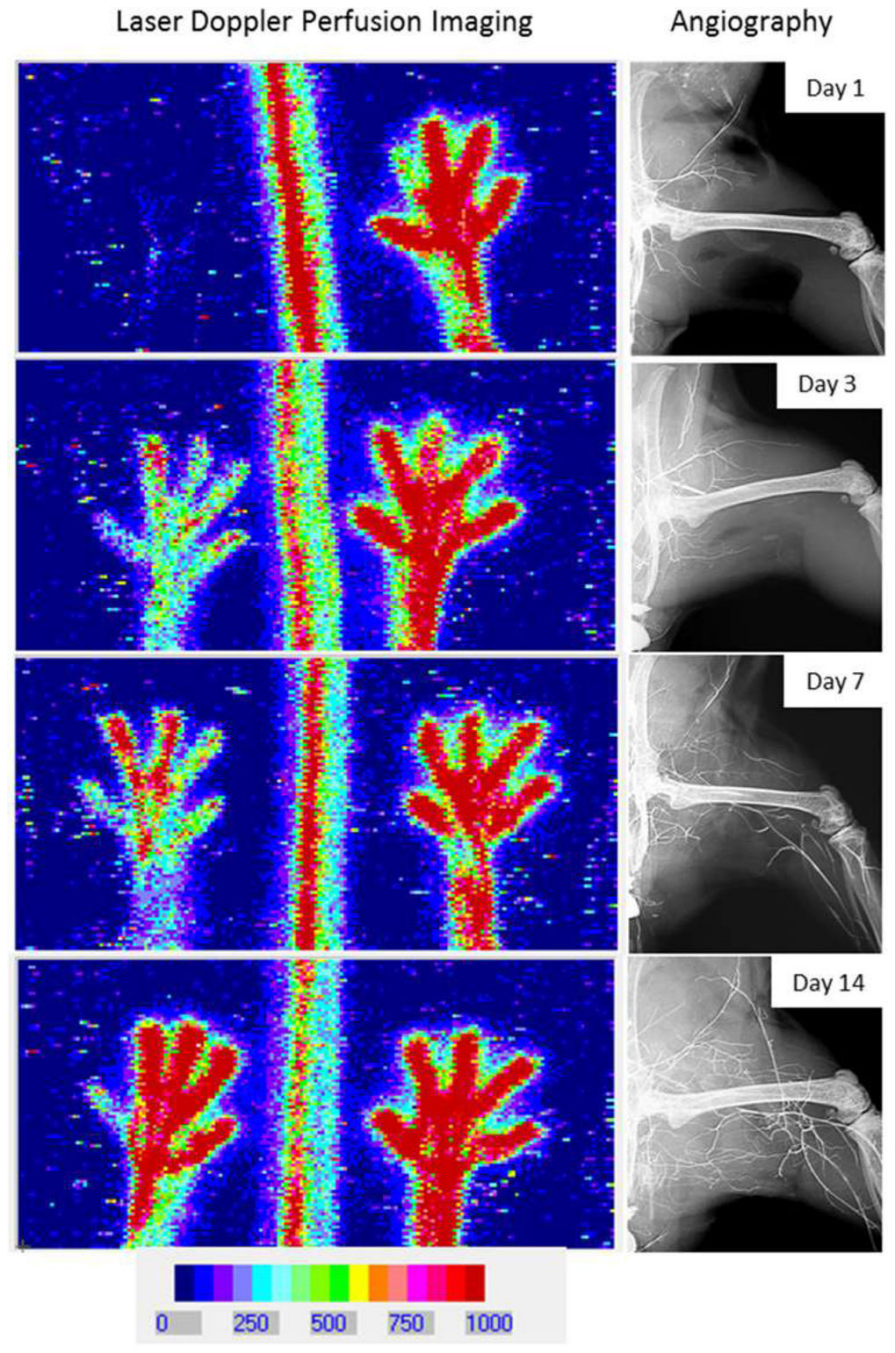
Laser Doppler perfusion imaging and angiography of blood flow. Analysis of blood flow recovery in time by laser Doppler perfusion imaging (left panels) and angiography (right panels)

Couffinal et al. were among the first to describe a mouse model for hind limb ischemia [576]. They induced acute hind limb ischemia by ligating the proximal end of the femoral artery, and the distal portion of the saphenous artery, followed by excision of ligated part of the femoral artery and attached side branches. The recovery of blood flow in the ischemic limb due to angiogenesis and arteriogenesis was monitored by laser Doppler perfusion imaging (LDPI) [576], Fig. 25. Subsequently, many groups used similar approaches and variants of this model [577, 578]. Surgical procedures range from a single ligation of the femoral or iliac artery [579–581] to a complete excision of the artery [576] and sometimes even the vein and the nerve were dissected as well [582, 583]. Several excellent review papers have been written on these variations in the hind limb ischemia model [584–587]. When choosing a particular variant of the mouse hind limb ischemia model for a study, it is important to define the goal of the study. For instance, when testing new pro-arteriogenic approaches, there should be an appropriate therapeutic window in which an improvement of blood flow recovery can be monitored. In mice that rapidly form new collaterals, for example, C57BL/6 mice with a single ligation of the femoral artery, it is difficult to monitor an increase toward an even faster collateral formation [588], and therefore a model with more severe injury is required [589, 590]. On the other hand, such a fast model is ideal for mechanistic studies in which the effects of the deficiency or inhibition of crucial factors on blood flow recovery are studied [591, 592]. The fast recovery model is also associated with less animal discomfort.

Another crucial question is which aspect of neovascularization will be studied. Arteriogenesis is the remodeling of preexisting arterioles to larger diameter collateral blood vessels required for restoring the blood flow to the distal ischemic parts of the limb, whereas angiogenesis is the sprouting of new (capillary) vessels into the ischemic tissue. One can imagine that the excision model to induce hind limb ischemia results in the disruption of the preexisting arterioles in the adductor muscle, and thus the presumed substrate for collateral formation. In pro-angiogenic intervention, however, severe ischemia in the calf muscles in the excision model is warranted.

A good comparison of the various models has also been hampered by the lack of a proper description of the anatomy of the blood vessels in the mouse limb. Hopefully the recent excellent description of the arterial anatomy of the murine hind limb by Kochi et al. [593] will contribute to a better understanding of the outcomes of the different ischemia models.

### 28.2 Variants of the surgical procedure to induce mouse hind limb ischemia

#### General aspects of the surgical procedures

Before surgery, mice are anaesthetized with an intraperitoneal injection of a combination of midazolam, medetomidine, and fentanyl. In all models, the femoral vein and nerve are preserved. After surgery, the skin is closed with 6/0 Ethilon sutures (procedures adapted from [585]).

#### Single electrocoagulation of femoral artery

Directly after incision of the skin in the left inguinal region, the subcutaneous fat pad in the thigh becomes visible and is pulled aside distally. After dissection of the artery from the nerve and vein, ischemia is induced by electrocoagulation of the left femoral artery, proximal to the superficial epigastric artery. Electrocoagulation results in complete occlusion of the artery. After electrocoagulation, the proximal end of the artery moves proximally into the surrounding tissue and the distal end moves distally, so there will be a distance of a few millimeters between both ends after the surgical procedure.

#### Single electrocoagulation of iliac artery

A larger skin incision in the inguinal region is made for this procedure. Furthermore, there is no need to cleave the fat pad. For exposure of the iliac artery, a retroperitoneal approach is used. By carefully moving the peritoneum proximally with a cotton swab, a good exposure of the iliac artery is possible. In addition, preparation of the artery from the vein is necessary. The internal iliac artery serves as a landmark; direct proximally of the internal iliac artery an electrocoagulation of the common iliac artery is performed.

#### Double electrocoagulation of both femoral artery and iliac artery

For a double coagulation model, both common iliac artery and femoral artery are electrocoagulated. First, an electrocoagulation of the common iliac artery is performed followed by electrocoagulation of the femoral artery. These coagulations are at the same anatomical levels as in the single electrocoagulation procedures of the femoral artery and the iliac artery.

#### Total excision of femoral artery

The upper thigh is exposed through an inguinal to knee incision. A proximal part of the femoral artery is removed after double ligation. Then, all side branches are isolated and coagulated. The distal ligation site is at the popliteal artery level distal from the saphenous artery bifurcation. The whole artery in between is removed.

### 28.3 Analysis of blood flow recovery and neovascularization

Traditionally, blood flow recovery is analyzed by Laser Doppler Perfusion Imaging (LDPI) where the flow in the footpads of the ligated and contralateral limb is measured and expressed as the ratio between the ligated and unligated footpad. The restoration of the blood flow in time is used as a readout for neovascularization (angiogenesis and arteriogenesis combined) in the ischemic limb.

Next to the LDPI-based flow analysis, immunohistochemical analyses of the neovascularization are used. Usually, the angiogenic response is studied in the distal part of the limb, the ischemic calf muscle (gastrocnemius), using EC staining (CD31, von Willebrand is in absolute numbers per area or as number of blood vessels related to number of myocytes). In addition, the arteriogenic response, (i.e., newly formed collateral arteries) is commonly studied in the adductor muscle. They are usually stained with alpha smooth muscle actin to demonstrate the arterial nature, whereas size and location are indicative of their collateral nature.

In addition to these two commonly used methods to monitor the neovascularization in the ischemic limb of the mouse, sometime a functional test is used in which the use of the foot and limb is analyzed for instance by assessment of the plantar/dorsiflexion [594]. In the more severe models, necrosis of toes or even feet ensues, leading to auto-amputation. Semiquantitative scores have been developed and used to assess efficacy of interventions. Moreover, the increased sensitivity of current imaging methods may represent a major step forward. For example, the use of micro-CT analysis (which previously was typically used as a postmortem procedure due to the low sensitivity and lack of good contrast agents [585], has now become a method for in vivo imaging of the microvasculature [595], enabling the analysis of the neovascularization over time. Recently, it was demonstrated by Hendrikx et al. [596] that single photon emission computed tomography perfusion can be used to analyze neovascularization processes in the mouse hind limb, even with such a resolution that they can demonstrate that LDPI analysis underestimates the revascularization processes in this model.

### 28.4 Limitations and challenges

One of the major limitations of the currently used hind limb ischemia model is the acute nature of the ischemia, whereas in the patients with peripheral artery disease the ischemia develops gradually. Although an interesting approach for gradual induction of ischemia in mice using ameroid constrictors has been described by Yang et al. [597], this model has not received large follow-up.

The capacity for vascular regeneration and neovascularization differs strongly between commonly used mouse strains [598, 599]. For many years, it has been known that C57BL/6 mice have a stronger neovascularization capacity than Balb/c mice [581, 600] and only recently, it has been discovered that this difference in regenerative response is linked not only to a difference in the preexisting collateral bed in the various strains [598] but that it also can be attributed to a specific gene locus in chromosome 7 of the mouse [601–604]. Because of their slow regenerative response, Balb/c mice are frequently used for hind limb ischemia studies, on the assumption that this slow response better mimics the situation in patients with PAD that display a poor regenerative capacity. A recent study by Nossent et al. [605] compared the mRNA expression pattern of angiogenesis and arteriogenesis related genes in the hind limb of C57BL/6 and Balb/c mice. This showed that in Balb/c mice a stronger upregulation of pro-angiogenic and pro-arteriogenic genes can be observed when compared to C57BL/6 mice, despite the poorer regenerative phenotype in Balb/c mice. These findings suggest that rather than a more accurate model of human critical limb ischemia, Balb/c mice lack a thus far unknown factor that is crucial for vascular regenerative response.

Since inflammation and the immune system play an important role in the regulation of the angiogenic and arteriogenic responses in the hind limb ischemia model and several immune cells including monocytes, T cells, and NK cells are crucial in neovascularization [579, 581, 591, 592, 606–609], the use of immune-compromised mouse strains for cell therapy approaches with human cells is not ideal and the results of these studies should be interpreted with caution. This becomes even clearer when the fate of injected cells, and their rapid disappearance even from syngeneic mouse is taken into account [609]. A very comprehensive review on the use of various immunocompromised mouse strains in hind limb ischemia models for human cell therapy validations has recently been published by Thomas et al. [594] and provides valuable information on the choice of the proper model.

Last but not least, a major limitation for most of the hind limb ischemia mouse studies is that they are performed in healthy young mice, whereas the PAD patients usually are older, and have atherosclerosis, type-2 diabetes, or other comorbidities. These comorbidities strongly affect the neo-vascularization responses in mice [610, 611]. Moreover, induction of vascular remodeling may also affect the underlying atherosclerotic disease leading to arterial occlusion. This phenomenon is described as the Janus phenomenon of neovascularization by Epstein et al. [612].

### 28.5 Concluding remarks

Many variants of the mouse hind limb ischemia model are used; researchers should be aware of the different options, select the model that fits best the goal of their experiments, and properly describe the type of model used. It should also be noted that although this model is commonly referred to as an angiogenesis model, the mode of neovascularization is a true combination of collateral formation and angiogenesis.

## 29 Large animal models for myocardial angiogenesis

There is little doubt that large animal models are needed for translational and preclinical studies of myocardial angiogenesis. Advantages of large animal models over smaller animals are that the heart better resembles human heart in size, anatomy, and function. Also, the same imaging equipment and treatment strategies, including similar dosage, can be used. Due to ethical reasons and a good resemblance to human, sheep and pigs are nowadays most widely used large animals in myocardial angiogenesis studies. Recent developments in these models are briefly described in this chapter.

### 29.1 Acute ischemia models

Acute myocardial ischemia models have been widely used, as they are rather easy to replicate in large number of animals. It will also be easy to make interventions if any arrhythmias occur. The models used are either surgical or catheter-based, very consistent, and variation between the animals is usually low. In surgical models, manipulation of the coronary arteries from a thoracotomy opening to induce ischemia is a widely used method [613]. However, from the thoracotomy opening, surgery affects the potential therapeutic approaches as pericardial attachments complicate further interventions and make it difficult to evaluate possible effusate accumulation, which often complicates pro-angiogenic therapies.

On the contrary to surgical models, catheter-based endovascular models leave much of the heart intact and post-operational complications are much less frequent. Widely used catheter-based ischemia models include ischemia–reperfusion model [614], infarction due to an intracoronary injected solution [615] and intracoronary coil occlusion [616].

### 29.2 Chronic ischemia models

A healthy animal has several mechanisms to respond to acute ischemia, which chronically ischemic patients may not have anymore. This has led to the development of more slowly developing occlusion and ischemia models to counteract and expand these adaptation mechanisms and to better mimic the lack of necrosis, acute inflammation and strong endogenous angiogenic stimuli, which takes place in acute ischemia models [617].

Very widely used gradual obstruction model is a surgical ameroid constrictor model [618]. However, as a surgical model, this has the same disadvantages as described above.

Endovascular models have also been recently developed. Catheter-based models include the use of copper stents [619], copper-plated stents [620], or oversized steel stents [621], where in-stent restenosis causes stenosis in the coronary artery. However, in these stent models, the stenosis size is variable, and its development cannot be controlled. There is also a ligated hourglass-shaped stent-graft model [622], but in this model, the exact time of total occlusion is not known. A recent bottleneck stent model can be varied for either reversible myocardial ischemia or ischemic cardiomyopathy [623], depending on if the stent is allowed to occlude in this model. Occlusion is controlled by gradual reduction of antiplatelet and antithrombotic therapy.

### 29.3 Non-ischemic heart failure models

In addition to ischemic heart failure models, non-ischemic models of heart failure have been developed that might be used to study neovascularization as a therapeutic approach for the improvement of cardiac function. In a recent model, heart failure is induced by chronic pulmonary hypertension [624]. To induce left ventricular hypertrophy and heart failure, different variations of aortic banding have been used [625]. Pacing models to induce heart failure have also been extensively used [626].

### 29.4 Myocardial stainings

Immunohistochemical stainings are used to histologically detect the effects of angiogenesis. Hematoxylin–eosin staining is used for tissue morphology characterization. Endothelial cells can be stained with PECAM-1, which is also known as CD31. Lymphatic endothelium can be stained with LYVE-1 or PROX-1. These pericytes, the smooth muscle cells surrounding blood vasculature, are stainable with α-SMA.

### 29.5 Challenges and limitations

Large animal models are the last translational step from bench to the first clinical trials. Therefore, using these models requires careful planning. Efficient delivery of the therapeutic agent, be it cellular, viral, or growth factor-based therapy, is a very critical factor for successful treatments. Earlier, mainly intramyocardial injections were used as these can provide robust and efficacious transduction, but more recent studies have used intracoronary infusion and retrograde coronary sinus infusion to achieve more global transduction of the heart. These new delivery methods bring along new challenges to achieve efficacious transduction for angiogenetic therapies.

**Fig. 26:**
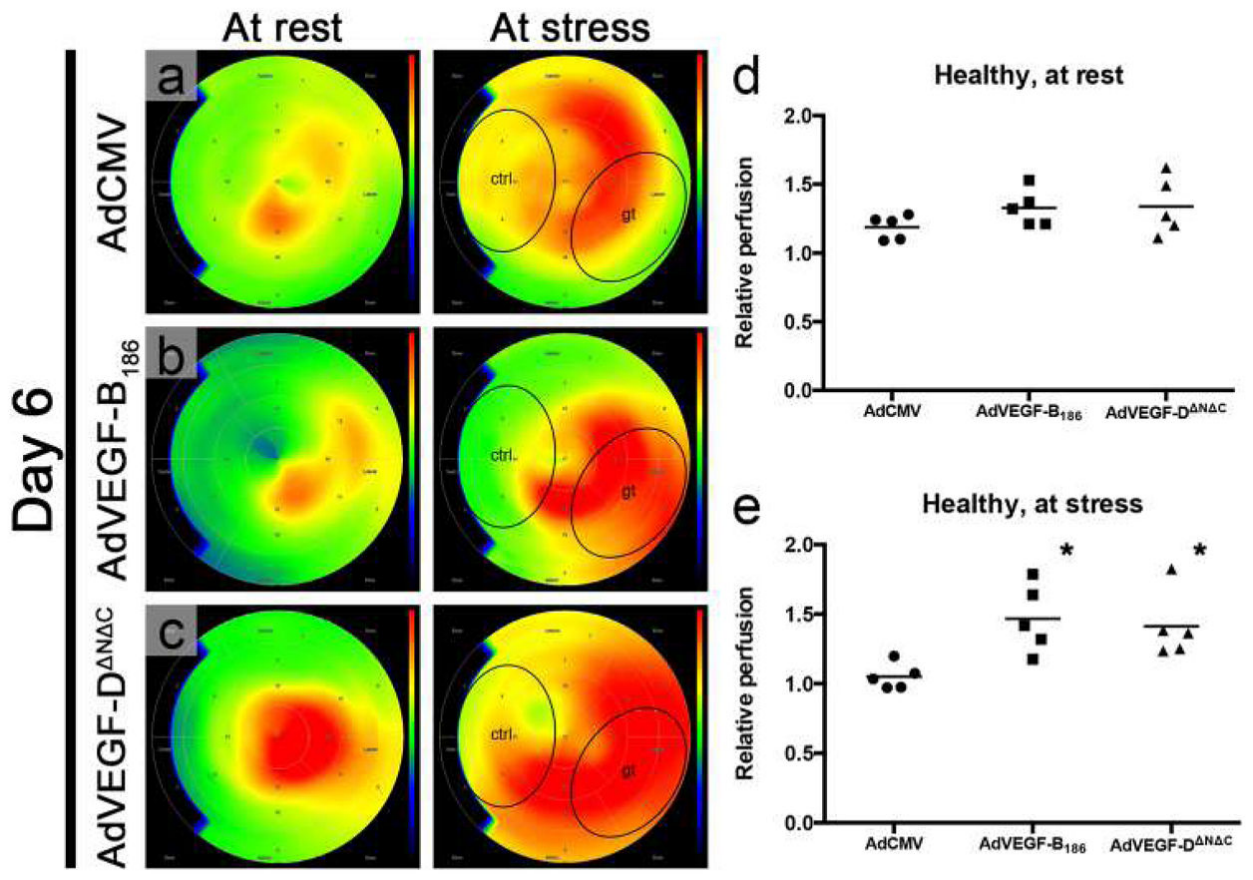
Perfusion imaging is crucial in detecting functional changes in the vasculature. In normoxic pig myocardium 6 days after intramyocardial AdVEGF-B_186_ and AdVEGF-D^ΔNΔC^ gene transfer, myocardial perfusion is increased at stress conditions in the treated region (gt) as measured with PET. Color scale is absolute; darkest blue is 0 ml/min/g, green is 1.5 ml/min/g, and deepest red is 3.0 ml/min/g or over (**a**–**c**). In control AdCMV group, relative perfusion, i.e., the ratio of absolute perfusion in the gene transfer area to that of the control anteroseptal area, did not increase at stress, whereas with both AdVEGF-B_186_ and AdVEGF-D^ΔNΔC^, the relative perfusion was higher in comparison with the control group. Relative perfusion at rest was 12 and 13% and at stress 40 and 34% higher for AdVEGF-B_186_ and AdVEGF-D^ΔNΔC^, respectively **d**, **e**. Ad, adenoviral; CMV, cytomegalovirus; ctrl, control anteroseptal area; gt, posterolateral gene transfer area [654]

In large animal pro-angiogenic studies, study endpoints must be carefully decided. Therapeutic angiogenesis itself is not the de facto aim. These neovessels should be functional and enhance myocardial function. Therefore, functional analyses should have a significant role in evaluating the effects of pro-angiogenic therapies. Previously, microspheres have been used for the evaluation of myocardial perfusion, but with improved technology, imaging modalities such as computed tomography (CT), magnetic resonance imaging (MRI), and positron emission tomography (PET) should play more prominent roles as real-time perfusion, anatomical and functional imaging can be performed simultaneously (Fig. 26). These modalities also allow for a long-term follow-up, as imaging can be performed sequentially, providing a better understanding of the long-lasting effects of the treatments.

Potential limitation of angiogenic therapies is the potential effect of the treatment on neural networks of the heart. It is entirely possible that the pro-angiogenic therapies also induce alterations in nerves producing arrhythmias. As a result, potential sudden deaths and other potential arrhythmia-based problems should be carefully monitored. Also, large animals do not have comorbidities that the patients might possess. For instance, reactions to angiogenic stimuli significantly vary in the ischemic and normoxic myocardium. Rapid growth of heart also causes challenges, as relatively young tissue is more resistant to injury and can regenerate better than tissues in elderly patients.

### 29.6 Concluding remarks

Large animal models are invaluable in neovascularization studies, as they most closely resemble human patients. The model must be carefully selected, and further thought must be given when deciding suitable endpoints, which should nowadays include functional imaging studies.

## 30 Guidelines for purity of recombinant proteins in angiogenesis assays

This brief section aims to provide a few practical guidelines for purity requirements of recombinant proteins. Indeed, many proteins are tested in the in vitro and in vivo angiogenesis assays described throughout this article, including pro- and anti-angiogenic cytokines or growth factors and antibodies. Antibodies, owing to their long systemic half-life, are widely used in preclinical in vivo models. While in the majority of cases reliable commercial sources ensure high quality of reagents and hence reproducibility of experimental results, at times controversial or unexpected findings have emerged that may be, at least in part, traced back to purity and quality control issues.

### 30.1 Endotoxin

One of the major confounders and a key source of variability, both in vitro and in vivo, is represented by endotoxin or lipopolysaccharide (LPS) contamination [627, 628]. LPS is an integral part of the outer cell membrane of Gram-negative bacteria and is released following bacterial lysis. LPS consists of a lipid component, known as Lipid A, a core oligosaccharide and a long heteropolysaccharide chain, the O-specific chain, representing the surface antigen (O-antigen). The O-antigen is strain specific. Lipid A is the most conserved component of endotoxin and is responsible for the majority of the biological effects attributed to LPS [628, 629]. LPS is heat stable and is not destroyed under regular sterilizing conditions. The limulus amebocyte lysate (LAL) assay is commonly used for the detection of endotoxin, and LAL kits are available from many manufacturers. This assay is based on a cell lysate of the horseshoe crab Limulus polyphemus that coagulates in the presence of even very low levels of endotoxins. Endotoxin levels are expressed as units (EU): 1 EU is generally equivalent to 100 pg of LPS.

It is now well established that LPS interacts with toll-like receptor(TLR)-4 [630]. This is a key pathway in innate immunity [631]. Upon activation, TLRs signal as dimers, complexing via their intracellular Toll/interleukin-1 receptor (TIR) domains with a family of adaptor proteins in TLR-specific patterns. This results in the activation of multiple downstream pathways in a cell-type specific fashion [632]. It is also well established that, besides LPS, a variety of endogenous and exogenous ligands can have agonistic or antagonistic effects on TLR4, accounting for a wide variety of biological effects [631].

### 30.2 Effects of endotoxin in angiogenesis assays

LPS can have diverse effects in angiogenesis-related assays. It can induce both cell injury and activation in cultured endothelial cells, even at very low concentrations [633, 634]. In vivo, LPS has been reported to induce tumor angiogenesis [635] and metastasis as well as tumor invasion [636]. On the other hand, a variety of studies have shown the opposite, i.e., anticancer effects of LPS in mouse models and in humans [637]. Indeed, as above noted, TLR4 stimulation can activate multiple signaling cascades, including the MAP kinase and NF-kB pathways [632]. These pathways may activate the secretion of proinflammatory cytokines, such as IL-6 and IL-8, or anti-inflammatory type I IFNs, including IFN-γ. To further complicate the picture, it has been reported that, depending on p53 status, TLR4 stimulation may have opposite effects on promotion of breast cancer cell growth [638].

A common misconception is that only proteins expressed in bacteria can be contaminated by LPS. In reality, a variety of sources have been found to be contaminating [628]. Tap water, air and people’s fingers can lead to endotoxin contamination. It has been reported that one of the most common sources of endotoxin is laboratory water since distillation and deionizing columns do not remove endotoxin [628]. Pyrogen-free water is helpful, but LPS can be still introduced by inadequate handling of containers during washing procedures. A variety of chemical reagents and buffers are also potential sources of endotoxin [628]. It is not uncommon to find high levels of endotoxin in biological products such as albumin, collagen or gelatin. Indeed, it has been argued that, unless specific steps to prevent LPS contamination have been taken and the LPS level is indicated, one should assume that a reagent is almost certainly contaminated [628]. Therefore, LPS levels ideally should be tested routinely and certainly whenever unexpected results are obtained.

In biotech settings, the generally acceptable LPS levels for antibodies or other protein reagents to be tested in pre-clinical studies are < 1 EU (~ 100 pg)/mg protein. Achieving such low levels requires significant planning and taking multiple steps to prevent contamination [628], besides the usual chromatographic steps aimed at achieving high purity of the recombinant protein. Treatment of columns and equipment with 1 N NaOH for 1 h (> 10 h when using 0.1 N NaOH) is typically employed to inactivate LPS. Methods to remove LPS in contaminated preparations have also been described, although their success is relatively mixed [629].

It is noteworthy that even some relatively recent controversies in the tumor angiogenesis field might be attributed, at least in part, to LPS (or other contaminants). For example, Paez-Ribes and colleagues reported in 2009 the unexpected finding that the anti-VEGFR2 monoclonal antibody DC101 promotes invasiveness and metastasis in the Rip-Tag model of insulinoma [530]. This finding was in conflict with previous studies reporting inhibition of invasiveness by DC101 in other models [639] and with subsequent reports that did not confirm induction of tumor invasiveness and metastasis by DC101 in the Rip-Tag model [557, 640]. While the DC101 employed in the majority of studies was purified and distributed by ImClone Systems [641], Paez-Ribes and colleagues purified the antibody themselves from supernatants of hybridoma cells that had been deposited at the American Tissue Culture Collection [530]. Unfortunately, the authors did not describe any steps to remove LPS nor did they report the LPS levels in their preparations of DC101 [530]. Given the high doses of DC101 required for in vivo studies (100 mg/kg/week), it is conceivable that even a relatively modest contamination may affect the results. It remains to be determined whether LPS was truly responsible for the reported tumor invasiveness.

**Fig. 27:**
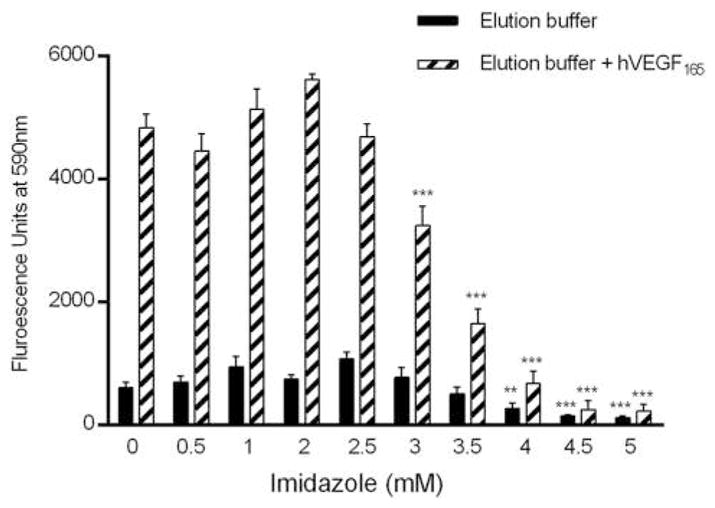
Effects of imidazole on endothelial cell proliferation. BCECs (bovine choroidal microvascular endothelial cells) were cultured in the presence of low-glucose DMEM in the presence of 10% bovine calf serum as previously described [645]. Elution buffer was tested up to a final concentration of 5 mM imidazole, with or without 10 ng/ml VEGF165. Note that the concentration of imidazole typically used to elute His-tagged proteins is 500 mM. After 5–6 days, cell proliferation was determined by fluorescence readings at 590 nm. Asterisks denote significant differences compared to no addition (0) groups by *t* test (***p < 0.001, **p < 0.01)

However, LPS is by no means the only confounder in angiogenesis-related assays. Commonly used reagents or contaminants, for example detergents employed to solubilize samples or misfolded or aggregated proteins, can have major effects on endothelial cells. In the course of earlier studies in which we screened libraries of secreted proteins to identify growth stimulators and inhibitors [642], we observed a surprisingly high rate of inhibitory “hits” using an early version of the library [643]. However, the majority of these “hits” could not be confirmed using more stringently purified proteins. It became apparent that basal or VEGF-stimulated endothelial cell growth could be easily inhibited by a variety of impurities, as above noted. Imidazole, the buffer typically employed to elute His-tagged protein bound to Nickel columns, a common procedure to purify recombinant proteins, was also found to have profound effects in endothelial cell proliferation assays. Figure 27 illustrates the effects of imidazole on proliferation of bovine microvascular endothelial cells. As little as 3 mM significantly inhibited VEGF-stimulated endothelial cell growth. At 4–5 mM imidazole, there was a dramatic inhibition of both basal and VEGF-stimulated endothelial cell growth. Considering that 500 mM is the concentration of imidazole commonly used to elute proteins from Nickel columns, a several hundred-fold final dilution or, preferably, a buffer exchange is advisable before adding the sample to endothelial cells. It has been suggested that similar issues might account for the opposite reports by two groups on the effects on endothelial cell growth of VEGF-Ax, a VEGF isoform arising from read through translation [644, 645].

### 30.3 Concluding remarks

It is clear from the above that contamination of reagents can have major effects in both in vitro and in vivo assays. The most well-known contaminant with major consequences is bacterial endotoxin, or LPS. Endotoxin is not only present in recombinant preparations but can be co-purified from many other sources. Next to endotoxin, other contaminants can have major effects as well. It is therefore required to check compound preparations for the presence of contaminants and take action for purification when necessary.

## 31 Conclusions

This paper describes a large number of assays and analysis methods that can be used for the assessment of angiogenesis. A proper interpretation of these assays is warranted for obtaining relevant mechanistical insight into the sequence of angiogenesis processes in different tissues and under changing conditions. We have aimed to provide insight into the use and interpretation of the mostly used assays and have tried to provide the reader with the challenges, limitations, and pitfalls in making use of these assays.

## Figures and Tables

**Table 1:** Comparison of 3D models of vascular morphogenesis

Assay	Process					

	Sprouting	Lumen formation	Anastomosis	Pericyte recruitment	Perfusion	Pruning
Fibrin bead assay	✓	✓	✓	✓		
Collagen lumen assay		✓		✓		
Retina explant assay	✓	✓	✓			
Vascularized micro-organ	✓	✓	✓	✓	✓	✓

**Table 2:** Variety of applications with the CAM

	References
*Developmental angiogenesis*	
Differentiation of vascular endothelium	[352, 655]
Membrane proteome associated with the vasculature	[366]
Gene transfer/global gene expression	[654–658]
Metabolic profiling	[659]
Transcriptome analysis in the “wound model”	[660]
Vascular and endothelial cell targets from isolated chicken membranes	[367]
*Lymphangiogenesis*	
Prox-1 in the lymphatic endothelial cells	[357, 661]
Ingrowth of lymphatics into the tumors	[662]
Embryonic lymphangiogenesis	[663]
*Vasomodulating therapies*	
Radiosensitizing activity	[662–666]
Microbeam radiation therapy	[667]
Photodynamic therapy and diagnosis	[666–676]
*Tumor angiogenesis*	
Tumor growth in the CAM	[348, 362, 363, 677, 678]
Experimental metastasis	[677–682]
Interstitial pO_2_ gradients in solid tumors	[341, 683]
*Accessing molecules activity*	
Growth factor (receptors) inhibitors; endothelium-targeting molecules	[341, 684, 685]
Metal-based compounds	[684–691]
Inflammatory and tumor cells or purified effector molecules	[692]
Pro-angiogenic molecules	[693]
*Stem cells*	
Human mesenchymal stem cells	[692–696]
Human skin-derived stem cells	[697]
*Drug delivery, nanoparticles*	
Drug delivery for cancer treatment	[698, 699]
Visible laser irradiation + gold nanoparticles	[700]
Screening of nanocarrier vehicles	[701, 702]
*Engineering*	
Tissue engineering and biomaterials	[701–706]

**Table 3:** Angiogenesis assays and vascular analysis

	References
*Development of novel imaging techniques*	
Direct measurement of interstitial diffusion and convection of albumin using fluorescence photobleaching	[707]
Tumor induction of VEGF promoter activity in stromal cells	[506]
Interstitial pH and pO_2_ gradients in solid tumors in vivo	[505]
In vivo measurement of gene expression, angiogenesis, and physiological function in tumors	[487]
Two-photon fluorescence correlation microscopy to reveal transport in tumors	[498]
Quantum dots to spectrally distinguish multiple species within the tumor milieu in vivo	[490]
Three-dimensional in vivo microscopy using optical frequency domain imaging	[485]
Simultaneous measurement of RBC velocity, flux, hematocrit, and shear rate in vivo	[495]
In vivo validation of MRI vessel caliber index with intravital optical microscopy in a mouse brain tumor model	[515]
Video-rate resonant scanning multiphoton microscopy	[496]
Next-generation in vivo optical imaging with short-wave infrared quantum dots	[708]
*Analysis of vessel function*	
Regulation of transport pathways in tumor vessels: role of tumor type and microenvironment	[501]
Effect of tumor–host interactions on distal angiogenesis and tumor growth	[709]
Kinetics of vascular normalization in response to VEGFR2 blockade	[510]
*pH/pO_2_*	
Calibration and application of fluorescence ratio imaging of pH gradients	[503]
Noninvasive measurement of microvascular and interstitial oxygen profiles	[504]
Simultaneous in vivo high-resolution measurements of interstitial pH and pO_2_ gradients	[505]
*Extracellular matrix and interstitial transport*	
Fluorescence photobleaching with spatial Fourier analysis for measurement of diffusion	[502]
Dynamic imaging of collagen in tumors in vivo using second-harmonic generation	[491]
*In vivo* imaging of ECM remodeling by tumor-associated fibroblasts	[492]
Angiotensin inhibition enhances drug delivery by decompressing tumor blood vessels	[500]
Anti-VEGF therapy induces ECM remodeling and mechanical barriers to therapy	[481]
*Immune cells*	
VEGF and bFGF regulate natural killer cell adhesion to tumor endothelium	[497]
Ly6C^low^ monocytes drive immunosuppression and confer resistance to anti-VEGFR2 therapy	[511]
*Analysis of lymphatic vessel function*	
Conventional and high-speed intravital multiphoton laser scanning microscopy	[508]
Lymphatic metastasis in the absence of functional intratumor lymphatics	[710]
A genetic Xenopus laevis tadpole model to study lymphangiogenesis	[711]
Investigation of the lack of angiogenesis in the formation of lymph node metastases	[486]
*Drug delivery, nanoparticles*	
Vascular normalization improves the delivery of nanomedicines in a size-dependent manner	[499]
Compact high-quality CdSe-CdS core–shell nanocrystals with narrow emission linewidths and suppressed blinking	[489]
Magneto-fluorescent core–shell supernanoparticles	[712]
*Engineered vasculature*	
Tissue engineering: creation of long-lasting blood vessels	[407]
Paradoxical effects of PDGF-BB overexpression in EC in vivo	[713]
Engineered blood vessel networks connect to host vasculature via wrapping-and-tapping anastomosis	[509]
Generation of functionally competent durable engineered blood vessels from human pluripotent stem cells	[398]
*Mathematical analysis of angiogenesis*	
Scale-invariant behavior and vascular network formation in normal and tumor tissue	[517]
Cancer, angiogenesis, and fractals	[519]
Scaling rules for diffusive drug delivery in tumor and normal tissues	[518]
*Assessing therapies*	
Herceptin acts as an anti-angiogenic cocktail	[714]
Targeting placental growth factor/neuropilin 1 pathway inhibits growth and spread of medulloblastoma	[478]
Vascular endothelial protein tyrosine phosphatase inhibition in tumor vasculature and metastatic progression	[512]
A cerebellar window for intravital imaging of medulloblastoma in mice	[479]
*Tissue isolation chambers*	
Implantable tissue isolation chambers for analyzing tumor dynamics in vivo	[493]

## Abbreviations

AAV: Adeno-associated virus
AMD: Age-related macular degeneration
BME: Basement membrane extracts
BOEC: Blood outgrowth endothelial cells
BrdU: 5-Bromo-2′-deoxyuridine
CAF: Cancer-activated fibroblasts
CAM: Chorioallantoic membrane
CD31: Platelet endothelial cell adhesion molecule
CD45: Leukocyte common antigen
CD144: Vascular endothelial cadherin
CNV: Choroidal neovascularization
CRC: Colorectal carcinoma
CT: Computer tomography
D: Definitive differentiated cells
DAPI: 4′,6-Diamidino-2-phenylindole
EACA: Epsilon aminocaproic acid
EBM: Endothelial basal medium
EC: Endothelial cell(s)
ECAR: Extracellular acidification rate
ECFC: Endothelial colony-forming cells
ECM: Extracellular matrix
EdU: 5-Ethynyl-2′deoxyuridine
ELISA: Enzyme-linked immunosorbent assay
ENU: *N*-ethyl-*N*-nitrosourea
EPC: Endothelial progenitor cells
EST: Expressed sequence tag
EVP: Endovascular progenitor cells
FGF: Fibroblast growth factor
FACS: Fluorescence-activated cell sorting
GFP: Green fluorescent protein
GS-B_4_: Griffonia simplicifolia isolectin B_4_
HDMEC: Foreskin-derived human dermal microvascular EC
H&E: Hematoxylin and eosin
HIF: Hypoxia-inducible factor
HILIC: Hydrophilic liquid chromatography
hiPSC: Human-induced pluripotent stem cell
HGP: Histopathological growth pattern
HSVSMC: Saphenous vein smooth muscle cells
HUAEC: Human umbilical artery EC
HUVEC: Human umbilical vein EC
ICAM: Intercellular adhesion molecule
IL-3: Interleukin 3
LDPI: Laser Doppler perfusion imaging
LPS: Lipopolysaccharide
MACS: Magnetic-activated cell sorting
MDSC: Mye-loid-derived suppressor cells
MMP: Matrix metal-loproteinase
MO: Morpholino
MP: Main population
MPC: Mesenchymal progenitor cells
MRI: Magnetic resonance imaging
MSC: Mesenchymal stem cells
MTT: 3-(4,5-Dimethylthiazol-2-yl)-2,5-diphenyltetrazolium bromide
NMR: Nuclear magnetic resonance
NV: Neovascularization
OCR: Oxygen consumption rate
OIR: Oxygen-induced retinopathy
oxPPP: Oxidative pentose phosphate pathway
PAD: Peripheral arterial disease
PCNA: Proliferating cell nuclear antigen
PCR: Polymerase chain reaction
PCV: Polypoidal choroidal vasculopathy
PDMS: Polydimethylsiloxane
PET: Positron emission tomography
PI: Propidium iodide
PNET: Pancreatic neuroendocrine tumor
PV: Particle velocimetry
PWG: Postnatal weight gain
RCC: Renal cell carcinoma
ROP: Retinopathy of prematurity
RSII: Reduced serum supplement II
RTCA: Real-time cell analysis
SCF: Stem cell factor
SCID: Severe combined immune deficiency
SDF: Stromal-derived factor
SEM: Scanning electron microscopy
SMA: Smooth muscle actin
SOD: Super-oxide dismutase
SP: Side-population
SPARC: Secreted protein acidic and rich in cysteine
TA: Transient amplifying cells
TAM: Tumor-associated macrophages
TCA: Tetracarboxylic acid
TEM: Tumor endothelial marker
TLR: Toll-like receptor
TME: Tumor micro-environment
TNF: Tumor necrosis factor
UEA: Ulex europaeus agglutinin
VCAM: Vascular cell adhesion molecule
VEGF: Vascular endothelial cell growth factor
VESC: Vascular endothelial stem cells
VHL: Von Hippel–Lindau
VMO: Vascularized micro-organ
VO: Vaso-obliteration
WGA: Wheat germ agglutinin
